# Perioperative Adriamycin plus ifosfamide vs. gemcitabine plus docetaxel for high-risk soft tissue sarcomas: randomised, phase II/III study JCOG1306

**DOI:** 10.1038/s41416-022-01912-5

**Published:** 2022-07-23

**Authors:** Kazuhiro Tanaka, Ryunosuke Machida, Akira Kawai, Robert Nakayama, Satoshi Tsukushi, Kunihiro Asanuma, Yoshihiro Matsumoto, Hiroaki Hiraga, Koji Hiraoka, Munenori Watanuki, Tsukasa Yonemoto, Satoshi Abe, Hirohisa Katagiri, Yoshihiro Nishida, Akihito Nagano, Yoshiyuki Suehara, Hiroyuki Kawashima, Masanori Kawano, Takeshi Morii, Hiroshi Hatano, Junya Toguchida, Tomotake Okuma, Masanobu Takeyama, Satoshi Takenaka, Toshihiro Akisue, Taisuke Furuta, Makoto Emori, Toru Hiruma, Hidetatsu Outani, Tetsuji Yamamoto, Tomoko Kataoka, Haruhiko Fukuda, Toshifumi Ozaki, Yukihide Iwamoto

**Affiliations:** 1 Department of Orthopaedic Surgery, Oita University, Oita, Japan; 2 Japan Clinical Oncology Group Data Center/Operations Office, National Cancer Center Hospital, Tokyo, Japan; 3 Division of Musculoskeletal Oncology, National Cancer Center Hospital, Tokyo, Japan; 4 Department of Orthopaedic Surgery, Keio University School of Medicine, Tokyo, Japan; 5 Department of Orthopaedic Surgery, Aichi Cancer Center Hospital, Nagoya, Japan; 6 Department of Orthopaedic Surgery, Mie University, Mie, Japan; 7 Department of Orthopaedic Surgery, Kyusyu University, Fukuoka, Japan; 8 Department of Orthopaedic Surgery, Hokkaido Cancer Center, Sapporo, Japan; 9 Department of Orthopaedic Surgery, Kurume University, Kurume, Japan; 10 Department of Orthopaedic Surgery, Tohoku University Hospital, Sendai, Japan; 11 Department of Orthopaedic Surgery, Chiba Cancer Center, Chiba, Japan; 12 Department of Orthopaedic Surgery, Teikyo University, Tokyo, Japan; 13 Department of Orthopaedic Surgery, Shizuoka Cancer Center, Shizuoka, Japan; 14 Department of Orthopaedic Surgery, Nagoya University, Nagoya, Japan; 15 Department of Orthopaedic Surgery, Gifu University, Gifu, Japan; 16 Department of Orthopaedic Surgery, Juntendo University, Tokyo, Japan; 17 Department of Orthopaedic Surgery, Niigata University, Niigata, Japan; 18 Department of Orthopaedic Surgery, Kyorin University, Tokyo, Japan; 19 Department of Orthopaedic Surgery, Niigata Cancer Center Hospital, Niigata, Japan; 20 Department of Orthopaedic Surgery, Kyoto University, Kyoto, Japan; 21 Department of Musculoskeletal Oncology, Tokyo Metropolitan Cancer and Infectious Diseases Center Komagome Hospital, Tokyo, Japan; 22 Department of Orthopaedic Surgery, Yokohama City University, Kanagawa, Japan; 23 Musculoskeletal Oncology Service, Osaka International Cancer Institute, Osaka, Japan; 24 Department of Orthopaedic Surgery, Kobe University, Kobe, Japan; 25 Department of Orthopaedic Surgery, Hiroshima University, Hiroshima, Japan; 26 Department of Orthopaedic Surgery, Sapporo Medical University, Sapporo, Japan; 27 Department of Musculoskeletal Tumor Surgery, Kanagawa Cancer Center, Kanagawa, Japan; 28 Department of Orthopaedic Surgery, Osaka University, Osaka, Japan; 29 Department of Orthopaedic Surgery, Kagawa University, Kagawa, Japan; 30 Department of Orthopaedic Surgery, Okayama University, Okayama, Japan; 31 Kyushu Rosai Hospital, Kitakyushu, Japan

**Keywords:** Sarcoma, Sarcoma

## Abstract

**Background:**

This randomised phase II/III trial aimed to determine whether perioperative chemotherapy with gemcitabine plus docetaxel (GD) is non-inferior to the standard Adriamycin plus ifosfamide (AI) in terms of overall survival (OS) in patients with soft tissue sarcoma (STS).

**Methods:**

Patients with localised high-risk STS in the extremities or trunk were randomised to receive AI or GD. The treatments were repeated for three preoperative and two postoperative courses. The primary endpoint was OS.

**Results:**

Among 143 enrolled patients who received AI (70 patients) compared to GD (73 patients), the estimated 3-year OS was 91.4% for AI and 79.2% for GD (hazard ratio 2.55, 95% confidence interval: 0.80–8.14, *P* = 0.78), exceeding the prespecified non-inferiority margin in the second interim analysis. The estimated 3-year progression-free survival was 79.1% for AI and 59.1% for GD. The most common Grade 3–4 adverse events in the preoperative period were neutropenia (88.4%), anaemia (49.3%), and febrile neutropenia (36.2%) for AI and neutropenia (79.5%) and febrile neutropenia (17.8%) for GD.

**Conclusions:**

Although GD had relatively mild toxicity, the regimen—as administered in this study—should not be considered a standard treatment of perioperative chemotherapy for high-risk STS in the extremities and trunk.

**Clinical trial registration:**

jRCTs031180003.

## Introduction

Soft tissue sarcoma (STS) is a group of malignant tumours with >50 histological subtypes [1]. STS accounts for <1% of all malignancies and only approximately 13,000 cases are diagnosed annually in the United States [2]. The incidence is even lower in Japan, with only approximately 2,000 STS cases registered during 2019 in the Japanese Orthopaedic Association’s registry of soft tissue tumours [3].

The standard treatment for localised STS is surgical resection [4, 5], although recurrence and metastases occur in approximately one-half of localised STS cases. Therefore, the effectiveness of adjuvant chemotherapy combined with surgery has been investigated. The Italian Sarcoma Group (ISG) conducted a randomised controlled trial (RCT) that demonstrated superior overall survival (OS), relative to surgery alone, with surgery and adjuvant chemotherapy using full-dose epirubicin plus ifosfamide (IFM, the EI regimen) to treat high-risk STS, which was defined as high-grade deeply located tumours that were >5 cm [6]. Although the largest RCT to compare adjuvant chemotherapy using Adriamycin (ADM) plus IFM (the AI regimen) and surgery alone for STS did not show significant difference in survival [7], a meta-analysis of 18 RCTs revealed that surgery plus adjuvant AI chemotherapy for STS provided a significant improvement in OS (11% reduction in the risk of death) [8]. The ISG further conducted a phase III study with a larger number of STS patients and validated the efficacy of preoperative chemotherapy using full-dose EI for high-risk STS in the extremities and trunk [9]. The Bone and Soft Tissue Tumor Study Group (BSTTSG) of the Japan Clinical Oncology Group (JCOG) also conducted a phase II trial (JCOG0304) of perioperative chemotherapy using full-dose AI for high-risk STS in the extremities, which revealed favourable OS [10] and good long-term survival at the 10-year follow-up, which was similar to the long-term results from the ISG study [11, 12].

Although perioperative AI appears to be effective for patients with STS, this regimen is highly toxic. Severe haematological toxicities were almost inevitable (Grade 3–4 leukopenia: 97.2%, Grade 3–4 neutropenia: 98.6%) in the JCOG0304 trial [10]. Thus, given that STS patients are generally old, a less toxic treatment option would be desirable. In this context, gemcitabine (GEM) plus docetaxel (DOC, the GD regimen) is effective for advanced STS and has relatively mild toxicity. A randomised phase II trial comparing GEM alone to GD revealed that the GD regimen provided superior progression-free survival (PFS) and OS among patients with advanced STS [13]. A randomised phase II trial also compared perioperative chemotherapy using AI or GD for localised STS, which revealed that GD was associated with significantly better disease-free survival (DFS) [14]. Therefore, the JCOG BSTTSG conducted a randomised phase II/III trial (JCOG1306) that aimed to confirm the non-inferiority of perioperative GD to AI for localised high-risk STS in the extremities or trunk [15].

## Methods

### Patients

JCOG1306 was a multicentre two-arm open-label randomised phase II/III trial. The definition of localised high-risk STS was high-grade tumours that were >5 cm and deeply located beyond the investing fascia, tumours with lymph node metastasis, or recurrent tumours. The major inclusion criteria were (1) histologically proven STS based on the 2013 World Health Organisation classification subtypes [16] and Grade 2–3 according to the Federation Nationale des Centers de Lutte Contre le Cancer system [17]: undifferentiated pleomorphic sarcoma (UPS), fibrosarcoma, myxofibrosarcoma, leiomyosarcoma, synovial sarcoma, liposarcoma, pleomorphic rhabdomyosarcoma, malignant peripheral nerve sheath tumour, angiosarcoma, or unclassified sarcoma; (2) primary tumour classified as T2bN0M0 or anyTN1M0 (American Joint Committee on Cancer, 7th edition) [18]; (3) no metastasis for recurrent tumours; (4) resectable tumours in the extremities or trunk; (5) age of 20–70 years; (6) Eastern Cooperative Oncology Group performance status of 0–1; (7) no history of chemotherapy or radiotherapy; and (8) sufficient bone marrow function (neutrophils: ≥1500/µL, platelets: ≥100,000/µL, and haemoglobin: ≥8.0 g/dL), renal function (creatinine: ≤1.5 mg/dL), and hepatic function (total bilirubin: ≤1.5 mg/dL and transaminases: ≤100 IU/L). Patients were also required to have normal electrocardiography findings (or at least no changes requiring treatment) and no signs of interstitial pneumonitis or severe emphysema by chest computed tomography (CT). The histological classification was based on a central review of the biopsy specimens by the BSTTSG Central Pathologic Committee (three pathologists who specialise in STS).

The major exclusion criteria were (1) synchronous or metachronous malignancies; (2) active infection requiring systemic therapy; (3) systemic steroid treatment; (4) unstable angina or history of myocardial infarction; and (5) poorly controlled diabetes mellitus. Other inclusion and exclusion criteria are described in the Supplemental Appendix.

All patients provided written informed consent before enrolment. The JCOG1306 protocol was approved by the participating institutional review boards.

### Randomisation and masking

Patients were randomly assigned (1:1) to receive AI or GD treatment using a minimisation method for balancing institution, recurrence status (primary vs. recurrence), and tumour location (extremities vs. trunk). The randomisation procedure was performed centrally by the JCOG Data Centre and the treatment allocation was performed after patients completed an online registration process. Masking of the treatment allocation was not possible because of the clear differences in the treatment schedules and toxicities.

### Procedure

The protocol treatments involved three courses of preoperative chemotherapy, surgery, and two courses of postoperative chemotherapy. The AI regimen involved ADM (30 mg/m^2^/day, 2-h intravenous infusions on days 1–2) and IFM (2 g/m^2^/day, 4-h intravenous infusions on days 1–5). The GD regimen involved GEM (900 mg/m^2^/day, 30-min intravenous infusions on day 1 and day 8) and DOC (70 mg/m^2^/day, 1-h intravenous infusion on day 8). The assigned chemotherapy regimen was repeated every 3 weeks.

The surgical technique aimed to provide wide or marginal surgical margins. When the surgical margin was considered insufficient, radiotherapy was performed after the protocol treatment at the discretion of the treating physicians. Primary prophylaxis using granulocyte colony-stimulating factor was not allowed, although secondary prophylaxis was provided for all subsequent courses if a patient developed Grade 4 leukopenia or neutropenia that lasted for ≥5 days.

Adverse events were assessed according to the National Cancer Institute Common Terminology Criteria for Adverse Events version 4.0. Each chemotherapy course was started when all per-protocol criteria regarding haematological and non-haematological adverse events were satisfied (Supplemental Appendix). The radiological response to preoperative chemotherapy was evaluated using magnetic resonance imaging (MRI) after the last course of the preoperative chemotherapy according to the Response Evaluation Criteria in Solid Tumors (RECIST) version 1.1 [19]. The histological response was also evaluated using the surgical specimen as previously described [10]. Based on tumour necrosis in the resected specimen, response was histologically classified as Grade 1 (<50% of the tumour), Grade 2 (50–90% necrosis), Grade 3 (>90% necrosis), and Grade 4 (no viable tumour cells). For the present study, a histological response was categorised as Grades 3–4. Disease status was monitored every 3 months for ≥5 years after completing patient accrual. Chest CT or radiography was performed every 3 months, and chest CT or MRI was performed every 6 months. Local MRI was strongly recommended every 3 months for all patients.

After the completion of the protocol treatment, no additional therapy was allowed until the patient experienced local recurrence and/or distant metastasis, except in cases that involved radiotherapy because of insufficient surgical margins. No treatment restrictions were imposed after disease progression.

### Outcomes

The phase II primary endpoint was defined as the proportion of the GD group that completed preoperative chemotherapy without disease progression (i.e. completed three courses of preoperative GD chemotherapy with a RECIST response of complete response, partial response, or stable disease). The phase III primary endpoint was OS, and the secondary endpoints were PFS, radiological and histological response, limb preservation, disease control, and toxicities. The OS was calculated from randomisation to death because of any cause, and the PFS was calculated from randomisation to the first instance of disease progression or death because of any cause. Radiological response was evaluated according to RECIST, and histological response was evaluated by the proportion of viable tumour cells in the resected specimen after preoperative chemotherapy. For histological response rates, patients who did not undergo surgery were included in the denominator. Limb preservation was defined as the proportion of patients who were able to preserve limb after surgery among all patients with tumours in the extremities. Disease control was evaluated as the proportion of CR, PR, or SD after all registered patients received preoperative chemotherapy.

### Statistical analysis

The trial aimed to evaluate the efficacy and safety of perioperative GD chemotherapy in the phase II part and to confirm the non-inferiority of GD to AI in terms of OS in the phase III part. No clinical trial had evaluated perioperative GD for localised STS at the start of the present study, which suggested that a phase II trial was prudent to determine whether perioperative GD should be evaluated in the confirmatory phase III trial. Thus, we selected a phase II/III design.

The planned phase II sample size was 28 patients in the GD group, which was calculated based on an expected value of 85% and a threshold of 65% for the proportion of patients to complete preoperative chemotherapy without disease progression, with one-sided alpha of 10% and power of 80%. The planned phase III sample size was set at 140 patients (70 patients in each group) to observe the required number of events of 33 based on Schoenfeld and Richter’s method [20] with enrolment of 6 years, follow-up of 3 years, power of 70%, and non-inferiority margin for hazard ratio (HR) of 1.61, expecting 3-year OS of 85% in AI group and that of 87% in GD group. The significant level (one-sided) was set at 10% in consideration of the rarity of STS.

Two interim analyses were planned. The first interim analysis (i.e., analysis of phase II part) was conducted after phase II accrual of 28 patients in the GD group to determine whether to proceed to the phase III part. The second interim analysis was conducted after the patient accrual and protocol treatments had been completed. The Lan–DeMets method with the O’Brien and Fleming alpha spending function was used to adjust the multiplicity of the tests in the second interim analysis and primary analysis to keep the one-sided alpha of 10% throughout the trial [21]. Details of the stopping guideline are described in the Supplemental Appendix. The JCOG Data and Safety Monitoring Committee (DSMC) independently reviewed the interim analysis reports, and in-house monitoring was performed every 6 months by the JCOG Data Center.

In the phase II part, the proportion of completing preoperative chemotherapy without disease progression in the GD group and 80% confidence interval (CI) were estimated. The CI was calculated using exact method based on binomial distributions. In the phase III part, data from all randomly assigned patients were analysed for OS and PFS on an intention-to-treat basis. The HR and CI for OS were estimated using a Cox proportional hazard model stratified according to recurrence status (primary vs. recurrence) and tumour location (extremities vs. trunk). Although a stratified analysis above had been prespecified for the primary analysis for OS in the protocol, we noticed that one of four strata had no OS event under masked conditions at the second interim analysis. Thus, we conducted the unstratified analysis to estimate HR at the second interim analysis according to the statistical analysis plan, which was prepared under masked conditions before the confirmatory analysis with comparison between groups. The HR and 95% CI for PFS were estimated using an unstratified Cox proportional hazard model. OS and RFS were estimated using the Kaplan–Meier method. The radiological response rate, histological response rate, disease control rate, and 95% CIs were estimated in each group. The CIs were calculated using exact method based on binomial distributions. Safety was assessed on a per-protocol basis. All statistical analyses were conducted by the JCOG Data Center using SAS software, version 9.4. The trial protocol was also registered in the Japan Registry of Clinical Trials (jRCTs031180003).

## Results

**Fig. 1:**
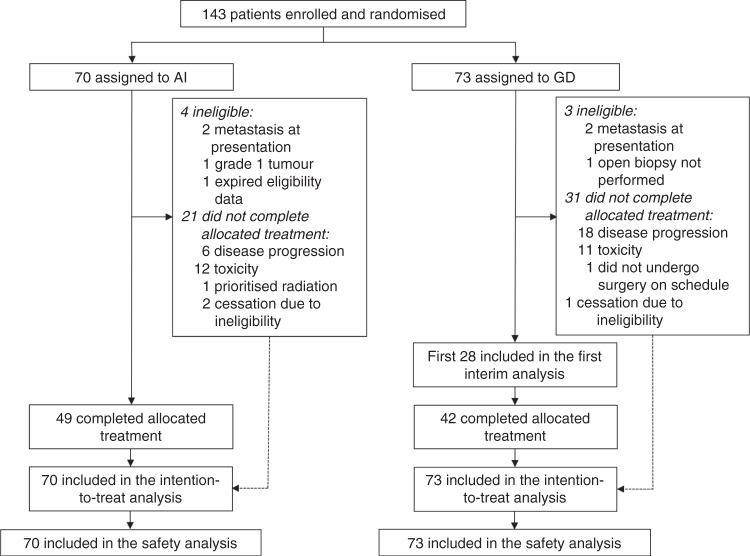
CONSORT diagram.

Between February 17, 2014 and September 28, 2018, 143 patients were enrolled (AI group: 70 patients, GD group: 73 patients) (Fig. 1). Four patients in the AI group were deemed ineligible because of metastasis at presentation (two patients), a Grade 1 tumour (one patient), and expired eligibility data (one patient). Three patients in the GD group were deemed ineligible because of metastasis at presentation (two patients) and mandatory open biopsy was not performed (one patient). However, these patients received the assigned treatments and were included in the intention-to-treat analysis.

The patients’ basic characteristics are summarised in Table 1. Most characteristics were balanced between the two treatment groups, although imbalances were observed in terms of tumour grade and clinical stage. Grade 2 tumours were observed for 48 patients in the AI group and 37 patients in the GD group, while Grade 3 tumours were observed for 22 patients in the AI group and 36 patients in the GD group. Clinical stage IIB was assigned for 44 patients in the AI group and 35 patients in the GD group, while stage III was assigned for 24 patients in the AI group and 36 patients in the GD group. Imbalances were also observed for some histological subtypes, including liposarcoma, UPS, and leiomyosarcoma.

**Table 1:** Patient characteristics.

	AI (*n* = 70)	GD (*n* = 73)
*Age in years, median (IQR; range)*	55.5 (46–62; 25–70)	54.0 (43–61; 24–70)
*Sex (male/female)*	39 (56%)/31 (44%)	42 (58%)/31 (42%)
*ECOG performance status (0/1)*	60 (86%)/10 (14%)	62 (85%)/11 (15%)
*Histological subtype*		
Liposarcoma	21 (30%)	16 (22%)
Myxoid liposarcoma	11 (16%)	7 (10%)
Dedifferentiated liposarcoma	7 (10%)	7 (10%)
Pleomorphic liposarcoma	3 (4%)	2 (3%)
Undifferentiated pleomorphic sarcoma	13 (19%)	22 (30%)
Synovial sarcoma	11 (16%)	9 (12%)
Myxofibrosarcoma	9 (13%)	9 (12%)
Leiomyosarcoma	3 (4%)	7 (10%)
Others	13 (19%)	10 (14%)
*Site (extremity/trunk)*	62 (89%)/8 (11%)	61 (84%)/12 (16%)
*Tumour status (primary/recurrent)*	69 (99%)/1 (1%)	70 (96%)/3 (4%)
*Lymph node (N0/N1)*	68 (97%)/2 (3%)	71 (97%)/2 (3%)
*Tumour size in cm, median (IQR; range)*	9.0 (6.6–12.6; 5.0–26.5)	10.2 (7.5–12.1; 3.7–32.0)
*Histological grade (2/3)*	48 (69%)/22 (31%)	37 (51%)/36 (49%)
*AJCC Stage (IIB/III/IV)*	44 (63%)/24 (34%)/2 (3%)	35 (48%)/36 (49%)/2 (3%)

Data are *n* (%) unless noted otherwise.

*AI* Adriamycin plus ifosfamide, *GD* gemcitabine plus docetaxel, *AJCC* American Join Committee on Cancer, *ECOG* Eastern Cooperative Group.

The first interim analysis was performed on April 15, 2016 (28 patients in the GD group had completed preoperative chemotherapy). The RECIST responses were stable disease for 22 patients, progressive disease for five patients, and not evaluable for one patient. All 22 patients who achieved stable disease completed three courses of preoperative chemotherapy. The proportion of completing preoperative chemotherapy without disease progression (phase II primary endpoint) in the GD group was 78.6% (22/28 patients; 80% CI: 65.4–88.3%), which exceeded the prespecified threshold of 65%. Thus, the JCOG DSMC allowed us to proceed with the phase III part of the study.

**Fig. 2:**
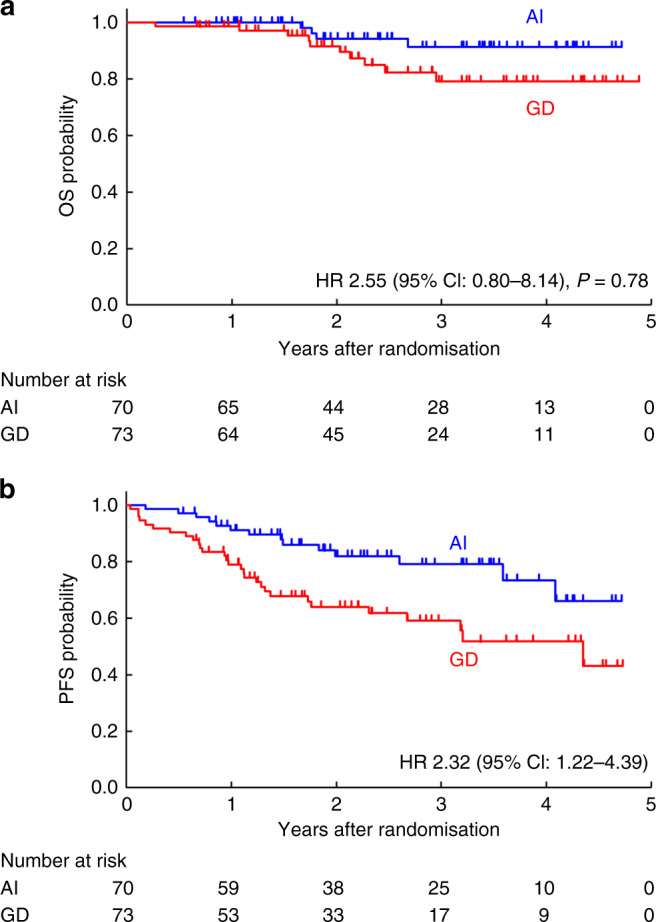
Overall survival and progression-free survival. Overall survival (**a**) and progression-free survival (**b**) among patients randomly assigned to receive control or experimental treatment. HR hazard ratio, CI confidence interval, AI Adriamycin plus ifosfamide, GD gemcitabine plus docetaxel.

After the completion of patient accrual, the second interim analysis was performed on December 14, 2019 with a data cut-off on May 23, 2019. The median follow-up at that point was 2.3 years (IQR: 1.6–3.6 years), and death occurred in four patients in the AI group and 10 patients in the GD group. The median OS was not reached in either treatment group and the 3-year OSs were 91.4% in the AI group (95% CI: 78.1–96.7%) and 79.2% in the GD group (95% CI: 64.0–88.5%). Because the point estimate for HR exceeded the non-inferiority margin (HR: 2.55, 97.7% CI: 0.67–9.78, *p* = 0.78 for non-inferiority hypothesis) (95% CI: 0.80–8.14) (Fig. 2a), the JCOG DSMC recommended early study termination.

Although there were no significant differences in OS between the AI and GD groups according to histological grade and clinical stage, sensitivity analysis was performed to evaluate the influence of the imbalance regarding stage IIB and stage III disease. In the unstratified Cox regression analysis using clinical stage (IA to IIB for primary vs. III or IV for primary vs. recurrence) and treatment arm as covariates, the HR for poor OS was 2.08 (95% CI: 0.53–8.13), which also exceeded the non-inferiority margin.

Disease progression was observed for 14 patients in the AI group and 29 patients in the GD group. The median PFS was not reached in the AI group and was 4.4 years in the GD group (95% CI: 2.3 years–not reached). The 3-year PFS were 79.1% in the AI group (95% CI: 65.5–87.8%) and 59.1% in the GD group (95% CI: 45.3–70.5%). The likelihood of PFS was significantly poorer in the GD group (HR: 2.32, 95% CI: 1.22–4.39) (Fig. 2b). Detailed information was available for 42/43 patients who experienced progression. Local recurrence was noted for 3/70 patients (4.3%) in the AI group and for 6/73 patients (8.2%) in the GD group. Distant metastases were observed for 12 patients in the AI group and 23 patients in the GD group. The most frequent metastatic site was the lungs (nine patients in the AI group and 18 patients in the GD group).

Radiological response was assessed for 143 patients. In the AI group, the RECIST responses were partial response for one patient, stable disease for 59 patients, and progressive disease for six patients. In the GD group, the responses were partial response for three patients, stable disease for 54 patients, and progressive disease for 15 patients. No significant difference in the response rate was observed when we compared the AI group (1.4%, 95% CI: 0.04–7.7%) and the GD group (4.1%, 95% CI: 0.9–11.5%).

Surgical resection was performed in 129 patients (63 patients in the AI group and 66 patients in the GD group). Surgical margin was assessed as R0 for 56 patients in the AI group and 61 patients in the GD group, and as R1 for six patients in the AI group and three patients in the GD group. There was no significant difference in OS between both groups in terms of the histological margin status.

Histological response was assessed for 129 patients who underwent surgery. Complete response (Grade 4) was observed for two patients in the GD group, Grade 3 response was observed for six patients in each treatment arm, and Grade 2 response was observed for 15 patients in each treatment group. Grade 1 response was observed for 41 patients in the AI group and 42 patients in the GD group (Table 2). The histological response rates were 8.6% in the AI group (6/70 patients, 95% CI: 3.2–17.7%) and 11.0% in the GD group (8/73 patients, 95% CI: 4.9–20.5%), although this difference was not significant.

**Table 2:** Histological response to preoperative chemotherapy.

	Grade 1	Grade 2	Grade 3	Grade 4	NE	Proportion of histological response
AI (*n* = 63)	41	15	6	0	1	8.6% (6/70) (95% CI: 3.2–17.7%)
GD (*n* = 66)	42	15	6	2	1	11.0% (8/73) (95% CI: 4.9–20.5%)

Data are *n* unless noted otherwise.

*NE* not evaluable, *AI* Adriamycin plus ifosfamide, *GD* gemcitabine plus docetaxel, *CI* confidence interval.

Radiotherapy was administered as post-protocol treatment, when the surgical margin was considered insufficient at the discretion of the attending physician, to 19/143 patients (13.3%), including eight patients (7.3%) in the AI group and 11 patients (15.1%) in the GD group. There was no significant difference in OS between the AI and GD groups in terms of radiation therapy.

Furthermore, pre-planned exploratory subgroup analyses were also performed. There were no significant differences in OS between both treatment groups according to age, sex, performance status, tumour location, tumour size, histological subtype, histological grade, and clinical stage.

The haematological and non-haematological adverse events during the preoperative chemotherapy are shown in Table 3. No treatment-related deaths were observed. Among the 142 patients who underwent preoperative chemotherapy (69 patients in the AI group and 73 patients in the GD group), the most frequent Grade 3–4 adverse events were any haematological toxicities (64/69 patients [92.8%] in the AI group vs. 60/73 patients [82.2%] in the GD group), neutropenia (61/69 patients [88.4%] vs. 58/73 patients [79.5%]), leukopenia (60/69 patients [87.0%] vs. 50/73 patients [68.5%]), anaemia (34/69 patients [49.3%] vs. 4/73 patients [5.5%]), and thrombocytopenia (7/69 patients [10.1%] vs. 4/73 patients [5.5%]). Febrile neutropenia was also a frequent event (25/69 patients [36.2%] vs. 13/73 patients [17.8%]).

**Table 3:** Adverse events during preoperative chemotherapy.

	AI (*n* = 69)				
	Grade 1	Grade 2	Grade 3	Grade 4	%Grade 3–4
Haematological					
Anaemia	12	20	29	5	49.3
Leukopenia	0	2	10	50	87.0
Neutropenia	0	2	4	57	88.4
Thrombocytopenia	33	13	5	2	10.1
Febrile neutropenia	—	—	25	0	36.2
Any haematological	2	1	7	57	92.8
Non-haematological					
Aspartate aminotransferase increased	22	2	1	0	1.4
Alanine aminotransferase increased	30	3	3	0	4.3
Creatinine decreased	2	0	0	0	0
Fatigue	17	6	3	—	4.3
Oedema	5	0	0	—	0
Diarrhoea	3	0	1	0	1.4
Haematuria	5	0	1	0	1.4
Oral mucositis	9	3	3	0	4.3
Pneumonitis	1	0	0	0	0
Fever	18	7	0	0	0
Leukoencephalopathy	0	1	1	0	1.4
Palmar-plantar erythrodysesthesia	0	0	0	—	0
Skin hyperpigmentation	0	0	—	—	—
Vomiting	14	4	2	0	2.9
Anorexia	30	14	3	0	4.3
Blood bilirubin increased	4	1	0	0	0
Any infection	0	3	1	0	1.4
Any cardiac disorders	1	0	0	0	0
Any neural disorders	4	1	0	0	0
Any electrolyte abnormalities	39	1	4	0	5.8

	GD (*n* = 73)				
	Grade 1	Grade 2	Grade 3	Grade 4	%Grade 3–4
Haematological					
Anaemia	36	21	4	0	5.5
Leukopenia	2	9	44	6	68.5
Neutropenia	2	5	12	46	79.5
Thrombocytopenia	40	5	3	1	5.5
Febrile neutropenia	—	—	13	0	17.8
Any haematological	2	8	13	47	82.2
Non-haematological					
Aspartate aminotransferase increased	33	3	2	0	2.7
Alanine aminotransferase increased	26	11	7	0	9.6
Creatinine decreased	6	0	0	0	0
Fatigue	20	9	1	—	1.4
Oedema	3	1	0	—	0
Diarrhoea	6	3	1	0	1.4
Haematuria	2	0	0	0	0
Oral mucositis	4	2	1	0	1.4
Pneumonitis	2	2	2	0	2.7
Fever	21	5	0	0	0
Leukoencephalopathy	0	0	0	0	0
Palmar-plantar erythrodysesthesia	1	1	0	—	0
Skin hyperpigmentation	0	0	—	—	—
Vomiting	5	0	0	0	0
Anorexia	15	8	1	0	1.4
Blood bilirubin increased	7	1	0	0	0
Any infection	0	1	1	0	1.4
Any cardiac disorders	0	1	0	0	0
Any neural disorders	2	0	0	0	0
Any electrolyte abnormalities	24	1	2	0	2.7

Data are *n* unless noted otherwise.

*AI* Adriamycin plus ifosfamide, *GD* gemcitabine plus docetaxel.

Among the 98 patients who underwent postoperative chemotherapy (53 patients in the AI group and 45 patients in the GD group), the most frequent Grade 3–4 adverse events were also any haematological toxicities (45/53 patients [84.9%] in the AI group vs. 34/45 patients [75.6%] in the GD group), neutropenia (43/53 patients [81.1%] vs. 33/45 patients [73.3%]), leukopenia (45/53 patients [84.9%] vs. 30/45 patients [66.7%]), anaemia (7/53 patients [13.2%] vs. 0/45 patients [0%]), and thrombocytopenia (23/53 patients [43.4%] vs. 2/45 patients [4.4%]). Febrile neutropenia was less common during postoperative chemotherapy (7/53 patients [13.2%] vs. 2/45 patients [4.4%]) (Table 4).

**Table 4:** Adverse events during postoperative chemotherapy.

	AI (*n* = 53)				
	Grade 1	Grade 2	Grade 3	Grade 4	%Grade 3–4
Haematological					
Anaemia	14	27	7	0	13.2
Leukopenia	0	4	5	40	84.9
Neutropenia	3	3	1	42	81.1
Thrombocytopenia	16	10	16	7	43.4
Febrile neutropenia	—	—	7	0	13.2
Any haematological	1	5	3	42	84.9
Non-haematological					
Aspartate aminotransferase increased	13	1	0	0	0
Alanine aminotransferase increased	16	2	1	0	1.9
Creatinine decreased	3	0	0	0	0
Fatigue	11	7	0	—	0
Oedema	1	1	0	—	0
Diarrhoea	0	0	1	0	1.9
Haematuria	2	0	0	0	0
Oral mucositis	1	0	1	0	1.9
Pneumonitis	0	0	0	0	0
Fever	8	3	1	0	1.9
Leukoencephalopathy	0	0	0	0	0
Palmar-plantar erythrodysesthesia	0	0	0	—	0
Skin hyperpigmentation	1	0	—	—	—
Vomiting	4	0	0	0	0
Anorexia	14	10	0	0	0
Blood bilirubin increased	0	0	0	0	0
Any infection	0	0	3	0	5.7
Any cardiac disorders	0	0	0	0	0
Any neural disorders	2	1	0	0	0
Any electrolyte abnormalities	23	1	3	0	5.7

	GD (*n* = 45)				
	Grade 1	Grade 2	Grade 3	Grade 4	%Grade 3–4
Haematological					
Anaemia	23	13	0	0	0
Leukopenia	3	3	27	3	66.7
Neutropenia	2	3	7	26	73.3
Thrombocytopenia	21	7	2	0	4.4
Febrile neutropenia	—	—	2	0	4.4
Any haematological	3	4	8	26	75.6
Non-haematological					
Aspartate aminotransferase increased	15	2	0	0	0
Alanine aminotransferase increased	14	6	0	0	0
Creatinine decreased	1	0	0	0	0
Fatigue	11	1	0	—	0
Oedema	4	1	0	—	0
Diarrhoea	1	0	0	0	0
Haematuria	0	0	0	0	0
Oral mucositis	2	0	0	0	0
Pneumonitis	0	0	0	0	0
Fever	12	0	0	0	0
Leukoencephalopathy	0	0	0	0	0
Palmar-plantar erythrodysesthesia	0	1	0	—	0
Skin hyperpigmentation	0	0	—	—	—
Vomiting	1	0	0	0	0
Anorexia	5	3	0	0	0
Blood bilirubin increased	1	0	0	0	0
Any infection	0	1	0	0	0
Any cardiac disorders	0	0	0	0	0
Any neural disorders	0	0	0	0	0
Any electrolyte abnormalities	12	0	2	0	4.4

Data are *n* unless noted otherwise.

*AI* Adriamycin plus ifosfamide, *GD* gemcitabine plus docetaxel.

## Discussion

The phase II/III trial, JCOG1306 aimed to confirm whether GD chemotherapy was non-inferior to the standard AI chemotherapy for patients with high-risk STS in the extremities or trunk. The second interim analysis revealed that the HR in the GD group exceeded the predetermined non-inferiority margin (HR > 1.61). Furthermore, the PFS in the GD group was significantly inferior to that in the AI group. Based on the methods of Spiegelhalter et al. [22], the predictive probability that the final analysis would reveal non-inferiority in the GD group was as low as 11.5%, and there was a 71.5% chance that the HR in the GD group would exceed the non-inferiority margin. Therefore, based on the recommendation from the JCOG DSMC, we decided to terminate the study early and open the results.

To date, the ISG has shown that the addition of adjuvant chemotherapy to surgery provides superior OS for patients with STS [6]. The ISG also demonstrated that three preoperative courses of EI was non-inferior to five perioperative courses of EI [9]. Moreover, the ISG-STS1001 trial revealed that preoperative EI provided significantly better OS than histotype-tailored chemotherapy [23]. The results from our AI group are comparable to those from the ISG RCTs, which appears to confirm the efficacy of perioperative chemotherapy using anthracycline plus IFM. While ISG-STS1001 and JCOG1306 trials did not directly compare perioperative chemotherapy and surgery, the results may suggest that perioperative chemotherapy using a full-dose AI regimen might provide a survival benefit for high-risk STS patients.

EORTC62931 enrolled patients with localised STS with no restrictions according to site, depth, or size, but it failed to confirm the superiority of adjuvant chemotherapy [7], which suggests that the target for adjuvant chemotherapy of STS should be limited. The three ISG RCTs [6, 9, 23] and the present study used nearly identical inclusion criteria, which were high-grade deeply located STS tumours that were >5 cm in the extremities and trunk. Therefore, patients with these types of high-risk STS might benefit from perioperative chemotherapy.

A new nomogram (the “Sarculator”) might also be useful for identifying patients with high-risk STS who might benefit from perioperative chemotherapy [24]. When high-risk STS cases from the EORTC62931 trial were screened using the Sarculator, subgroup analysis revealed that adjuvant chemotherapy was associated with significant benefits in terms of OS and DFS [25]. Furthermore, the CINSARC signature has also been proposed for predicting the prognosis of cancer patients based on their tumour’s gene expression profile [26], and an ongoing RCT is evaluating 3 vs. 6 courses of AI chemotherapy for high-risk STS that is identified based on the CINSARC signature [27]. Our findings also solidify the status of the standard AI regimen as the preferred perioperative chemotherapy for high-risk STS. Nevertheless, further studies may be needed to refine the definition of “high-risk STS” and clarify which patients will experience the greatest survival benefit from perioperative chemotherapy.

Most evidence regarding GD chemotherapy has been based on studies of advanced STS, and we are only aware of two trials that have evaluated the GD regimen as perioperative chemotherapy for STS. ISG-STS1001 evaluated the GD regimen as one of five experimental treatments for 97 patients with UPS. The GD regimen from that study (GEM at 900 mg/m^2^ and DOC at 75 mg/m^2^) was nearly identical to the regimen used in our study. The subgroup analysis of UPS in ISG-STS1001 revealed that there were no significant differences in OS and DFS between the standard EI and GD groups [23]. A randomised phase II trial (UMMC-2004.010) also compared the AI and GD regimens for localised high-risk STS, which revealed a significantly better 4-year DFS in the GD group (69 vs. 50%, *p* = 0.032) and a non-significantly better 4-year OS in the GD group (73 vs. 68%, *p* = 0.928) [14]. The dose of DOC in the UMMC-2004.010 trial (100 mg/m^2^) was noticeably higher than the dose in our trial (70 mg/m^2^), which was determined based on the results from a feasibility study that evaluated Japanese patients with STS [28]. Many previous trials involving the GD regimen used the higher DOC dose (100 mg/m^2^) [13], although a recent phase III study (the GeDDiS trial) evaluated patients with advanced STS who received ADM alone or the GD regimen with a lower dose of DOC (GEM at 675 mg/m^2^ and DOC at 75 mg/m^2^) [29]. That dose was selected because Maki et al. found that 46% of their patients required a dose reduction from GEM at 900 mg/m^2^ and DOC at 100 mg/m^2^, and that ≥40% of the patients who received a reduced dose subsequently stopped GD treatment because of haematological toxicities [13]. Thus, a reduced-dose GD regimen was used in the GeDDiS trial, although the results in that GD group were similar to those reported by Maki et al. [25]. Similar to these results for advanced STS, comparable OS was observed after GD treatment for localised STS in UMMC-2004.010 and JCOG1306 (4-year OS: 73 vs. 73.5%).

Interestingly, the efficacy in the AI group of UMMC-2004.010 was noticeably lower than in our study (4-year OS: 68 vs. 91.3%). Furthermore, our previous trial (JCOG0304), which evaluated very similar subjects, revealed a 4-year OS of 82.6% in the AI group [10]. Moreover, standard EI treatment in ISG-STS1001 provided an OS rate of 76% at 60 months [23]. Thus, the results in our AI group were comparable to those in previous reports. Nevertheless, the IFM doses were 7.5 g/m^2^ in the UMMC-2004.010 trial, 9 g/m^2^ in the ISG studies, and 10 g/m^2^ in the JCOG studies. These differences in the IFM dose may have affected the outcomes in the AI groups from the different studies.

Other differences between our study and the UMMC-2004.010 trial were that the tumour location was not limited to the extremities and trunk, patients received four chemotherapy courses, treatment was administered as postoperative chemotherapy for approximately 30% of the patients, and radiotherapy was performed for most patients (89%). Furthermore, fixed-dose rate administration of GEM, which was used in most previous studies including UMMC-2004.010, was not used in JCOG1306 since the method was not allowed by insurance in Japan. It is unclear what factors influence the differences in the results between our study and UMMC-2004.010, and a comparative analysis is needed using participant-level data.

In the present study, histological response was evaluated based on tumour necrosis in the resected specimen. However, there are no validated criteria for evaluating histological response in STS, and the prognostic significance of histological response to chemotherapy remains controversial. Recently, we attempted to establish a standardised evaluation method of histological response to preoperative chemotherapy with high agreement scores among pathologists using specimens from the patients who are registered in JCOG0304 [10]. In the ancillary study, JCOG0304-A1, the tumour cells displaying cellular swelling, nuclear swelling, increased eosinophilia of cytoplasm, or slight vacuolation were defined as viable. Those displaying any evidence of pyknosis, karyorrhexis, karyolysis, severe vacuolation, or loss of nuclear staining were defined as non-viable. The results demonstrated a substantial agreement in the weighted κ score of 0.71 among six pathologists who specialise in STS [30]. We are currently carrying out a validation study of this method using the resected specimens from JCOG1306. Further investigation is needed to establish a consensus in the evaluation method of histological response to preoperative chemotherapy in STS.

This study had several limitations. First, there were imbalances in the histological subtypes, as UPS and leiomyosarcoma were more common in the GD group, while liposarcoma, especially myxoid subtype, was more common in the AI group. When the pre-planned exploratory subgroup analyses were performed, no significant difference in OS was observed between the treatment groups according to the histological subtype. As GD is considered highly effective for leiomyosarcoma [31], the higher number of patients with leiomyosarcoma in the GD group might have helped favour the GD outcomes, although it is also possible that the differences between the treatment arms were also influenced by the imbalanced histological subtypes. Second, there were imbalances in the histological grades, as the GD group had a larger number of patients with Grade 3 tumours (36 patients vs. 22 patients) and a larger number of patients with stage III disease (36 patients vs. 24 patients). There were no significant differences in OS between the AI and GD groups according to histological grade and clinical stage. Moreover, when the sensitivity analysis was performed to evaluate whether these imbalances influenced survival, the conclusion was not changed after Cox regression analysis (not stratified according to stage and treatment arm), as the HR of 2.08 still exceeded the non-inferiority margin (HR: 1.61). However, it is noteworthy that Grade 3 tumours were associated with a greater HR (2.641) than Grade 2 tumours (HR: 1.379), which suggested that AI chemotherapy might be more beneficial for Grade 3 tumours. Third, the results were based on the second interim analysis with a median follow-up period of only 2.4 years, which highlights the need for observation until the final analysis. Fourth, the rarity of STS limited the sample size to 143 patients, which may limit the power of the analyses.

In conclusion, although the GD regimen had milder toxicity than the AI regimen, its non-inferiority could not be confirmed. Therefore, it appears that the GD regimen, as administered in JCOG1306, should not be considered a standard treatment of perioperative chemotherapy for high-risk STS in the extremities and trunk.

## Supplementary information

Supplemental Appendix

CONSORT Checklist

## Data Availability

The data sets generated and/or analysed during the current study are available from the corresponding author on reasonable request.

## References

[1] WHO Classification of Tumours Editorial Board. WHO classification of tumours, soft tissue and bone tumours. Lyon: International Agency for Research on Cancer; 2020.

[2] American Cancer Society. Cancer facts and figures 2022, soft tissue sarcoma. https://www.cancer.org/cancer/soft-tissue-sarcoma.html. 2022. Accessed 28 Apr 2022.

[3] Japanese Orthopaedic Association Musculoskeletal Tumor Committee. Soft Tissue Tumor Registry in Japan 2019. Tokyo: National Cancer Centre; 2019.

[4] National Comprehensive Cancer Network. Clinical Practice Guidelines in Oncology. Soft tissue sarcoma, version 1. https://www.nccn.org/professionals/physician_gls/pdf/sarcoma.pdf. 2022. Accessed 28 Apr 2022.

[5] Gronchi A, Miah AB, Dei Tos AP, Abecassis N, Bajpai J, Bauer S (2021). Soft tissue and visceral sarcomas: ESMO-EURACAN-GENTURIS clinical practice guidelines for diagnosis, treatment and follow-up. Ann Oncol..

[6] Frustaci S, Gherlinzoni F, De Paoli A, Bonetti M, Azzarelli A, Comandone A (2001). Adjuvant chemotherapy for adult soft tissue sarcomas of the extremities and girdles: results of the Italian randomized cooperative trial. J Clin Oncol..

[7] Woll PJ, Reichardt P, Le Cesne A, Bonvalot S, Azzarelli A, Hoekstra HJ (2012). Adjuvant chemotherapy with doxorubicin, ifosfamide, and lenograstim for resected soft-tissue sarcoma (EORTC 62931): a multicentre randomised controlled trial. Lancet Oncol..

[8] Pervaiz N, Colterjohn N, Farrokhyar F, Tozer R, Figueredo A, Ghert M (2008). A systematic meta-analysis of randomized controlled trials for adjuvant chemotherapy for localized resectable soft tissue sarcoma. Cancer.

[9] Gronchi A, Frustaci S, Mercuri M, Martin J, Lopez-Pousa A, Verderio P (2012). Short, full-dose adjuvant chemotherapy in high-risk adult soft tissue sarcomas: a randomized clinical trial from the Italian Sarcoma Group and the Spanish Sarcoma Group. J Clin Oncol..

[10] Tanaka K, Mizusawa J, Fukuda H, Araki N, Chuman H, Takahashi M (2015). Perioperative chemotherapy with ifosfamide and doxorubicin for high-grade soft tissue sarcomas in the extremities (JCOG0304). Jpn J Clin Oncol..

[11] Tanaka K, Mizusawa J, Naka N, Kawai A, Katagiri H, Hiruma T (2019). Ten-year follow-up results of perioperative chemotherapy with doxorubicin and ifosfamide for high-grade soft-tissue sarcoma of the extremities: Japan Clinical Oncology Group study JCOG0304. BMC Cancer.

[12] Gronchi A, Stacchiotti S, Verderio P, Ferrari S, Martin Broto J, Lopez-Pousa A (2016). Short, full-dose adjuvant chemotherapy in high-risk adult soft tissue sarcomas: long-term follow-up of a randomized clinical trial from the Italian sarcoma group and the Spanish sarcoma group. Ann Oncol..

[13] Maki RG, Wathen JK, Patel SR, Priebat DA, Okuno SH, Samuels (2007). Randomized phase II study of gemcitabine and docetaxel compared with gemcitabine alone in patients with metastatic soft tissue sarcomas: results of sarcoma alliance for research through collaboration study 002. J Clin Oncol..

[14] Davis EJ, Chugh R, Zhao L, Lucas DR, Biermann JS, Zalupski MM (2015). A randomised, open-label, phase II study of neo/adjuvant doxorubicin and ifosfamide versus gemcitabine and docetaxel in patients with localised, high-risk, soft tissue sarcoma. Eur J Cancer.

[15] Kataoka K, Tanaka K, Mizusawa J, Kimura A, Hiraga H, Kawai A (2014). A randomized phase II/III trial of perioperative chemotherapy with adriamycin plus ifosfamide versus gemcitabine plus docetaxel for high-grade soft tissue sarcoma: Japan Clinical Oncology Group Study JCOG1306. Jpn J Clin Oncol.

[16] Fletcher, CDM, Bridge, JA, Hogendoorn, PCW, Mertens, F. WHO classification of tumours of soft tissue and bone. Lyon: International Agency for Research on Cancer; 2013.

[17] Guillou L, Coindre JM, Bonichon F, Nguyen BB, Terrier P, Collin F (1997). Comparative study of the National Cancer Institute and French Federation of Cancer Centers Sarcoma Group grading systems in a population of 410 adult patients with soft tissue sarcoma. J Clin Oncol..

[18] Edge, SB, Byrd, DR, Compton, CC, Fritz AG, Greene FL, Trotti A, editors. AJCC cancer staging manual, 7th edition. Cham: Springer; 2011.

[19] Eisenhauer EA, Therasse P, Bogaerts J, Schwartz LH, Sargent D, Ford R (2009). New response evaluation criteria in solid tumours: revised RECIST guideline (version 1.1). Eur J Cancer.

[20] Schoenfeld DA, Richter JR (1982). Nomograms for calculating the number of patients needed for a clinical trial with survival as an endpoint. Biometrics.

[21] Lan KKG, DeMets DL (1983). Discrete sequential boundaries for clinical trials. Biometrika.

[22] Spiegelhalter DJ, Freedman LS, Parmar MK (1993). Applying Bayesian ideas in drug development and clinical trials. Stat Med..

[23] Gronchi A, Palmerini E, Quagliuolo V, Martin Broto J, Lopez Pousa A, Grignani G (2020). Neoadjuvant chemotherapy in high-risk soft tissue sarcomas: final results of a randomized trial from Italian (ISG), Spanish (GEIS), French (FSG), and Polish (PSG) Sarcoma Groups. J Clin Oncol..

[24] Callegaro D, Miceli R, Bonvalot S, Ferguson P, Strauss DC, Levy A (2016). Development and external validation of two nomograms to predict overall survival and occurrence of distant metastases in adults after surgical resection of localised soft-tissue sarcomas of the extremities: a retrospective analysis. Lancet Oncol.

[25] Pasquali S, Pizzamiglio S, Touati N, Litiere S, Marreaud S, Kasper B (2019). The impact of chemotherapy on survival of patients with extremity and trunk wall soft tissue sarcoma: revisiting the results of the EORTC-STBSG 62931 randomised trial. Eur J Cancer..

[26] Chibon F, Lagarde P, Salas S, Pérot G, Brouste V, Tirode F (2010). Validated prediction of clinical outcome in sarcomas and multiple types of cancer on the basis of a gene expression signature related to genome complexity. Nat Med..

[27] Italiano A, Blay JY, Le Cesne A, Bompas E, Piperno-Neumann S, Duffaud F (2019). Benefit of intensified perioperative chemotherapy within high-risk CINSARC patients with resectable soft tissue sarcomas (CIRSARC). J Clin Oncol..

[28] Takano T, Niikura H, Ito K, Nagase S, Utsunomiya H, Otsuki T (2014). Feasibility study of gemcitabine plus docetaxel in advanced or recurrent uterine leiomyosarcoma and undifferentiated endometrial sarcoma in Japan. Int J Clin Oncol..

[29] Seddon B, Strauss SJ, Whelan J, Leahy M, Woll PJ, Cowie F (2017). Gemcitabine and docetaxel versus doxorubicin as first-line treatment in previously untreated advanced unresectable or metastatic soft-tissue sarcomas (GeDDiS): a randomised controlled phase 3 trial. Lancet Oncol.

[30] Oda Y, Tanaka K, Hirose T, Hasegawa T, Hiruta N, Hisaoka M (2022). Standardization of evaluation method and prognostic significance of histological response to preoperative chemotherapy in high-grade non-round cell soft tissue sarcomas. BMC Cancer.

[31] Hensley ML, Miller A, O’Malley DM, Mannel RS, Behbakht K, Bakkum-Gamez JN (2015). Randomized phase III trial of gemcitabine plus docetaxel plus bevacizumab or placebo as first-line treatment for metastatic uterine leiomyosarcoma: an NRG Oncology/Gynecologic Oncology Group study. J Clin Oncol..

