# Supplementary material for: Perioperative Adriamycin plus ifosfamide vs. gemcitabine plus docetaxel for high-risk soft tissue sarcomas: randomised, phase II/III study JCOG1306
Source: Br J Cancer. 2022 Jul 23;127(8):1487–96. doi: 10.1038/s41416-022-01912-5 (PMC9553903; doi:10.1038/s41416-022-01912-5)
Supplement: Supplementary file 1 — Supplemental Appendix [file 41416_2022_1912_MOESM1_ESM.docx]

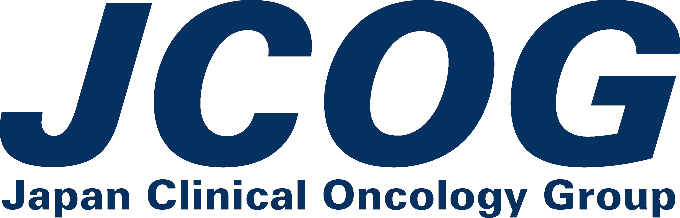


**Japan Clinical Oncology Group**

**Bone and Soft Tissue Tumor Study Group**

Practical Research for Innovative Cancer Control from Japan Agency for Medical Research and Development

"Establishment of the new standard treatments for patients with high-grade bone and soft tissue sarcomas"

National Cancer Center Research and Development Fund 29-A-3

"Scientific research on multi-institutional trials to establish new standard treatment of solid tumors in adults"

**JCOG1306**

**Randomized phase II/III study of adjuvant chemotherapy with Adriamycin + Ifosfamide vs Gemcitabine + Docetaxel for high-grade soft tissue sarcomas**

**Study Protocol ver.1.4.0**

**AI vs GD for STS RPII/III**

**Group Chair: Toshifumi OZAKI**

Department of Orthopaedic Surgery, Okayama University Hospital

**Study Chair (Principal Investigator): Yukihide IWAMOTO**

Kyushu Rosai Hospital

1-1 Sone-Kita, Kokura-Minami-ku, Kitakyushu City, Fukuoka 800-0296, Japan

TEL: +81-93-471-1121

FAX: +81-93-475-5545

E-Mail:yiwamoto@ortho.med.kyushu-u.ac.jp

**Study Coordinator: Kazuhiro TANAKA**

Department of Orthopedics, Oita University Hospital

1-1 Idaigaoka, Hasama, Yufu City, Oita 879-5593, Japan

TEL: +81-97-586-5872

FAX: +81-97-586-6647

E-mail: ktanaka@oita-u.ac.jp

June 29, 2013 Protocol concept approved by JCOG Executive Committee (PC1306)

May 13, 2019 Revision v1.3. approved by JCOG Data and Safety Monitoring Committee

May 17, 2019 Approved by Certified Review Board of National Cancer Center Hospital

Nov 1, 2019 Revision v1.4.0 approved by JCOG Data and Safety Monitoring Committee

Dec 19, 2019 Approved by Certified Review Board of National Cancer Center Hospital

# Summary

This study is conducted as a "specified clinical trial" based on the Clinical Research Act (Act No. 16 of 2017).

In this protocol, the Principal Investigator refers to Study Chair in JCOG.

Study name: Randomized phase II/III study of adjuvant chemotherapy with Adriamycin+Ifosfamide vs Gemcitabine+Docetaxel for high-grade soft tissue sarcoma.

Simple study name: "Randomized Phase II/III Study of Adjuvant Chemistry for High-grade Soft Tissue Sarcoma"

## Schema


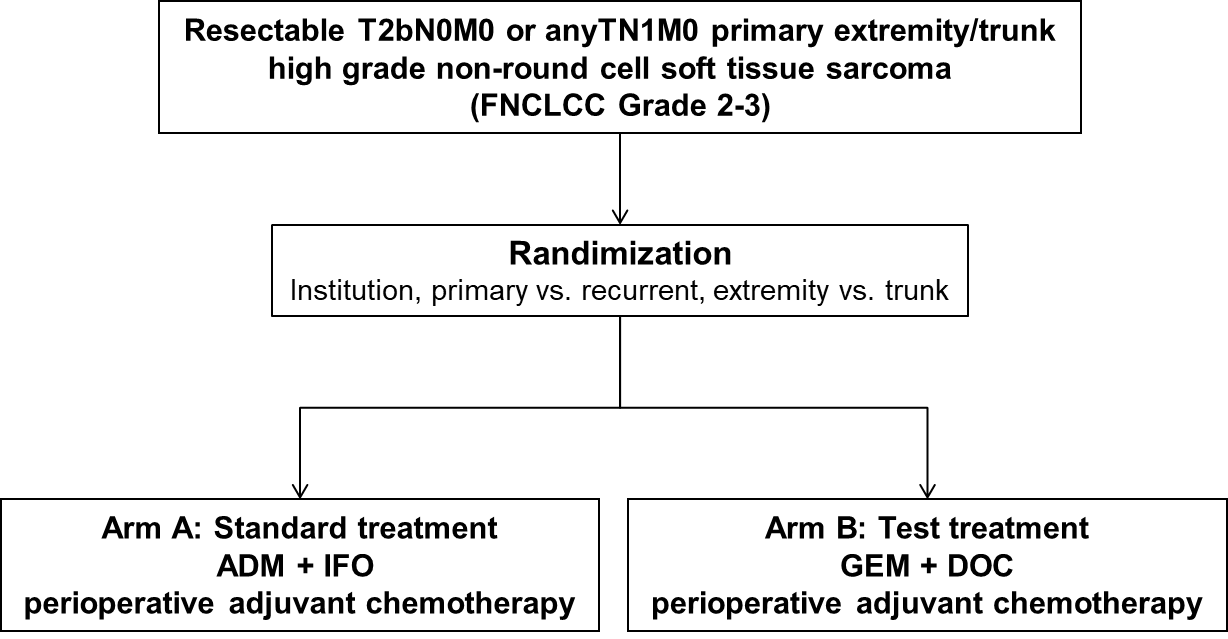


## Objectives

To evaluate the efficacy and safety of perioperative chemotherapy with Gemcitabine (GEM) plus Docetaxel (DOC) and Adriamycin (ADM) plus Ifosfamide (IFO) in patients with resectable T2bN0M0 or anyTN1M0 primary extremity/trunk high grade non-round cell soft tissue sarcoma (histologic grade Grade2-3 by FNCLCC system, including patients with local recurrence without history of upfront chemotherapy and radiotherapy), and to confirm the non-inferiority of perioperative chemotherapy with GEM plus DOC to that with ADM plus IFO in terms of overall survival.

**Phase II part**

Primary endpoint: proportion of completion of pre-operative chemotherapy without progressive disease

(Proportion of patients who complete 3 courses of pre-operative chemotherapy and whose response is CR, PR or SD among all registered patients)

Secondary endpoints: progression-free survival, response rate of preoperative chemotherapy, pathological response rate,

proportion of preservation of diseased limb (extremities), disease control rate (extremities and trunks), proportion of adverse events

proportion of serious adverse events, proportion of respiratory-related adverse events, and proportion of post-operative complications

**Phase III part**

Primary endpoint: overall survival

Secondary endpoints: progression-free survival, response rate to neoadjuvant chemotherapy, pathological response rate,

proportion of preservation of diseased limb (extremities), disease control rate (extremities and trunks), proportion of adverse events

proportion of serious adverse events, proportion of respiratory-related adverse events, and proportion of post-operative complications

## Subjects:

※ For patient registration, refer to "4.2. Exclusion criteria.

1. HistologicalIy proven non-round cell sarcoma^※^.

Any of the following histologic types (WHO-classification 2013 edition) and histological grade by FNCLCC system are Grade 2-3.

High-grade undifferentiated pleomorphic sarcoma (malignant fibrous histiocytoma: according to the 2002 edition of the WHO classification), fibrosarcoma, myxofibrosarcoma, leiomyosarcoma (conventional, poorly differentiated/pleomorphic/epithelioid), synovial sarcoma, liposarcoma (myxoid, high-grade myxoid, dedifferentiated, pleomorphic), pleomorphic rhabdomyosarcoma, malignant peripheral nerve sheath tumor (conventional, poorly differentiated), angiosarcoma (conventional, poorly differentiated/epithelioid), undifferentiated sarcoma/unclassifid sarcoma

- Histopathologically confirmed: In the primary case, definitive diagnosis of histologic type by incisional biopsy is mandatory. Local recurrence can be diagnosed by needle biopsy in addition to incisional biopsy at the time of initial onset.

1. Primary tumor or first local recurrent^※^ tumor

- Local recurrent tumor refers to the tumor of the same histology occurred in or around the site where the primary tumor was resected at the initial tumor resection

1. Ten or more unstained tumor tissue slides available for central review, whose biopsy specimen was used for the diagnosis described in 1) (in cases of local recurrence, submission of the pathological specimen at the initial diagnosis is mandatory, but he/she is eligible even if 10 unstained specimens are not available).
2. In the case of primary tumor, the patient has been diagnosed as T2bN0M0 or anyTN1M0 (UICC/AJCC 7th edition) by most recent imaging (chest CT, regional MRI: plain CT/MRI is acceptable) within 28 days prior to registration.
3. The largest diameter of the tumor should be the longest, whether transverse, coronal, or sagittal. However, in the case of N1, the primary lesion is of any size and depth.
4. N1 is defined as enlarged lymph nodes ≧10 mm in short diameter on CT or MRI of the regional lymph node area (see 3.3).
5. In case of primary local recurrence, there is no apparent distant metastasis by the most recent imaging examination (chest CT) within 28 days before registration. No limitation for tumor size and depth of primary tumor and recurrent tumor.
6. Tumor is in the extremities or trunk.

Extremities (upper limbs): shoulder, axilla, upper arm, elbow, forearm, wrist, or hand

Extremities (lower limbs: gluteal, inguinal, thigh, knee, lower leg, ankle, or foot

Trunk: the surface of the chest wall, abdominal wall, lumbar region, or back without tumor invasion to the thoracic cavity, abdominal cavity, or spinal canal.

1. Having measurable lesion^※^ on MRI axial section.

- Non-nodal lesion with a maximum diameter of 10 mm or greater on MRI with a slice thickness of 5 mm or less or nodal lesion with a short diameter of 15 mm or greater on MRI or CT with a slice thickness of 5 mm or less

1. Marginal resection or wide margin resection is considered feasible (see 3.4).
2. Age of the registration date is 20 years or older and 70 years or younger.
3. Performance status (PS) is 0 or 1 according to ECOG criteria.
4. There is no history of chemotherapy or radiation therapy, including treatment of the other cancer.
5. The most recent laboratory test within 14 days prior to registration (the same day of the week 2 weeks prior to the registration date is acceptable) meets all of the following:
   1. Neutrophil count ≧1,500/mm^3^
   2. Hemoglobin≧8.0 g/dL (no blood transfusions within 14 days prior to registration)
   3. Platelet count ≧10×10^4^ / mm^3^
   4. Total bilirubin ≦ 1.5 mg/dL
   5. AST(GOT) ≦ 100 IU/L
   6. ALT(GPT) ≦ 100 IU/L
   7. Creatinine ≦ 1.5 mg/dL
   8. Creatinine clearance^*^ ≧ 60 mL/min (estimated)

(Cockcroft-Gault's Ccr formula)

Male: {(140-Age) × Body weight (kg)}/{72 × Serum creatinine level (mg/dL)}

Females: 0.85×{(140-Age)×Body weight (kg)}/{72×Serum creatinine level (mg/dL)}

* If the estimated value by Cockcroft-Gault formula is less than 60 mL/min, he/she is eligible if the creatinine clearance calculated using 24-hour urine collection is 60 mL/min or more.

1. Normal or no change requiring treatment on the most recent 12-lead resting ECG within 28 days before registration (the same day of the week 4 weeks before registration is allowed).
2. Neither interstitial pneumonitis, pulmonary fibrosis, nor severe emphysema as diagnosed by chest CT is complicated.
3. Written informed consent was obtained.

## Treatment

Preoperative chemotherapy (3 courses) plus tumor resection plus postoperative chemotherapy (2 courses).

Arm A: Chemotherapy regimens (common to pre- and post-operative chemotherapy)

| Drug | Dosage | Dosing regimen (dosing time) | Dose day |
| --- | --- | --- | --- |
| Adriamycin  (doxorubicin) | 30 mg/m^2^ | Div (2 h) | Day1,2 |
| Ifosfamide | 2 g/m^2^ | Div (4 h) | Day1-5 |

Arm B: Chemotherapy regimens (common to pre- and post-operative chemotherapy)

| Drug | Dosage | Dosing regimen (dosing time) | Dose day |
| --- | --- | --- | --- |
| Gemcitabine | 900 mg/m^2^ | Div (30 min) | Day 1, 8 |
| Docetaxel | 70 mg/m^2^ | Div (1 h) | Day 8 |

## Planned sample size and duration of the study

Planned sample size: 140 patients

Planned accrual period: 6 years.

Follow-up period: 5 years after completion of accrual.

Analysis period: 1 year

Total study duration :12 years

Primary analysis is performed 3 years after completion of accrual

Start date of the study February 17, 2014

Expected completion date of the study Feb 17, 2026

## Contact information

Eligibility criteria, treatment modification criteria, etc. that require clinical judgment: Study Coordinator (cover, 16.6.)

Enrollment procedures, CRFs completed, etc. JCOG Data Centre (16.14.).

Adverse Event Reporting, etc.; JCOG Data and Safety Monitoring Committee Office (16.11.)

# Criteria and Definitions Used in This Study

## Histological classification and histological grading

Histological Grading Sysytem by the French Federation of Cancer Center (FNCLCC) is used in this study.

Tumor differentiation score by histological type in Table 3.1.1., and the degree of necrosis and the sum of the number of mitoses in 10 fields at 400× higher magnification (/10 high-power fields, HPFs) in Table 3.1.2. are used, then, histological grade is classified as either Grade 1, Grade 2, Grade 3 according to the sum of scores in Tables 3.1.2.

When counting mitoses, use properly fixed and prepared specimens, and recognize mitotic divisions accurately, and select the site with the highest number of mitoses in the area of high cellularity.

### Tumor Differentiation Score for Soft Tissue Sarcomas.

Table 3.1.1. Tumor differentiation scores of soft tissue sarcomas according to FNCLCC system (only histological types as subjects in this study was excerpted)

| Histological type (according to 2013 WHO classification) | Tumor differentiation score |
| --- | --- |
| Myxoid liposarcoma | 2 |
| High-grade myxoid liposarcoma | 3 |
| Dedifferentiated liposarcoma | 3 |
| Pleomorphic liposarcoma | 3 |
| Fibrosarcoma (Adult/conventional fibrosarcoma) | 2 |
| Myxofibrosarcoma | 2 |
| High-grade undifferentiated pleomorphic sarcoma  (Malignant fibrous histiocytoma (2002 WHO classification)) | 3 |
| Conventional leiomyosarcoma | 2 |
| Poorly differentiated/pleomorphic/epithelioid leiomyosarcoma | 3 |
| Synovial sarcoma | 3 |
| Pleomorphic rhabdomyosarcoma | 3 |
| Conventional malignant peripheral nerve sheath tumor (MPNST) | 2 |
| Poorly differentiated malignant peripheral nerve sheath tumor (Poorly differentiated MPNST). | 3 |
| Conventional angiosarcoma | 2 |
| Poorly differentiated/epithelioid angiosarcoma | 3 |
| Undifferentiated sarcoma*/unclassifiid sarcoma ** | 3 |

* Excluding pleomorphic type.

** Non-round cell sarcoma but with undetermined histological type that is likely to be any of the above histological type.

Table 3.1.2. Histological grade of soft tissues by FNCLCC system

| Parameter |  | Evaluation criteria |
| --- | --- | --- |
| I Tumor differentiation score | Score 1 | According to Table 3.1.1 |
|  | Score 2 | According to Table 3.1.1 |
|  | Score 3 | According to Table 3.1.1 |
| II Degree of necrosis | Score 0 | No necrosis seen in any of the sections |
|  | Score 1 | Necrotic area < 50% |
|  | Score 2 | Necrotic area ≧ 50% |
| III Number of mitoses | Score 1 | 0-9/10 high-power fields (HPF) (400×) |
|  | Score 2 | 10-19/10HPF |
|  | Score 3 | ≧ 20/10 HPF |

| Histological Grade | (two-stage classification by Hajdu system) |  |
| --- | --- | --- |
| Grade 1 | Low grade | Total of I, II, III: 2-3 points |
| Grade 2 | High grade | Total of I, II, III: 4-5 points |
| Grade 3 | High grade | Total of I, II, III: 6-8 points |

## Stage classification (UICC/AJCC-TMN 7th edition)

T – Primary Tumor

TX: Primary tumor cannot be assessed

T0: No evidence of primary tumor

T1: Tumor 5 cm or less in greatest dimension

T1a: Superficial tumor*

T1b: Deep tumor*

T2: Tumor more than 5 cm in greatest dimension

T2a: Superficial tumor*

T2b: Deep tumor*

* Superficial tumor is a tumor confined superficially than superficial fascia and do not invade the superficial fascia.

Deep tumor are a tumor confined deeper than the superficial fascia or invaded or penetrated the superficial fascia

Sarcomas in the retroperitoneum, mediastinal, and pelvis are classified as deep tumors

N - Regional Lymph Nodes

NX: Regional lymph node cannot be assessed

N0: No regional lymph node metastases

N1: Regional lymph node metastasis

M - Distant Metastasis

M0: No distant metastasis

M1: Distant metastasis

In this study, the following cases are treated as M0 instead of distant metastasis.

① If multiple non-continuous lesions are present, but all are only within the same muscle compartment.

② When multiple non-continuous lesions (one or more non-continuous lesions in one muscle compartment) are present within multiple muscle compartments, but all muscle compartments where lesions are present are in contact with each other.

Table 3.2.1 Definitions of Boundary Zone Between Sites (Adapted from UICC TNM 7th Edition Skin Tumors)

| Section | Along |
| --- | --- |
| Right/left | Midline |
| Head and neck/thorax, back | Lower border of 7th cervical vertebra |
| Thorax, back/upper limb | Deltoid pectoralis major groove-axilla-scapula |
| Thoraxl/abdomen | Arcus costalis |
| Back/loins | Lower border of 12th thoracic vertebra |
| Loins/buttocks | Inferior border of 5th lumbar vertebra |
| Abdomen, buttocks/lower limb | Inguinal ligament-upper border of greater trochanter-gluteal fissure |

Table 3.2.2 Stage classification (Subjects of this study are bold and shaded)

| Stage | Tumor size and depth | Lymph node metastasis | Distant metastasis | Histological Grade |
| --- | --- | --- | --- | --- |
| IA | T1a, T1b | N0 | M0 | Grade 1 |
| IB | T2a, T2b | N0 | M0 | Grade 1 |
| IIA | T1a, T1b | N0 | M0 | Grade 2, 3 |
| IIB | T2a | N0 | M0 | Grade 2 |
|  | **T2b** | **N0** | **M0** | **Grade 2** |
| III | T2a | N0 | M0 | Grade 3 |
|  | **T2b** | **N0** | **M0** | **Grade 3** |
|  | **Any T** | **N1** | **M0** | **Any Grade** |
| IV | Any T | Any N | M1 | Any Grade |

## Definitions of regional lymph nodes in this study

In this study, regional lymph nodes are defined as follows in reference to the definitions of regional lymph nodes in skin tumors (UICC TNM 7th edition):

### Definitions of regional lymph nodes by site

Primary tumor in chest wall, back: Ipsilateral axillary lymph nodes

Primary tumor in upper limb: Ipsilateral medial epicondyle lymph nodes of humerus and axillary lymph nodes

Primary tumor in abdomen, loins, buttock: Ipsilateral inguinal lymph nodes

Primary tumor in lower limb: Ipsilateral popliteal and inguinal lymph nodes

### Definition of positive regional lymph nodes

A lymph node ≧10 mm in short diameter diagnosed by CT or MRI.

## Definition of resectable in this study.

"Resectabe" is defined as the absence of tumor invasion of critical organs, nerves, blood vessels, thoracic cavity, peritoneal cavity, etc. in diagnostic imaging, and it is judged that marginal resection or wide margin resection is expected during surgery. For patients with positive regional lymph nodes, resection of lymph node metastases is also considered "resectabe".

In the limb, the operative method can be either limb preserving surgery or amputation.

## Evaluation of surgical margin

Surgical margins in tumor resection are defined by the positional relationship between the tumor margin and the resection line.

In this study, surgical margins are comprehensively classified into one of the following three categories based on the intraoperative gross findings and the histopathological findings of the resected specimen:

| Intralesional margin | Surgical margin of which resection line penetrates the tumor parenchyma macroscopically |
| --- | --- |
| Marginal margin | Surgical margin of which resection line penetrates the reactive layer surrounding the tumor. Surgical margin of which resection line passes through just outside of the tumor margin, if reactive layer is absent or ambiguous. |
| Wide margin | Surgical margin of which resection line is outside of the peritumoral reactive layer throughout the circumference. However, if surgical margin contains a portion of normal tissue with 1 cm or less thickness (wide) around the area including the tumor and the reactive layer, it is judged as "inadequate wide excision"(wide margin - inadequate resection). |

Details on surgical margin evaluation are in compliance with "Surgical margin evaluation method for bone and soft tissue sarcoma (Edited by the Japanese Orthopaedic Association/Bone and Soft Tissue Tumor Committee). KANEHARA & Co., LTD 1989)

# Patients Selection Criteria

For inclusion in the study, patients must fulfill all of the following eligibility criteria and they are exluded if they meet any of the following exclusion criteria.

## Eligibility criteria

1. HistologicalIy proven non-round cell sarcoma^※^.

Any of the following histologic types (WHO-classification 2013 edition) and histological grade by FNCLCC system are Grade 2-3.

High-grade undifferentiated pleomorphic sarcoma (malignant fibrous histiocytoma: according to the 2002 edition of the WHO classification), fibrosarcoma, myxofibrosarcoma, leiomyosarcoma (conventional, poorly differentiated/pleomorphic/epithelioid), synovial sarcoma, liposarcoma (myxoid, high-grade myxoid, dedifferentiated, pleomorphic), pleomorphic rhabdomyosarcoma, malignant peripheral nerve sheath tumor (conventional, poorly differentiated), angiosarcoma (conventional, poorly differentiated/epithelioid), undifferentiated sarcoma/unclassifid sarcoma

- Histopathologically confirmed: In the primary case, definitive diagnosis of histologic type by incisional biopsy is mandatory. Local recurrence can be diagnosed by needle biopsy in addition to incisional biopsy at the time of initial onset.

1. Primary tumor or first local recurrent^※^ tumor

- Local recurrent tumor refers to the tumor of the same histology occurred in or around the site where the primary tumor was resected at the initial tumor resection

1. Ten or more unstained tumor tissue slides available for central review, whose biopsy specimen was used for the diagnosis described in 1) (in cases of local recurrence, submission of the pathological specimen at the initial diagnosis is mandatory, but he/she is eligible even if 10 unstained specimens are not available).
2. In the case of primary tumor, the patient has been diagnosed as T2bN0M0 or anyTN1M0 (UICC/AJCC 7th edition) by most recent imaging (chest CT, regional MRI: plain CT/MRI is acceptable) within 28 days prior to registration.
3. The largest diameter of the tumor should be the longest, whether transverse, coronal, or sagittal. However, in the case of N1, the primary lesion is of any size and depth.
4. N1 is defined as enlarged lymph nodes ≧10 mm in short diameter on CT or MRI of the regional lymph node area (see 3.3).
5. In case of primary local recurrence, there is no apparent distant metastasis by the most recent imaging examination (chest CT) within 28 days before registration. No limitation for tumor size and depth of primary tumor and recurrent tumor.
6. Tumor is in the extremities or trunk.

Extremities (upper limbs): shoulder, axilla, upper arm, elbow, forearm, wrist, or hand

Extremities (lower limbs: gluteal, inguinal, thigh, knee, lower leg, ankle, or foot

Trunk: the surface of the chest wall, abdominal wall, lumbar region, or back without tumor invasion to the thoracic cavity, abdominal cavity, or spinal canal.

1. Having measurable lesion^※^ on MRI axial section.

- Non-nodal lesion with a maximum diameter of 10 mm or greater on MRI with a slice thickness of 5 mm or less or nodal lesion with a short diameter of 15 mm or greater on MRI or CT with a slice thickness of 5 mm or less

1. Marginal resection or wide margin resection is considered feasible (see 3.4).
2. Age of the registration date is 20 years or older and 70 years or younger.
3. Performance status (PS) is 0 or 1 according to ECOG criteria.
4. There is no history of chemotherapy or radiation therapy, including treatment of the other cancer.
5. The most recent laboratory test within 14 days prior to registration (the same day of the week 2 weeks prior to the registration date is acceptable) meets all of the following:
6. Neutrophil count ≧1,500/mm^3^
7. Hemoglobin≧8.0 g/dL (no blood transfusions within 14 days prior to registration)
8. Platelet count ≧10×10^4^ / mm^3^
9. Total bilirubin ≦ 1.5 mg/dL
10. AST(GOT) ≦ 100 IU/L
11. ALT(GPT) ≦ 100 IU/L
12. Creatinine ≦ 1.5 mg/dL
13. Creatinine clearance^*^ ≧ 60 mL/min (estimated)

(Cockcroft-Gault's Ccr formula)

Male: {(140-Age) × Body weight (kg)}/{72 × Serum creatinine level (mg/dL)}

Females: 0.85×{(140-Age)×Body weight (kg)}/{72×Serum creatinine level (mg/dL)}

* If the estimated value by Cockcroft-Gault formula is less than 60 mL/min, he/she is eligible if the creatinine clearance calculated using 24-hour urine collection is 60 mL/min or more.

1. Normal or no change requiring treatment on the most recent 12-lead resting ECG within 28 days before registration (the same day of the week 4 weeks before registration is allowed).
2. Neither interstitial pneumonitis, pulmonary fibrosis, nor severe emphysema as diagnosed by chest CT is complicated.
3. Written informed consent was obtained.

## Exclusion criteria

1. Synchronous or metachronous (within 5 years) malignancies except for carcinoma in situ or intramucosal tumors curatively treated with local therapy
2. Active infection requiring systemic therapy
3. Body temperature >= 38 degrees Celsius
4. Women in pregnant, possibly pregnant or breast feeding
5. Psychiatric disease
6. Patients requiring systemic steroid medication
7. Unstable angina within 3 weeks, or with a history of myocardial infarction
8. Poorly controlled hypertension
9. Poorly controlled diabetes mellitus or routine administration of insulin
10. Positive HBs antigen

# Treatment Plan and Treatment Modification Criteria

Unless patient safety is threatened, treatment and treatment modifications is done in compliance with the specifications in this chapter.

## Protocol treatment

Protocol treatment is initiated within 7 days of registration.

If treatment is initiated after 8 days from registration for some reason, the reason should be documented on the Treatment Form. If it is determined that treatment cannot be initiated, describe the details in the Off-treatment Form as protocol treatment termination.

When laboratory parameters worsen and eligibility criteria are no longer met by the start of treatment after registration, the investigator/sub-investigator is allowed to decide whether initiate or terminate protocol treatment at their own discretion.

"6.3. Treatment modification criteria" is not applied at the beginning of the first course.

#### Drugs used

- Adriamycin (doxorubicin)^※^
- Ifosphamide^※^
- Gemcitabine^※^
- Docetaxicel^※^
- Entecavir, tenofovir disoproxil fumarate, tenofovir alafenamide fumarate

The use of generic drugs is not restricted.

- The company that manufactures or distributes these drugs, or intends to manufacture or sell these drugs, requires conflicts of interest control in the Clinical Trials Act as a company involved in this study (see 13.7.2.).

#### Arm A: standard treatment arm

Preoperative AI therapy (3 courses) plus tumor resection plus postoperative AI therapy (2 courses).

#### Arm B: experimental treatment arm

Preoperative GD therapy (3 courses) plus tumor resection plus postoperative GD therapy (2 courses).

- If margin evaluation in the resected specimen indicates inadequate margin (if the margin is positive on histology, intralesional excision, marginal excision, or wide excision for invasive disease resulting in a margin of less than 1 cm), then 2 courses of postoperative chemotherapy are followed by the additional radiotherapy as post-study treatment.


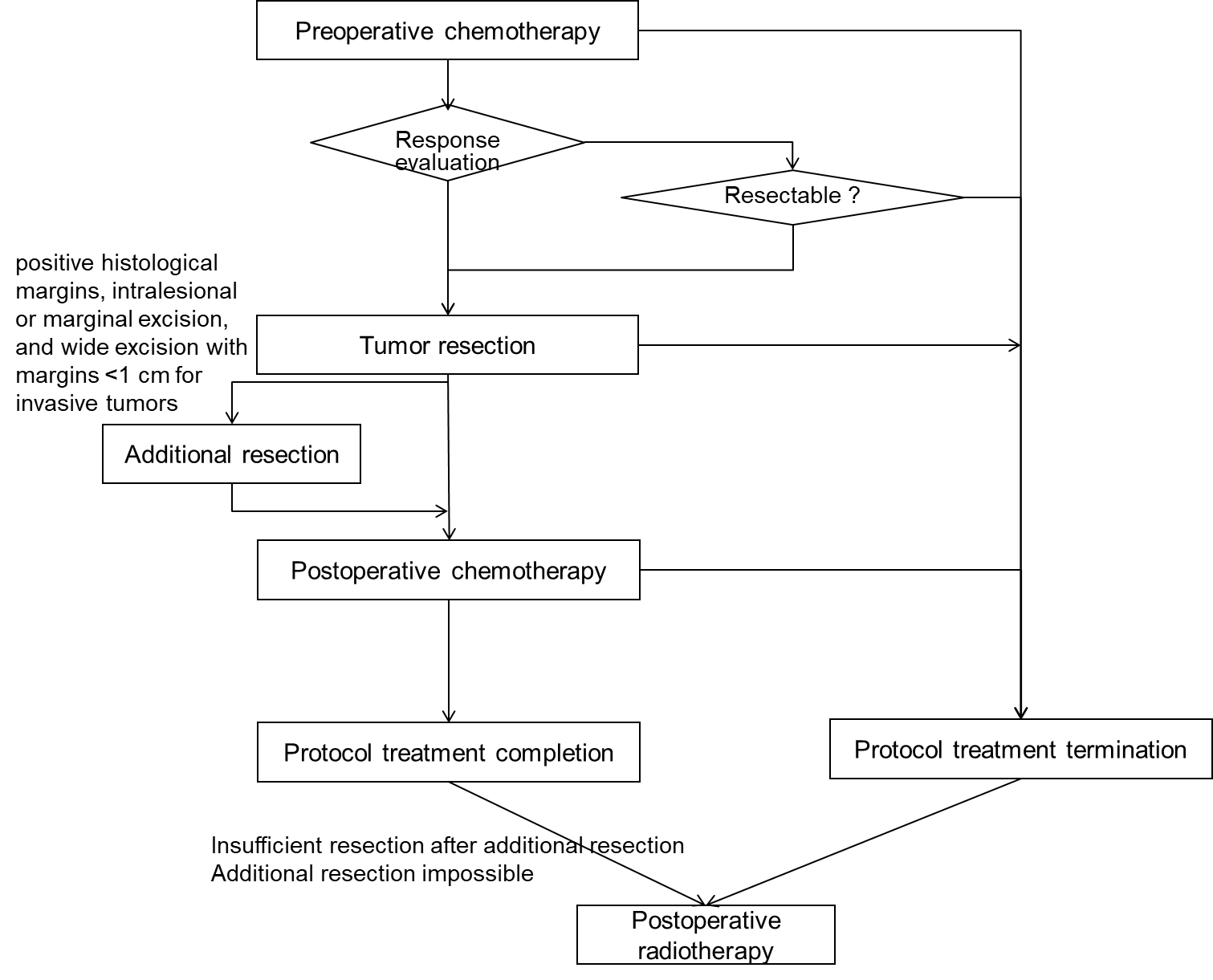


### Arm A: Preoperative AI therapy + tumor resection + postoperative AI therapy

#### 1) Preoperative chemotherapy

The following chemotherapy is administered for 3 courses as 1 course per 3 weeks.

| Drug | Dosage | Dosing regimen (dosing time) | Dose day |
| --- | --- | --- | --- |
| Adriamycin (ADM). | 30 mg/m^2^ | Div (2 h) | Day1, 2 |
| Ifosfamide (IFO) | 2 g/m^2^ | Div (4 h) | Day1-5 |

- One course is defined as the day of treatment initiation until the day before the next treatment initiation.
- The 3rd course of preoperative chemotherapy is to the day before the day of tumor resection.
- The administration of ADM and IFO is carried out in the hospitalization.
- Body surface area and drug dose calculations are institutional responsibilities, and the body surface area and drug dose conveyed by the Data Center at registration are only for double-checking with the physician's calculations. They should always be calculated and confirmed at the institution.
- Drug dose calculations should always be performed not only at registration but also before each course using the most recent body weight. However, no dose changes by recalculation are made during the course.
- When calculating the dose, the dose of IFO should be in units of 0.1 g, and the margins less than 0.1 g should be truncated.
- For dose of ADM, it should be in units of 1 mg, and the margins less than 1 mg should be truncated.
- Either of ADM or IFO may be given first. Though the infusion route is not specified, the administration by the central venous route is recommended.

**<Precautions for administration>**

**a) Adriamycin (ADM).**

- ADM is dissolved in 250-500 mL of normal saline and given by intravenous drip infusion over 2 hours.
- If the electrocardiogram abnormality is suspected or the patient complains of chest pain, administration of this drug should be carefully performed by using a heart rate monitor.

**b) Ifosfamide (IFO)**

- IFO is dissolved in 500-1,500 mL of normal saline and given by intravenous drip infusion over 4 hours.
- Mesna (uromitexan) is given to prevent hemorrhagic cystitis. 20% equivalent of the daily dose of IFO (round out in 100 mg units) is administered intravenously (IV or DIV) 3 times daily (immediately, 4, and 8 hours after IFO administration) on the day of IFO administration (day 1-day 5). The dosage can be modified as necessary. Alternatively, a continuous IV infusion of 60% equivalent of the daily IFO dose is administered from the initiation of IFO administration to 8 hours after the completion of administration.
- If occult blood in the urine is present, the volume of mesna should be increased to the same amount as the daily IFO dose. Observe the presence of urinary occult blood, gross hematuria, urinary frequency, and micturition pain as appropriate in order to prevent the aggravation of hemorrhagic cystitis.

**c) Infusion**

- 2,500 mL/m^2^/day or more of fluids are administered in day 1 - day 5. Types of intravenous fluids are not specified.
- More than 600 mL/m^2^/8 hours of urine output should be maintained for total of 8 hours from 2 hours before the start of IFO administration to 2 hours after the end of IFO administration.

#### 2) Tumor resection

① Timing of surgery

Patients who complete preoperative chemotherapy and patients who terminate preoperative chemotherapy for reasons other than Grade 4 non-hematological toxicities who are considered resectable by the treating physician will undergo tumor resection between day 1 and day 43 (6 weeks) of the last preoperative chemotherapy course. If preoperative chemotherapy is terminated followed by tumor resection, protocol treatment is terminated at the completion of surgery, and postoperative chemotherapy should not be given.

If the date of operation exceeds day 43 for any reason, the reason should be documented on the Treatment Form. However, if surgery cannot be performed until day 71 (within 10 weeks), the protocol treatment is terminated.

② Surgical procedure

a) Resection of primary tumor

Wide excision (en bloc resection with encapsulation of the tumor with healthy tissue outside the reactive layer) is the principle. In cases of extremity, whether it is the limb preservation method or amputation is not specified.

The extent of resection is determined by using MRI images after preoperative chemotherapy (the newest MRI image if preoperative chemotherapy is terminated and MRI is not performed after preoperative chemotherapy) to provide wide margins, including the area of secondary changes due to tumor.

b) Resection of recurrent tumor

Wide excision including resection of surgical scar by the initial surgery is the principle in the recurrence case within 4 years from the initial surgery.

In cases of recurrence more than 4 years after the initial surgery, the principle is to ignore the surgical scar at the time of the initial surgery and perform wide excision of the recurrent tumor only.

c) Resection of lymph node metastases

All enlarged regional lymph nodes on either imaging at registration or intraoperative macroscopic findings are resected. No lymph node dissection is performed for the lymph node with no enlargement both at registration and duing surgery.

③ Margin evaluation

In this study, the principle of resection is to ensure a healthy tissue thickness (wide) of 2 cm or more throughout the entire circumference of the tumor regardless with or without "barrier". However, if the tumor is judged to have a strong tendency to invade on imaging, the principle of resection is to ensure a healthy tissue thickness (wide) of at least 5 cm in the longitudinal direction (the site without barrier).

For further details, the Bone and Soft Tissue Sarcoma Margin Evaluation Method (Edited by the Japanese Orthopaedic Association/Bone and Soft Tissue Tumor Committee). KANEHARA & Co., LTD, 1989). Margin Evaluation Criteria in (6) (see 3.5. Margin evaluation).

④ Additional surgery/reoperation

- - 1. Additional surgery

If the initial surgery is inadequate (positive histological margins, intralesional or marginal excision, and wide excision with margins <1 cm for invasive tumors), as a rule, additional wide excision (with additional more extensive excision if the initial surgery is judged to have failed to achieve adequate wide margins) should be performed only before initiation of postoperative chemotherapy.

Additional surgery should be performed within 28 days from the previous surgery (counting the day of the previous surgery as day 0) after ensuring that the patient's general condition has fully recovered.

After initiation of postoperative chemotherapy, additional surgery is not allowed.

- - 1. Reoperation

Even during or after protocol treatment, reoperations (e.g., surgical for postoperative infection, surgery for fracture of reconstructive materials) with objective other than tumor resection are acceptable.

Additional surgeries as post-study treatment after completion or termination of the protocol treatment is not specified.

#### 3) Postoperative chemotherapy

AI therapy should be started after the initial surgery or within 5 weeks after the additional surgery (from day of surgery as day 0 and up to 35 days after surgery). The following chemotherapy is administered for 2 courses as 1 course per 3 weeks.

| Drug | Dosage | Dosing regimen (dosing time) | Dose day |
| --- | --- | --- | --- |
| Adriamycin (ADM) | 30 mg/m^2^ | Div (2 h) | Day1,2 |
| Ifosfamide (IFO) | 2 g/m^2^ | Div (4 h) | Day1-5 |

- One course is defined as the days from the day of the treatment initiation for each regimen to the day before the next treatment initiation.
- The 2nd course of postoperative chemotherapy is from the starting date of the course as day1 for a period of 21 days until day 21.
- Body surface area and drug dose calculations are institutional responsibilities, and the body surface area and drug dose conveyed by the Data Center at registration are only for double-checking with the physician's calculations. They should always be calculated and confirmed at the institution.
- Drug dose calculations should always be performed not only at registration but also before each course using the most recent body weight. However, no dose changes by recalculation are made during the course.
- When calculating the dose, the dose of IFO should be in units of 0.1 g, and the margins less than 0.1 g should be truncated.
- For dose of ADM, it should be in units of 1 mg, and the margins less than 1 mg should be truncated.
- Either of ADM or IFO may be given first. Though the infusion route is not specified, the administration by the central venous route is recommended.

**<Precautions for administration>**

**a) Adriamycin (ADM).**

- ADM is dissolved in 250-500 ml of normal saline and given by intravenous drip infusion over 2 hours.
- If the electrocardiogram abnormality is suspected or the patient complains of chest pain, administration of this drug should be carefully performed by using a heart rate monitor.

**b) Ifosfamide (IFO)**

- IFO is dissolved in 500-1500 ml of physiological saline and infused intravenously over 4 hours.
- Mesna (uromitexan) is given to prevent hemorrhagic cystitis. 20% equivalent of the daily dose of IFO (round out in 100 mg units) is administered intravenously (IV or DIV) 3 times daily (immediately, 4, and 8 hours after IFO administration) on the day of IFO administration (day 1-day 5). The dosage can be modified as necessary. Alternatively, a continuous IV infusion of 60% equivalent of the daily IFO dose is administered from the initiation of IFO administration to 8 hours after the completion of administration.
- If occult blood in the urine is present, the volume of mesna should be increased to the same amount as the daily IFO dose. Observe the presence of urinary occult blood, gross hematuria, urinary frequency, and micturition pain as appropriate in order to prevent the aggravation of hemorrhagic cystitis.

**c) Infusion**

- 2,500 mL/m^2^/day or more of fluids are administered in day 1-day 5. Types of intravenous fluids are not specified.
- More than 600 mL/m^2^/8 hours of urine output should be maintained for total of 8 hours from 2 hours before the start of IFO administration to 2 hours after the end of IFO administration.

### Arm B: Preoperative GD therapy + tumor resection + postoperative GD therapy

#### 1) Preoperative chemotherapy

The following chemotherapy is administered for 3 courses as 1 course per 3 weeks.

| Drug | Dosage | Dosing regimen (dosing time) | Dose day |
| --- | --- | --- | --- |
| Gemcitabine (GEM) | 900 mg/m^2^ | Div (30 min) | Day 1, 8 |
| Docetaxel (DOC) | 70 mg/m^2^ | Div (1 h) | Day 8 |

- One course is defined as the day of treatment initiation until the day before the next treatment initiation.
- The 3rd course of preoperative chemotherapy is to the day before the day of tumor resection.
- GEM and DOC are administered on an inpatient or outpatient basis.
- Body surface area and drug dose calculations are institutional responsibilities, and the body surface area and drug dose conveyed by the Data Center at registration are only for double-checking with the physician's calculations. They should always be calculated and confirmed at the institution.
- Drug dose calculations should always be performed not only at registration but also before each course using the most recent body weight. However, no dose changes by recalculation are made during the course.
- When calculating the dose, the dose of GEM should be by 10 mg units, and the margin of less than 10 mg should be truncated. For DOC, the dose should be by 1 mg units, and the margin of less than 1 mg should be truncated.
- Either GEM or DOC is given first in day 8. The infusion route is not specified.

**<Precautions for administration>**

**a) Gemcitabine (GEM)**

- GEM is dissolved in 100-250 mL of normal saline and given by intravenous drip infusion over 30 minutes.

**b) Docetaxel (DOC)**

- - DOC will be dissolved in 250-500 mL of normal saline after adjustment according to the package insert and infused intravenously over 1 hour.
  - However, since the accompanying dissolving solution of DOC contains alcohol, when this product is administered to patients with alcohol hypersensitivity, it should be dissolved in 5% glucose solution or normal saline.
- Premedication with corticosteroids is performed in order to relieve oedema and hypersensitivity symptoms. As a premedication, dexamethasone (16 mg/day, 8 mg twice daily) etc. is administered orally for 3 days before administration of DOC. Instead of oral administration, dexamethasone phosphate (20 mg/day, 10 mg BID) may be given by drip infusion.

#### 2) Tumor resection

① Timing of surgery

Patients who complete preoperative chemotherapy and patients who terminate preoperative chemotherapy for reasons other than Grade 4 non-hematological toxicities who are considered resectable by the treating physician will undergo tumor resection between day 1 and day 43 (6 weeks) of the last preoperative chemotherapy course. If preoperative chemotherapy is terminated followed by tumor resection, protocol treatment is terminated at the completion of surgery, and postoperative chemotherapy should not be given.

If the date of operation exceeds day 43 for any reason, the reason should be documented on the Treatment Form. However, if surgery cannot be performed until day 71 (within 10 weeks), the protocol treatment is terminated.

② Surgical procedure

a) Resection of primary tumor

Wide excision (en bloc resection with encapsulation of the tumor with healthy tissue outside the reactive layer) is the principle. In cases of extremity, whether it is the limb preservation method or amputation is not specified.

The extent of resection is determined by using MRI images after preoperative chemotherapy (the newest MRI image if preoperative chemotherapy is terminated and MRI is not performed after preoperative chemotherapy) to provide wide margins, including the area of secondary changes due to tumor.

b) Resection of recurrent tumor

Wide excision including resection of surgical scar by the initial surgery is the principle in the recurrence case within 4 years from the initial surgery.

In cases of recurrence more than 4 years after the initial surgery, the principle is to ignore the surgical scar at the time of the initial surgery and perform wide excision of the recurrent tumor only.

c) Resection of lymph node metastases

All enlarged regional lymph nodes on either imaging at registration or intraoperative macroscopic findings are resected. No lymph node dissection is performed for the lymph node with no enlargement both at registration and duing surgery.

③ Margin evaluation

In this study, the principle of resection is to ensure a healthy tissue thickness (wide) of 2 cm or more throughout the entire In this study, the principle of resection is to ensure a healthy tissue thickness (wide) of 2 cm or more throughout the entire circumference of the tumor regardless with or without "barrier". However, if the tumor is judged to have a strong tendency to invade on imaging, the principle of resection is to ensure a healthy tissue thickness (wide) of at least 5 cm in the longitudinal direction (the site without barrier).

For further details, the Bone and Soft Tissue Sarcoma Margin Evaluation Method (Edited by the Japanese Orthopaedic Association/Bone and Soft Tissue Tumor Committee). KANEHARA & Co., LTD, 1989). Margin Evaluation Criteria in (6) (see 3.5. Margin evaluation).

④ Additional surgery/reoperation

1. Additional surgery

If the initial surgery is inadequate (positive histological margins, intralesional or marginal excision, and wide excision with margins <1 cm for invasive tumors), as a rule, additional wide excision (with additional more extensive excision if the initial surgery is judged to have failed to achieve adequate wide margins) should be performed only before initiation of postoperative chemotherapy.

Additional surgery should be performed within 28 days from the previous surgery (counting the day of the previous surgery as day 0) after ensuring that the patient's general condition has fully recovered.

After initiation of postoperative chemotherapy, additional surgery is not allowed.

1. Reoperation

Even during or after protocol treatment, reoperations (e.g., surgical for postoperative infection, surgery for fracture of reconstructive materials) with objective other than tumor resection are acceptable.

Additional surgeries as post-study treatment will not be specified Additional surgeries as post-study treatment after completion or termination of the protocol treatment is not specified.

#### 3) Postoperative chemotherapy

GD therapy should be started after the initial surgery or within 5 weeks after the additional surgery (from day of surgery as day 0 and up to 35 days after surgery). The following chemotherapy is administered for 2 courses as 1 course per 3 weeks.

| Drug | Dosage | Dosing regimen (dosing time) | Dose day |
| --- | --- | --- | --- |
| Gemcitabine | 900 mg/m^2^ | Div (30 min) | Day 1, 8 |
| Docetaxel | 70 mg/m^2^ | Div (1 h) | Day 8 |

- One course is defined as the days from the day of the treatment initiation for each regimen to the day before the next treatment initiation.
- The 2nd course of postoperative chemotherapy is from the starting date of the course as day1 for a period of 21 days until day 21.
- Drug dose calculations should always be performed not only at registration but also before each course using the most recent body weight. However, no dose changes by recalculation are made during the course.
- Chemotherapy for arm B is performed on an inpatient or outpatient basis.
- When calculating the dose, the dose of GEM should be by 10 mg units, and the margin of less than 10 mg should be truncated.
- For DOC, the dose should be by 1 mg units, and the margin of less than 1 mg should be truncated.
- Either GEM or DOC is given first in day 8. The infusion route is not specified.

**<Precautions for administration>**

**a) Gemcitabine (GEM)**

- GEM is dissolved in 100-250 mL of normal saline and infused over 30 min.

**b) Docetaxel (DOC)**

- DOC will be dissolved in 250-500 mL of normal saline after adjustment according to the package insert and infused intravenously over 1 hour.
- However, since the accompanying dissolving solution of DOC contains alcohol, when this product is administered to patients with alcohol hypersensitivity, it should be dissolved in 5% glucose solution or normal saline.
- Premedication with corticosteroids is performed in order to relieve oedema and hypersensitivity symptoms. As a premedication, dexamethasone (16 mg/day, 8 mg twice daily) etc. is administered orally for 3 days before administration of DOC. Instead of oral administration, dexamethasone phosphate (20 mg/day, 10 mg BID) may be given by drip infusion.

## Protocol Treatment Termination/Completion Criteria

### Definition of protocol treatment completion

#### 1) Definition of completion of preoperative chemotherapy

Arm A: Completion of preoperative chemotherapy with completion of 3 courses of preoperative chemotherapy (completion of IFO in day 5).

Arm B: Completion of preoperative chemotherapy with completion of 3 courses of preoperative chemotherapy (completion of GEM and DOC in day 8).

#### 2) Definition of surgery completion

Surgery is completed with the date of surgery if evaluated margin is marginal margin or wide margin.

If additional surgery is performed, the evaluated final margin is marginal margin or wide margin, the surgery is completed at the date of additional surgery.

#### 3) Definition of postoperative chemotherapy completion

Arm A: Postoperative chemotherapy is completed by the completion of 2 courses of postoperative chemotherapy (completion of day 5 IFOs).

Arm B: Postoperative chemotherapy is completed by the completion of 2 courses of postoperative chemotherapy (GEM and DOC in day8) .

#### 4) Definition of protocol treatment completion

Arm A: Completion of all of preoperative chemotherapy, surgery and postoperative chemotherapy is defined as protocol treatment completion.

Arm B: Completion of all of preoperative chemotherapy, surgery and postoperative chemotherapy is defined as protocol treatment completion.

### Criteria for termination of protocol treatment

#### 1) Criteria for termination of preoperative chemotherapy

If the patient meets any of the following 1)-13) after starting preoperative chemotherapy, then terminate preoperative chemotherapy.

In the case of termination of preoperative chemotherapy, the protocol treatment should be continued, response evaluation should be performed whenever possible, and surgery should be performed. However, no postoperative chemotherapy should be administered, and the protocol treatment should be terminated after completion of surgery.

1. Preoperative chemotherapy was considered ineffective.

Apparent disease progression after initiation of preoperative chemotherapy.

1. Any of the following Grade 3 adverse event is observed.
2. Supraventricular tachycardia
3. Ventricular arrhythmia
4. Left ventricular systolic dysfunction
5. Any of the following Grade 2 or Grade 3 adverse event is observed.
6. Vertigo, dizziness
7. Depressed level of consciousness
8. Seizures [Brief generalized seizure]
9. Leukoencephalopathy [Moderate symptoms; focal T2/FLAIR hyperintensities, involving periventricular white matter extending into centrum semiovale or involving 1/3 to 2/3 of susceptible areas of cerebrum +/- moderate increase in SAS and/or moderate ventriculomegaly]
10. Grade 2 pneumonitis (interstitial pneumonitis)
11. Grade 3 peripheral sensory/motor neuropathy
12. Grade 2-4 creatinine increased
13. Adverse events other than the treatment modification criteria that the treating physician judges to require termination of protocol treatment
14. The start of the course is delayed for more than 2 weeks (14 days) from the expected start date of the course (day 22 in the previous course).
15. In arm B, the administration in day 8 (GEM, DOC) is delayed for more than 2 weeks from the expected date of day 8.
16. Grade 3 hematuria leading to suspension of chemotherapy and failure to recover to Grade 0-1 within 3 days including the first day of suspension
17. Hematuria results in suspension of chemotherapy and Grade 3 hematuria reappears after resuming
18. The dose reduction/termination criteria are met after the dose is reduced to level -2 (minus 2).
19. Patient proposes the termination of chemotherapy alone (distinguish whether associated with adverse event or not)

**2) Protocol termination criteria (all treatments)**

Protocol treatment is terminated in any of the following cases:

1. The protocol treatment is judged to be ineffective by any of the following:
   1. Disease progression is observed after the start of protocol treatment

※ Protocol treatment should not be terminated if response evaluation imaging indicates PD but continuing protocol treatment is considered clinically appropriate.

Terminate protocol treatment after completion of surgery, if resectable.

- 1. Margin evaluation indicates intralesional excision and appropriate additional surgery is not possible.
  2. Judged as intralesional excision by margin evaluation of additional resection.
  3. Judged that surgical cannot be continued for some reason.

1. Protocol treatment cannot be continued due to adverse events.
2. Grade 4 non-hematological toxicity is observed.

(Non-hematological toxicity: Adverse event other than anemia, bone marrow hypocellular, lymphocyte count decreased, neutrophil count decreased, white blood cell decreased, platelet count decreased, and CD4 lymphocyte decreased in CTCAE v4.0-JCOG).

However, i) and ii) below are excluded.

i) Adverse events associated with surgery

Intraoperative skin injury; Intraoperative musculoskeletal injury; Wound complication; Wound dehiscence; Bone infection; Soft tissue infection; Wound infection; Intraoperative neurological injury; Intraoperative arterial injury; Intraoperative venous injury; Intraoperative hemorrhage; Postoperative hemorrhage; Joint infection

ii) Serum electrolyte abnormality

hypernatremia, hyponatremia, hyperkalemia, hypokalemia, hypercalcemia, Hypocalcemia

1. Grade 2 pneumonitis (interstitial pneumonitis) after initiation of postoperative chemotherapy
2. Grade 3 supraventricular tachycardia and ventricular arrhythmia after initiation of postoperative chemotherapy
3. Protocol treatment could not be initiated within the specified time periods (6.1 and 6.3) due to adverse events
4. The criteria for terminating protocol treatment in the treatment modification criteria (6.3.) are met.
5. Adverse events other than the treatment modification criteria that the investigator/sub-investigator judges to require termination of protocol treatment
6. The patient offers termination of protocol treatment because of reasons not associated with adverse events

- This category should be used if an association with an adverse event cannot be ruled out.

1. The patient offers termination of protocol treatment because of reasons not associated with adverse events

- Patient refusal of protocol treatment after registration and before initiation of protocol treatment
- Patient refusal by the reason definely not associated with adverse events, such as a patient's or family member's relocation during protocol treatment

1. Death during protocol treatment

- Death before deciding to terminate protocol treatment for other reasons

1. Others: progression occurs before the start of protocol treatment after registration (protocol treatment could not be initiated due to rapid progression), protocol violation is identified, or ineligibility is determined due to changes in pathological diagnosis after registration and treatment was changed.

The date of termination of protocol treatment is the date of death in case of 5), the date of completion of surgery if the patient meets the criteria for terminating preoperative chemotherapy (6.2.2.1), or the date that the treating physician determines that protocol treatment is terminated.

In order to perform a central pathological diagnosis in this study, the central pathological diagnosis may reveal ineligibility during the protocol treatment. In such cases, Study Coordinator and the investigator will discuss the response. If termination of protocol treatment is judged to be appropriate, protocol treatment is terminated, and the reason for termination will be "Others".

## Treatment modification criteria

The following terms shall be used for the treatment modification.

Termination: Discontinuation of a part of or all of the treatment without restarting.

Delay: Delay the start of the course or administration of treatment from the planned date.

Suspending: temporary interruptions or withdrawals that may be resumed if conditions are met

Skip: Do not administer one or more drugs and proceed to the next schedule.

"Infection" in this chapter refers to the following.

Infection: CTCAEv4.0 infections and infestations

Bronchial infection; Lung infection; Bone infection; Soft tissue infection

### Arm A (ADM+IFO therapy) dose levels.

| Drug | Dose level | Dosage and administration methods | Dose day |
| --- | --- | --- | --- |
| ADM | Level 0 (initial dose) | 30 mg/m^2^ IV | Day 1, 2 |
|  | Level-1 (minus 1). | 24 mg/m^2^ IV | Day 1, 2 |
|  | Level-2 (minus 2) | 19 mg/m^2^ IV | Day 1, 2 |
| IFO | Level 0 (initial dose) | 2 g/m^2^ IV | Day 1-5 |
|  | Level-1 (minus 1). | 1.6 g/m^2^ IV | Day 1-5 |
|  | Level-2 (minus 2) | 1.3 g/m^2^ IV | Day 1-5 |

### Arm A (ADM + IFO therapy) course initiation criteria (common in pre- and post-operative chemotherapy)

Initiate the course after ensuring that all of the following are met with the most recent laboratory data within 3 days before the start of the course: If any is not met, the initiation of the course is delayed on a daily basis.

Chemotherapy is terminated if the course delay exceeds 2 weeks from the scheduled start date of the course (day 22 from the start date of the previous course).

Course initiation criteria is applied to all courses of preoperative and postoperative chemotherapy after the 2nd course of preoperative chemotherapy.

1) All of the following ①-⑮ are met: However, if G-CSF is used in the previous course, ② should have been assessed at least 48 hours after the last dose of G-CSF. Transfusion is not restricted.

① Hemoglobin Grade 0-2 (≧8 g/dL)

② Neutrophil count Grade 0-1(≧1,500/mm^3^)

③ Platelets count Grade 0-2 (≧50,000 /mm^3^).

1. AST (GOT) Grade 0-1(≦90 IU/L)
2. ALT (GPT) Grade 0-1 (male: ≦126 IU/L, female: ≦69 IU/L).
3. Serum creatinine Grade 0-1 (male: ≦1.605 mg/dL, female: ≦1.185 mg/dL).
4. Fatigue Grade 0-2
5. Edema limbs Grade 0-2
6. Diarrhea Grade 0-1
7. Hematuria Grade 0-1
8. Oral mucositis Grade 0-1
9. Supraventricular tachycardia Grade 0-1
10. Ventricular arrhythmia Grade 0-1
11. Pneumonitis Grade 0-1
12. Infection Grade 0-1

### Arm A (ADM + IFO therapy) Dose reduction/termination criteria (common in preoperative and postoperative chemotherapy)

If any of the following adverse events are observed during the course of chemotherapy, whether preoperatively or postoperatively, treatment modification is made according to the dose reduction/termination criteria. Treatment modification is not made during the course. The 1st course of postoperative chemotherapy is modified according to adverse events during the 3rd course of preoperative chemotherapy.

If two or more dose reduction criteria are met for one drug at the beginning of the course, a lower dose level is applied. During the protocol treatment, including the starting dose of the 1st course of postoperative chemotherapy after the 3rd course of preoperative chemotherapy, no re-escalation (increasing the dose level) is performed even if the adverse events responsible for the dose reduction have recovered or the adverse events have not appeared after the dose has been reduced (lowering the dose level).

If the dose reduction/termination criteria are met after reducing to level -2 (minus 2), subsequent chemotherapy is terminated.

In the case of termination of preoperative chemotherapy, the protocol treatment should be continued, response evaluation should be performed whenever possible and surgical should be performed. However, postoperative chemotherapy should not be performed, and the protocol treatment should be terminated after completion of surgery.

Table 6.3.3. Dose Reduction/Termination Criteria

| Adverse event | Grade | ADM | IFO |
| --- | --- | --- | --- |
| Neutrophil count (no G-CSF prophylaxis) | Grade 4 (lasting 5 days or more)  <500/mm^3^ | No reduction  (G-CSF prophylaxis) | No reduction  (G-CSF prophylaxis) |
| Neutrophil count (with G-CSF prophylaxis) | Grade 4 (lasting 5 days or more)  <500/mm^3^ | Loss on one level | 1 level dose down |
| Diarrhea | Grade 3 | 1 level dose down | 1 level dose down |
| Oral mucositis | Grade 3 | 1 level dose down | 1 level dose down |
| Infection | Grade 3 | 1 level dose down | 1 level dose down |
| Pneumonitis | Grade 2 | Termination | Termination |
| Serum creatinine | Grade2-4  (male: >1.605 mg/dL,  female: >1.185 mg/dL). | Termination | Termination |
| Supraventricular tachycardia | Grade 2 (appeared twice) | Termination | Termination |
|  | Grade 3 |  |  |
| Atrial arrhythmia | Grade 2 (appeared twice) | Termination | Termination |
|  | Grade 3 |  |  |
| Left ventricular systolic dysfunction | Grade 2 (appeared twice) | Termination | Termination |
|  | Grade 3 |  |  |
| Vertigo, dizziness | Grade 2-3 | Termination | Termination |
| Depressed level of consciousness | Grade 2-3 | Termination | Termination |
| Seizures | Grade 2-3 | Termination | Termination |
| Leukoencephalopathy | Grade 2-3 | Termination | Termination |
| Peripheral sensory/motor neuropathy | Grade 3 | Termination | Termination |
| Hematuria (See 6.3.4.1) | Grade 3 (appeared twice*) | Termination | Termination |
| Other non-hematological toxicity ** | Grade 4 | Termination | Termination |
| When the above adverse events occur at dose level -2 (minus 2) | | Termination | Termination |

* However, if hematuria does not resolve to Grade 0-1 within 3 days after suspension due to Grade 3 hematuria, chemotherapy should be terminated.

** Non-hematological toxicity: Adverse event other than anemia, bone marrow hypocellular, lymphocyte count decreased, neutrophil count decreased, white blood cell decreased, platelet count decreased, and CD4 lymphocyte decreased in CTCAE v4.0-JCOG.

Except for the following adverse events:

hypernatremia, hyponatremia, hyperkalemia, hypokalemia, hypercalcemia, hypocalcemia

** If applicable, the specific adverse event(s) should be describled in the CRFs.

#### 1) Secondary prophylaxis with G-CSF

If leukopenia or neutropenia is Grade 4 lasting at least 5 days (all blood tests are Grade 4, which are performed between the initial test with Grade 4 and the test at 5 days after the initial test, regardless of the frequency of tests and inter-assay intervals), secondary prophylaxis with G-CSF is given for all subsequent courses unless G-CSF prophylaxis is already given (See 6.4.5.2). Dose modification is not made for both ADM and IFO.

If secondary prophylaxis with G-CSF has been performed, reduce the dose level by one for both ADM and IFO from the next course.

Secondary G-CSF prophylaxis consists of G-CSF administration (filgrastim 50 μg/m^2^ subcutaneously (or 100 μg/m^2^ intravenously) or lenograstim 2 μg/kg subcutaneously (or 5 μg/kg intravenously) or naltograstim 1 μg/kg subcutaneously (or 2 μg/kg intravenously) once daily for 7 consecutive days from day 7 of the course. If pegfilgrastim is used, 3.6 mg per course is given subcutaneously once on day 7 of the course. If IFO is suspended during the course and the date of the final dose of IFO is day 6 or later, the starting day of administration of G-CSF is 2 days after the date of the last dose of IFO.

If the therapeutic administration of G-CSF criteria (see 6.4.5.3) is met after completion of secondary prophylaxis during the course with G-CSF prophylaxis, secondary prophylaxis is followed by prolongation of daily G-CSF to the time of discontinuation as defined in 6.4.5.3.

### Arm A (ADM + IFO therapy) preoperative and postoperative chemotherapy suspension and restart criteria

ADM and IFO are suspended if any of the following adverse events occur after each course of treatment is initiated because course initiation criteria has been met.

1. Hematuria

Chemotherapy (IFO and ADM) is immediately suspended if Grade 3 hematuria (persistent macroscopic bleeding or clots; requiring catheters or devices inserted or transfusion) appears on the day of IFO administration (day 1-5).

If hematuria recovers to Grade 0-1 (microscopic hematuria) within 3 days, administration should be restarted. When restarting, do not administer the remaining drug on the day of suspension, and restart the administration from the next day.

Chemotherapy is terminated if hematuria does not recover to Grade 0-1 within 3 days. Chemotherapy is terminated if Grade 3 hematuria reappears after suspension and resumption.

1. Cardiotoxicity

If Grade 2 arrhythmias (supraventricular tachycardia, paroxysmal atrial tachycardia, ventricular arrhythmia, Mobitz (type) II atrioventricular block) appear during ADM or IFO administration, immediately suspend all drug being delivered. If the arrhythmia resolves quickly, administration is resumed using ECG monitors, but after resuming, the same course of treatment is discontinued and chemotherapy is terminated if Grade 2 arrhythmia develops again.

If Grade 2 arrhythmias appear during the previous course, no dose reduction of subsequent chemotherapy is made, but treatment is continued on subsequent courses with attention to arrhythmias using ECG monitors.

Again, if Grade 2 arrhythmias appear, the same course of treatment is discontinued and chemotherapy is terminated.

If Grade 3 arrhythmias (supraventricular tachycardia, paroxysmal atrial tachycardia, ventricular arrhythmias, and Mobitz (type) II atrioventricular block) appear during chemotherapy, the administration is immediately discontinued and the chemotherapy is terminated.

1. Grade 3 infection

If Grade 3 infection develops during ADM or IFO administration, chemotherapy is immediately suspended and not resumed within the course. From the next course, the dose level of both ADM and IFO are reduced by 1 level.

### Arm B (GEM+DOC therapy) dose levels

| Drug | Dose level | Dosage and administration methods | Dose day |
| --- | --- | --- | --- |
| GEM | Level 0 (initial dose) | 900 mg/m^2^ IV | Day 1, 8 |
|  | Level-1 (minus 1) | 720 mg/m^2^ IV | Day 1, 8 |
|  | Level-2 (minus 2) | 570 mg/m^2^ IV | Day 1, 8 |
| DOC | Level 0 (initial dose) | 70 mg/m^2^ IV | Day 8 |
|  | Level -1 (minus 1) | 56 mg/m^2^ IV | Day 8 |
|  | Level -2 (minus 2) | 44 mg/m^2^ IV | Day 8 |

### Arm B (GEM+DOC therapy) course initiation criteria (common in pre- and post-operative chemotherapy)

Initiate the course after ensuring that all of the following are met with the most recent laboratory data within 3 days before the start of the course: If any is not met, the initiation of the course is delayed on a daily basis.

However, if G-CSF is used in the previous course, ② should have been assessed at least 48 hours after the last dose of G-CSF. Transfusion is not restricted.

Chemotherapy is terminated if the course delay exceeds 2 weeks from the scheduled start date of the course (day 15 with the day of the previous course day 8 as day 1 of the current course).

Course initiation criteria is applied to all courses of preoperative and postoperative chemotherapy after the 2nd course of preoperative chemotherapy.

If Grade 1 pneumonitis is present, percutaneous oxygen-saturation SpO_2_ and chest radiograph (2 directions) should be obtained at least weekly to ensure no exacerbation of pneumonitis.

If the patient is considered to have exacerbation of pneumonitis, even if no symptoms are observed, the subsequent administration of the drug should be terminated. If there is no exacerbation, continue chemotherapy after a one-level dose reduction.

① Hemoglobin Grade 0-2 (≧8 g/dL)

② Neutrophil count Grade 0-2(≧1,000/mm^3^)

③ Platelets count Grade 0-2 (≧5×10^4^/mm^3^)

1. AST(GOT) Grade 0-1(≦90 IU/L)
2. ALT(GPT) Grade 0-1 (male: ≦126 IU/L, female: ≦69 IU/L).
3. Serum creatinine Grade 0-1 (male: ≦1.605 mg/dL, female: ≦1.185 mg/dL).
4. Fatigue Grade 0-2
5. Edema limbs Grade 0-2
6. Diarrhea Grade 0-1
7. Hematuria Grade 0-1
8. Oral mucositis Grade 0-1
9. Supraventricular tachycardia Grade 0-1
10. Ventricular arrhythmia Grade 0-1
11. Pneumonitis Grade 0-1
12. Infection Grade 0-1

### Arm B (GEM+DOC therapy) day 8 dosing criteria

After confirming that all of the following are met, day 8 of GEM and DOC are administered.

If the dosing criteria are not met, the administration of day 8 should be delayed for up to 2 weeks. If day 8 is delayed, the plan starting date of the next course will be day 15, with the actual day day 8 was administered as day 1. If day 8 dose cannot be done for more than 2 weeks (if it exceeds day 22 counted from day 1 of the last course), chemotherapy is terminated.

If day 8 GEM+ DOC dose of the 3rd course or preoperative chemotherapy could not be performed, tumor resection should be performed before day 43 counting from day 1 of the 3rd course.

If Grade 1 pneumonitis is observed, percutaneous oxygen-saturation SpO_2_ and chest radiograph (2 directions) should be performed at least weekly to ensure that there is no exacerbation of pneumonitis

1. All of the following are met with the most recent laboratory data within 3 days before the day of administration.
2. Hemoglobin Grade 0-2 (≧8 g/dL)
3. Neutrophil count Grade 0-2 (≧1,000/mm^3^)
4. Platelet count Grade 0-2 (≧5×10^4^/mm^3^)
5. AST (GOT) Grade 0-1(≦90 IU/L)
6. ALT (GPT) Grade 0-1 (male: ≦126 IU/L, female: ≦69 IU/L).
7. The following is met on the day of administration.
8. Pneumonitis Grade 0-1

### Arm B (GEM + DOC therapy) Dose reduction/termination criteria (common in preoperative and postoperative chemotherapy)

If any of the following adverse events are observed during the course of chemotherapy, whether preoperatively or postoperatively, treatment modification is made according to the dose reduction/termination criteria. Treatment modification is not made during the course. The 1st course of postoperative chemotherapy is modified according to adverse events during the 3rd course of preoperative chemotherapy.

If two or more dose reduction criteria are met for one drug at the beginning of the course, a lower dose level is applied. During the protocol treatment, including the starting dose of the 1st course of postoperative chemotherapy after the 3rd course of preoperative chemotherapy, no re-escalation (increasing the dose level) is performed even if the adverse events responsible for the dose reduction have recovered or the adverse events have not appeared after the dose has been reduced (lowering the dose level).

If the dose reduction/termination criteria are met after reducing to level -2 (minus 2), subsequent chemotherapy is terminated.

In the case of termination of preoperative chemotherapy, the protocol treatment should be continued, response evaluation should be performed whenever possible and surgical should be performed. However, postoperative chemotherapy should not be performed, and the protocol treatment should be terminated after completion of surgery.

Table 6.3.8. Dose Reduction/Termination Criteria

| Adverse event | Grade | GEM | DOC |
| --- | --- | --- | --- |
| Neutrophil count (no G-CSF prophylaxis) | Grade 4 (lasting 5 days or more)  <500/mm^3^ | No reduction  (G-CSF prophylaxis) | No reduction  (G-CSF prophylaxis) |
| Neutrophil count (with G-CSF prophylaxis) | Grade 4 (lasting 5 days or more)  <500/mm^3^ | 1 level dose down | 1 level dose down |
| Diarrhea | Grade 3 | 1 level dose down | 1 level dose down |
| Oral mucositis | Grade 3 | 1 level dose down | 1 level dose down |
| Infection | Grade 3 | 1 level dose down | 1 level dose down |
| Pneumonitis | Grade 1 | 1 level dose down | 1 level dose down |
|  | Grade 2 | Termination | Termination |
| Peripheral sensory/motor neuropathy | Grade 2 | 1 level dose down | 1 level dose down |
|  | Grade 3 | Termination | Termination |
| Serum creatinine | Grade 2-4  (male: >1.605 mg/dL,  female: >1.185 mg/dL). | Termination | Termination |
| Supraventricular tachycardia | Grade 2 (appeared twice) | Termination | Termination |
|  | Grade 3 |  |  |
| Atrial arrhythmia | Grade 2 (appeared twice) | Termination | Termination |
|  | Grade 3 |  |  |
| Left ventricular systolic dysfunction | Grade 2 (appeared twice) | Termination | Termination |
|  | Grade 3 |  |  |
| Vertigo, dizziness | Grade 2-3 | Termination | Termination |
| Depressed level of consciousness | Grade 2-3 | Termination | Termination |
| Seizures | Grade 2-3 | Termination | Termination |
| Leukoencephalopathy | Grade 2-3 | Termination | Termination |
| Other non-hematological toxicity** | Grade 4 | Termination | Termination |
| When the above adverse events occur at dose level-2 (minus 2) | | Termination | Termination |

** Non-hematological toxicity: Adverse event other than anemia, bone marrow hypocellular, lymphocyte count decreased, neutrophil count decreased, white blood cell decreased, platelet count decreased, and CD4 lymphocyte decreased in CTCAE v4.0-JCOG

Except for the following adverse events:

hypernatremia, hyponatremia, hyperkalemia, hypokalemia, hypercalcemia, hypocalcemia

** If applicable, the specific adverse event(s) should be describled in the CRFs.

#### 1) Secondary prophylaxis with G-CSF

If leukopenia or neutropenia is Grade 4 lasting at least 5 days (all blood tests are Grade 4, which are performed between the initial test with Grade 4 and the test at 5 days after the initial test, regardless of the frequency of tests and inter-assay intervals), secondary prophylaxis with G-CSF is given for all subsequent courses unless G-CSF prophylaxis is already given (See 6.4.5. 2) . Dose modification is not made for both GEM and DOC.

If secondary prophylaxis with G-CSF has been performed, reduce the dose level by one for both GEM and DOC from the next course.

Secondary prophylaxis with G-CSF is G-CSF (filgrastim 50 μg/m^2^ subcutaneously (or 100 μg/m^2^ intravenously) or lenograstim 2 μg/kg subcutaneously (or 5 μ/kg intravenously) or naltograstim 1 μg/kg subcutaneously (or 2 μg/kg intravenously)) once daily for 7 consecutive days from courses of day10 (2 days after the day of GEM+DOC administration). If pegfilgrastim is used, 3.6 mg per course is given subcutaneously once on day 10 the course. If GEM+DOC administration is delayed during the course and the date of GEM+DOC administration is day 9 or later, the starting day of G-CSF administration is 2 days after the date of GEM+DOC administration.

If the therapeutic administration of G-CSF criteria (see 6.4.5.3) is met after completion of secondary prophylaxis during the course secondary prophylaxis with G-CSF, secondary prophylaxis is followed by prolongation of daily G-CSF to the time of discontinuation as defined in 6.4.5.3.

### Surgical Indication Criteria (arm A & arm B)

After completion of preoperative chemotherapy or termination of preoperative chemotherapy for reasons other than Grade 4 non-hematological toxicity, if the treating physician judges resectable, perform surgery after confirming that the most recent laboratory data within 7 days before the day of surgery meet all of the following 1) to 7). Transfusion-induced increases in hemoglobin are acceptable. However, if G-CSF is used, ② should have been administered for at least 48 hours after the last dose of G-CSF. Surgery is delayed on a daily basis if not met.

1. Hemoglobin ≧8 g/dL(Grade 0-2)
2. Neutrophil count ≧1,000/ mm^3^(Grade 0-2)
3. Platelet count ≧ 7.5×10^4^/ mm^3^ (Grade 0-1)
4. AST (GOT)≦100 IU/L
5. ALT (GPT)≦100 IU/L
6. Serum creatinine≦1.5 mg/dL
7. Infection Grade 0-1

### Criteria for additional resection (arm A & arm B)

If the initial surgery results in inadequate resection (positive histologic margins, intralesional excision, marginal excision, or wide resection for invasive tumors <1 cm margins), confirm that the most recent laboratory data within 7 days before the day of additional surgery have met 1) to 7) and perform additional resection. Transfusion-induced increases in hemoglobin are acceptable. Surgery for additional resection should be performed before the start of postoperative chemotherapy, after confirming adequate recovery of the patient's general condition within 28 days after surgery, using the day of previous surgery as day 0.

1. Hemoglobin ≧8 g/dL(Grade 0-2)
2. Neutrophil count ≧1,000/ mm^3^(Grade 0-2)
3. Platelet count ≧ 7.5×10^4^/ mm^3^ (Grade 0-1)
4. AST (GOT) ≦100 IU/L
5. ALT (GPT) ≦100 IU/L
6. Serum creatinine ≦1.5 mg/dL
7. Infection Grade 0-1

### Consultation on treatment modification

If there are any questions about treatment modification, contact "16.6. Study Coordinator".

Study Coordinator Contact: Kazuhiro Tanaka

Department of Orthopedics, Oita University Hospital

1-1 Idaigaoka, Hasama, Yufu City, Oita 879-5593, Japan

TEL: +81-97-586-5872

FAX: +81-97-586-6647

E-mail: ktanaka@oita-u.ac.jp

## Concomitant treatment and supportive care

### Required concomitant treatment/supportive care

#### 1) Laboratory Teste and Supportive Care for HBsAg-Negative and HBc Antibody-Positive and/or HBs Antibody-Positive Cases

HBV-DNA quantitative analysis should be performed at least once prior to initiation of chemotherapy. HBV-DNA assays are performed by real-time PCRs.

#### I) HBV-DNA ≥ 20 IU/mL (1. 3 log IU/mL) prior to initiation of chemotherapy

It has been clarified that HBV-DNA replicates persist at low levels in the livers and peripheral blood mononuclear cells when HBc or HBs are positive, even if they are HBs-Ag negative. It has been reported that reactivation of HBV and development of severe hepatitis are caused by the use of potent immunosuppressive agents even in such patients with previous infections.

If HBV-DNA ≥ 20 IU/mL (1.3 log IU/mL), the risk of HBV reactivation is judged to be as high as in HBsAg-positive cases, and prophylactic administration of nucleic acid analogues (entecavir, tenofovir disoproxil fumarate, tenofovir alafenamide fumarate) is administered. The following laboratory tests and supportive care are performed in accordance with the "Guideline for the Treatment of Hepatitis B, 3rd edition (Japanese Society of Hepatology)" with reference to the following for examination, dosage and dosing regimen of supportive therapy, and monitoring before the start of chemotherapy.

However, these are not applicable if the HBs antibody alone is positive and the HBV vaccination history is obvious.

##### i) Dosing schedule for supportive care (nucleic acid analogues prophylaxis)

- **Drugs used**
  - **Entecavir (Bristol-Myers: Baraclude Tablets 0.5 mg)**
  - **Tenofovir disoproxil fumarate (GlaxoSmithKline: Tenozet Tablets 300 mg)**
  - **Tenofovir alafenamide fumarate (Giliado: Vemuridi Tablets 25 mg)**

The following dosage regimen should be followed, starting at least 1 week before the start of chemotherapy (as soon as possible), and continuing for at least 12 months after the end of chemotherapy. After 12 months of completion of chemotherapy, nucleic acid analogues may be discontinued if the patient meets the conditions for discontinuing nucleic acid analogues. However, if the administration of a nucleic acid analogue is discontinued, consultation with a hepatologist is always obtained, and the administration is discontinued only if the hepatologist deems it appropriate. Bearing in mind that reactivation may occur after discontinuation of nucleic acid analogues treatment, HBV-DNA quantitative analysis should be continued at intervals specified in "ii) Monitoring." In addition, if HBV-DNA level is 20 IU/mL (1.3 log IU/mL) or more after discontinuation of nucleic acid analogues therapy, nucleic acid analogues therapy should be resumed immediately.

| - Requirements for discontinuation of nucleic acid analogues (entecavir, tenofovir disoproxil fumarate, tenofovir alafenamide fumarate): all of the following  1. The treatment has been continued for at least 12 months after the completion of immunosuppression or chemotherapy. 2. HBV-DNA quantitative analysis is persistently negative 3. HBsAg and HB core-related antigens are persistently negative 4. Normalized ALT (except for causes of ALT abnormalities other than HBV)   (Adapted from Guidelines for the Treatment of Hepatitis B, the 3rd edition (Japanese Society of Hepatology)) |
| --- |

##### Entecavir

- **Dosage regimen: Take this medicine on an empty stomach (2 hours after meals and more than 2 hours before the next meal).**
- **Dosage :**

| Creatinine clearance (mL/min). | Dosage |
| --- | --- |
| 50 or more | 0.5 mg once daily |
| ≥ 30, < 50 | 0.5 mg once every 2 days |
| ≥ 10, < 30 | 0.5 mg once every 3 days |
| < 10 | 0.5 mg once every 7 days |

- **Adverse drug reactions (incidence of all grades): nucleoside analog-naïve patients**

Diarrhea (6.0%), nausea (4.5%), constipation (3.7%), upper abdominal pain (3.0%), malaise (1.5%), nasopharyngitis (3.0%), headache (14.2%), dizziness (3.0%), rash (incidence unknown), laboratory tests: elevated AST (GOT) (3.7%), elevated ALT (GPT) (3.7%), increased blood bilirubin (6.0%), blood amylase increased (10.4%), lipase increased (6.0%), blood lactate increased (23.1%), BUN increased (6.7%), urine occult blood positive (4.5%), white blood cell count decreased (8.2%), eosinophil count increased (0.7%)

**[significant adverse reactions (incidence unknown)]** Hepatitis worsened after completion of treatment, anaphylactoid symptoms, lactic acidosis, severe hepatomegaly due to fatty liver

##### Tenofovir disoproxil fumarate

- **Dosage and administration: 300 mg is orally administered once daily.**
- **Dosage :**

| Creatinine clearance (mL/min). | Dosage |
| --- | --- |
| ≥ 50 | 300 mg once daily |
| ≥ 30, < 50 | 300 mg once every 2 days |
| ≥ 10, < 30 | 300 mg once every 3 to 4 days |
| Hemodialysis | 300 mg once every 7 days  Or 300 mg after completion of cumulative approximately 12 hours of dialysis  NOTE) After hemodialysis was performed. The pharmacokinetics in patients with creatinine clearance < 10 mL/min and not on hemodialysis have not been investigated. |

- **Dosing Precautions:**

In the long-term administration of tenofovir disoproxil fumarate, attention should be paid to renal dysfunction, hypophosphatemia (including Fanconi syndrome), and decrease in bone mineral density. It is recommended that renal function and serum phosphorus should be measured regularly during tenofovir disoproxil fumarate administration.

- **Adverse reactions (incidence of all grades):**

Seven patients (4.9%) had abnormal liver function tests (AST, ALT and γ-GTP increased, etc.), 4 patients (2.8%) had increased creatinine, 3 patients (2.1%) each had increased amylase, increased lipase and nausea, 2 patients (1.4%) each had abdominal pain

**[significant adverse reactions (incidence unknown)]** renal dysfunction, renal failure, acute renal failure, proximal renal tubular dysfunction, severe renal dysfunction such as Fanconi syndrome, acute renal tubular necrosis, renal diabetes insipidus or nephritis, severe hepatomegaly due to lactic acidosis and fatty deposition (steatohepatitis), pancreatitis

##### Tenofovir alafenamide fumarate

- **Dosage and administration: 25 mg is orally administered once daily.**
- **Dosage :**

| Creatinine clearance (mL/min). | Dosage |
| --- | --- |
| ≥ 15 | 25 mg once daily |
| < 15 | Consider discontinuation |

- **Dosing Precautions:**

In the long-term administration of tenofovir alafenamide fumarate, attention should be paid to renal dysfunction, hypophosphatemia (including Fanconi syndrome), and decrease in bone density. It is recommended that renal function and serum phosphorus should be measured periodically during tenofovir alafenamide fumarate administration.

- **Adverse reactions (incidence of all grades):**

Nausea and abdominal distension, headache, fatigue (≥ 1%), dyspepsia and diarrhea, flatus, upper abdominal pain, constipation, ALT increased, arthralgia, dizziness, insomnia, pruritus, rash (≥ 0.5% to <1%)

**[significant adverse reactions (incidence unknown)]** renal dysfunction, renal failure, acute renal failure, proximal renal tubular dysfunction, severe renal impairment such as Fanconi syndrome, acute renal tubular necrosis, renal diabetes insipidus or nephritis, severe hepatomegaly due to lactic acidosis and fatty deposits (fatty liver)

##### ii) Monitoring: Quantitative analysis of HBV-DNA (during and after administration of nucleic acid analogues)

**During nucleic acid analogue administration:**

They are monitored every 4 weeks by both HBV-DNA quantitative analysis and liver function (ASTs, ALTs). However, if HBV-DNA level is less than 20 IU/mL (1.3 log IU/mL) during administration of nucleic acid analogues, it is acceptable to perform tests every 4 to 12 weeks.

**After discontinuation of nucleic acid analogue administration:**

Bearing in mind that reactivation may occur even after discontinuation of administration of a nucleic acid analogues, the patient should be consulted with a hepatologist, and the patient should be monitored for HBV-DNA determination and hepatic function (AST/ALT) every 4 weeks for at least 1 year after discontinuation of administration of a nucleic acid analogues. If HBV-DNA level is 20 IU/mL (1.3 log IU/mL) or more after discontinuation of nucleic acid analogues therapy, the nucleic acid analogues therapy should be resumed immediately.

#### II) HBV-DNA < 20 IU/mL (1.3 log IU/mL) prior to initiation of chemotherapy

HBV-DNA quantitative analysis and hepatic function (AST, ALT) will be monitored, and nucleic acid analogues (entecavir, tenofovir disoproxil fumarate, tenofovir alafenamide fumarate) will be started when ≥ 20 IU/mL (1.3 log IU/mL) is achieved.

The Guidelines for the Treatment of Hepatitis B, the 3rd edition (Japanese Society of Hepatology) recommends monitoring with HBV-DNA quantitative analysis or high-sensitivity HBs antibodies during and after chemotherapy, depending on the risks of revitalization.

##### i) Monitoring: HBV-DNA quantitative analysis

HBV-DNA quantitative analysis should be performed every 4-12 weeks from the start of chemotherapy until at least 12 months after the end of chemotherapy.

If HBV-DNA level is more than 20 IU/mL (1.3 log IU/mL), administration of nucleic acid analogues should be started immediately in accordance with the Guidelines for the Treatment of Hepatitis B, the 3rd edition (Japanese Society of Hepatology). If HBsAg monitoring is positive for < 1 IU/mL (low positive), nucleic acid analogues should be administered after additional HBV DNA determinations of ≧ 20 IU/mL (1.3 log IU/mL). It is advisable to consult a hepatologist at a time prior to initiation of NAs.

##### ii) Supportive care in reactivation

Nucleic acid analogues should be administered according to the supportive care described in 6.4.1.1. i) HBV-DNA of 20 IU/mL (1.3 log IU/mL) or more prior to initiation of chemotherapy. Once administration of nucleic acid analogues is started, nucleic acid analogues should be discontinued only if appropriate by the hepatologist.

### Recommended/not recommended concomitant treatment/supportive care

The following concomitant treatment and supportive care are recommended. Even if it is not carried out, it is not regarded as protocol deviation,

#### 1) Addressing Fever During Neutropenia.

1. Assessment at onset of febrile neutropenia (FN).
2. If the neutrophil count is less than 500/mm^3^, or less than 1,000/mm^3^ and is predicted to decrease to less than 500/mm^3^ in less than 48 hours, and if the axillary temperature is 37.5°C (oral 38°C) or higher, then immediately assess the severity risks and start the antivirus treatment as appropriate.
3. Severity risk assessment is performed with reference to Multinational Association for Supportive Care in Cancer (MASCC) scoring system ^※1^.
4. For initial evaluation, complete blood cell count including differential and platelet count, renal function (BUN, creatinine), electrolytes, liver function (transaminases, total bilirubin, and alkaline phosphatase) tests, two or more sets of venous blood cultures before initiation of antimicrobials, one set of cultures from the catheter lumen and one set from a peripheral vein if a central venous catheter is in place, culture of suspected infected areas, and plain chest x-ray if respiratory symptoms or signs are present.
5. When febrile neutropenia (FN) develops in a patient with a central venous catheter, blood cultures from the catheter and peripheral blood are performed, and catheter-related infections are considered if there is a time difference of more than 120 minutes in the positivity of both. If appropriate antimicrobial therapy does not improve after more than 72 hours, catheter should be removed. For infections caused by Staphylococcus aureus, Pseudomonas aeruginosa, Bacillus, fungi, and acid-fast bacilli, the catheter should be removed and appropriate antimicrobial therapy based on culture results should be performed.
6. Antibiotic use
7. In high-risk patients, β-lactams with anti-Pseudomonas aeruginosa activity are administered intravenously as a single agent. However, other antimicrobials (aminoglycosides, fluoroquinolones, and/or vancomycin) may be added to a single agent in the initial regimen in patients with unstable or complicated conditions or when drug-resistant organisms are strongly suspected. Low-risk patients may be treated with antibiotics orally or intravenously, hospitalized, or with adequate evaluation, if appropriate, as outpatients.
8. The antimicrobial agent should be reassessed 3-4 days after initiation, and antimicrobial agents should be continued or changed. As a rule, antimicrobials should be continued until the neutrophil count is at least 500/mm^3^.
9. Empiric antifungal therapy is recommended in high-risk patients who do not respond to 4-7 days of broad-spectrum antibiotics.
10. Fluoroquinolone prophylaxis is recommended in high-risk patients with an expected neutrophil count ≤ 100 /mm^3^ lasting >7 days.
11. Therapeutic administration of G-CSF

Therapeutic administration of G-CSF during the development of FNs is refered to Section 6.4.5.3) Therapeutic Administration of G-CSF

*1 Multinational Association for Supportive Care in Cancer (MASCC) scoring system.

(Adapted in part from the Practice Guideline for Febrile Neutropenia (FN) [Japanese Society of Medical Oncology].*2)

| Item | Score |
| --- | --- |
| Clinical manifestations (select one of the followings)  * No symptoms  * Mild symptoms  * Moderate symptoms | 5  5  3 |
| No decrease in blood pressure | 5 |
| No chronic obstructive pulmonary disease | 4 |
| Solid tumors, or hematopoietic tumors without a history of fungal infection | 4 |
| No dehydration symptoms | 3 |
| Patients with fever during outpatient management | 3 |
| Age < 60 | 2 |

The total score is up to 26 points. Twenty-one points or more are considered low risk and 20 points or less are considered high risk

*2 Since patients aged 20 years or older are subjects in this study, we deleted "Not applicable to patients younger than 16 years old" from the original edition of the Practice Guideline for Febrile Neutropenia (FN) (Japanese Society of Medical Oncology).

#### 3) Nausea and vomiting

Antiemetics (5-HT3 receptor antagonists, metoclopramide, domperidone, steroids, aprepitant, palonosetron, etc.) should be used as needed, and fluid and electrolyte replacement should be if oral intakes are severely reduced^(44)^.

#### 4) Anemia

If Grade 3-4 anemia is observed (Hb < 8 g/dL), red blood cell transfusions are performed.

#### 5) Thrombocytopenia

If platelet count is 1.0×10^4^ /mm^3^ or less, or platelet count is 2.0×10^4^ /mm^3^ or less and there is a bleeding tendency, then the platelet transfusions are performed.

#### 6) Occult hematuria/hematuria

Urine pH should be kept above 7 during IFO administration (from day 1 to 24 hours after completion of IFO administration) in order to alkalinize urine. Urine pH should be examined whenever possible. If urine pH<7, approximately one ample of sodium bicarbonate (maylon) should be added to 1,000 mL of fluid during infusion. If occult hematuria is observed, administer adequate fluids. If occult hematuria appears during IFO administration, the mesna should be increased to the same daily dose of IFO. If hematuria is present, frequent urination and adequate fluid replacement should be encouraged to avoid retention of blood in the bladder. Bladder irrigation is performed if necessary.

#### 7) Diarrhea

Fluid and electrolyte supplements are given as needed. If Grade 3 or higher diarrhea develops, restrict feeding. Loperamide is not used in principle.

#### 8) Edema and allergic reactions

If necessary, antihistamines or steroids are administered.

#### 9) Shock symptoms and anaphylactoid symptoms

If it is judged to be due to the drug used for treatment, administration of the drug should be discontinued immediately, and appropriate measures such as administration of short-acting steroids, respiratory support, and administration of catecholamines should be taken.

#### 10) Interstitial pneumonitis

If pneumonitis is Grade 2 is higher, administration of the drug is discontinued and oxygen is administered. If rapid exacerbation occurs, appropriate measures such as steroid pulse therapy should be taken.

### Acceptable concomitant treatment and supportive care

The following concomitant treatment and supportive care may be used as needed.

- 1. Treatment of comorbidities such as hypertension and diabetes
  2. Symptomatic treatment for pain
  3. Oral antibiotics for prevention of febrile neutropenia

### Unacceptable concomitant treatment and supportive care

None of the following treatments is administered during the protocol treatment:

1. Administration of anticancer drugs other than protocol treatment
2. Hormone therapy other than steroids
3. Radiation therapy
4. Immune therapy

### Granulocyte colony-stimulating factor (G-CSF)

※ This study permits the use of G-CSF biogenerics (biosimilars).

#### 1) G-CSF primary precautionary administration ^※^

- - - - Primary prophylaxis: G-CSF administration before developing febrile neutropenia or prolonged neutropenia to prevent them during anticancer therapy.

Evidence on the risks of developing febrile neutropenia in arm A is scarce, and primary G-CSF prophylaxis should or should not be used. For this reason, the primary prophylactic administration of G-CSF is not specified in arm A of this study.

On the other hand, the risk of developing febrile neutropenia in arm B is 10-20% and having "Recent surgical treatment" as a risk factor that increases the frequency of febrile neutropenia. Therefore, in arm B, primary prophylactic administration of G-CSF is recommended in accordance with the "Guidelines for Appropriate Use of G-CSF, 2013 edition" and "Febrile Neutropenia (FN) Practice Guidelines by JSMO". However, no primary prophylaxis has been approved for the cancer types targeted in this study in any of the package inserts of filgrastim, naltograstim, or lenograstim. In addition, pegfilgrastim is approved for use in primary prophylaxis in all cancer types; however, since once-weekly administration of anticancer drugs is given in arm B, the primary prophylactic administration of pegfilgrastim is not recommended in this study based on the precautions for use in the package insert of pegfilgrastim (the safety of drug given 14 days prior to the start of cancer chemotherapy and 24 hours after the end of administration has not been established). For this reason, primary prophylaxis with G-CSF is not recommended in arm B of this trial.

Table 6.4.5. Primary prophylactic administration of G-CSF should be performed according to the approved dosage and administration shown in the table below.

| Drug | ・ Pegfilgrastim (arm A only)  ・ Filgrastim  ・ Naltograstim  ・ Lenograstim |
| --- | --- |
| Time of initiation | ・ Twenty-four hours after completion of chemotherapy |
| Dosage  Dosing regimen | ・ Pegfilgrastim (genetical recombination) administered subcutaneously at a dose of 3.6 mg/course of chemotherapy (arm A only)  ・ Filgrastim: 50 μg/m^2^ subcutaneously once daily or 100 μg/m^2^ intravenously once daily  ・ Naltograstim: 1 μg/kg SC once daily or 2μg/kg IV once daily  ・ Renograstim: 2 μg/kg SC once daily or 5μg/kg IV once daily |
| Timing of  discontinuation  (other than  pegfilgrastim) | ・ If neutrophil count reach a nadir level of at least 5,000/mm^3^ after the elapse, discontinue administration.  ・ If the neutrophil count recover to more than 2,000/mm^3^, if there are no symptoms suggestive of infection, and if the patient's safety is judged to be ensured, discontinue or reduce the dose of the drug. |

#### 2) Secondary prophylactic administration of G-CSF

- - - - Secondary prophylaxis: G-CSF prophylactic administration after once occurrence of febrile neutropenia or prolonged neutropenia to prevent febrile neutropenia or prolonged neutropenia from occurring again during anticancer therapy.

When febrile neutropenia occurs in the previous course in arm A, there is insufficient evidence to suggest whether secondary prophylaxis with G-CSF should be used, and secondary prophylaxis with G-CSF should or should not be used. Therefore, we do not decide whether to administer secondary prophylaxis of G-CSF in this study.

On the other hand, if febrile neutropenia occurs in the previous course in arm B, it is desirable to reduce the dose of anticancer drugs or modify the schedule according to the "the Guideline for Appropriate Use of G-CSF, 2013 edition" and "Febrile Neutropenia (FN) Practice Guidelines by JSMO". Therefore, secondary prophylaxis with G-CSF is not recommended in subsequent courses.

Since the safety of pegfilgrastim administered 14 days prior to the start of cancer chemotherapy and 24 hours after the end of administration has not been established, secondary prophylactic pegfilgrastim administration is not performed in arm B, which administers the drug in day 8.

Secondary prophylactic administration of G-CSF should be performed according to the approved dosage and administration shown in the table below.

| Drug | ・ Pegfilgrastim (A arm only)  ・ Filgrastim  ・ Naltograstim  ・ Lenograstim |
| --- | --- |
| Time of initiation | ・ Pegfilgrastim (arm A only)  Twenty-four hours after completion of chemotherapy  ・ Filgrastim, nartograstim, and lenograstim  When neutrophil count < 1,000/mm^3^ were observed |
| Dosage  Dosing regimen | ・ Pegfilgrastim (genetical recombination) administered subcutaneously at a dose of 3.6 mg/course of chemotherapy (arm A only)  ・ Filgrastim: 50 μg/m^2^ subcutaneously once daily or 100 μg/m^2^ intravenously once daily  ・ Naltograstim: 1 μg/kg SC once daily or 2μg/kg IV once daily  ・ Renograstim: 2 μg/kg SC once daily or 5μg/kg IV once daily |
| Timing of  discontinuation  (other than  pegfilgrastim) | ・ If neutrophil count reach a nadir level of at least 5,000/mm^3^ after the elapse, discontinue administration.  ・ If the neutrophil count recover to ≧2,000/mm^3^, if there are no symptoms suspicious of infection, and if the patient's safety is determined to be ensured, discontinue or reduce the dose of the drug. |

#### 3) Therapeutic administration of G-CSF

Therapeutic administration of G-CSF should be performed according to the approved dosage and administration shown in the table below.

| Time of initiation | ・ When neutrophil count are below 1,000/mm^3^ and fever (38°C or higher as a general rule) occurs  ・ When neutrophil count < 500/mm^3^ were observed |
| --- | --- |
| Dosage  Dosing regimen | ・ Filgrastim: 50 μg/m^2^ subcutaneously once daily or 100 μg/m^2^ intravenously once daily  ・ Naltograstim: 1 μg/kg SC once daily or 2μg/kg IV once daily  ・ Renograstim: 2 μg/kg SC once daily or 5μg/kg IV once daily |
| Timing of  discontinuation | ・ If neutrophil count reach a nadir level of at least 5,000/mm^3^ after the elapse, discontinue administration.  ・ If the neutrophil count recover to ≧2,000/mm^3^, if there are no symptoms suspicious of infection, and if the patient's safety is determined to be ensured, discontinue or reduce the dose of the drug. |

## Post-study treatment

After protocol treatment completion, patients should be observed without treatment until progression or relapse is observed.

Treatment after termination of protocol treatment and treatment after progression or relapse after completion are not specified. However, chemotherapy with the same treatment regimen as the protocol treatment (regimens containing both ADM and IFO in arm A and both GEM and DOC in arm B) is not acceptable.

Treatment with the drugs included in the opposite arm treatment regimen (cross over) is allowed.

If the primary conclusions of the study are determined by primary analysis or interim analysis, the results of the study should be explained to patients enrolled in the study as appropriate, and the best available treatment should be provided with the consideration of the treatment course of the individual patients.

In addition, if the criteria for termination of protocol treatment are met, but continuation of protocol treatment is clinically considered appropriate, consult the Study Coordinator through the Investigator rather than deciding on it at the Sub-investigator level in principle (except when time is not available). Under the agreement of the Study Coordinator and the Site Investigator/Site Coordinator, decide whether to treat as a treatment after termination of the protocol treatment or to continue the protocol treatment with protocol deviation. The details of the consultation with the Study Coordinator and the history of the decision making should be provided in the comments section of the patient's Off-treatment Form and Treatment Form. If continuing the protocol treatment with protocol deviation occurs frequently, the Study Coordinator should consider revising protocol treatment termination criteria using group meetings and group mailing lists, because it is considered that the protocol treatment termination criteria is clinically inappropriate in such situation.

### Recommended post-study treatment: Radiotherapy

We recommend that radiotherapy be administered as post-study treatment after protocol treatment if the surgery ends with inadequate resection (positive histological margin, intralesional excision, marginal excision, or wide excision for invasive tumors with less than 1 cm margins) and no additional surgery is performed, or if additional surgery resulted in inadequate resection.

Methods of radiotherapy are recommended as the following.

**1) Dose and fractionation**

Total dose: 50-70 Gy (basically 60 Gy)

Single dose is 2 Gy once a day, 5 times a week in the principle.

Irradiation direction, irradiation portal number are not specified. The total duration of treatment and the allowable total duration of treatment are not specified.

**2) Radiotherapy equipment**

A 4-10 MV X-ray generator with a Source Axis Distance (SAD) of 100 cm or more is used. It is desirable to use a 10 MV generator for the trunk.

**3) Approximate maximum dose for organs at risk (dose calculated by dose distribution rather than prescription dose)**

Spinal cord: 46 Gy Lung: V20 < 20%

Esophagus: 55 Gy Stomach, colon: 50 Gy

Small intestine, duodenum: 45 Gy Rectum: 60 Gy

# Examination and Evaluation

## Baseline examination and evaluation before registration

### Examination and evaluation before registration (irrespective of timing before registration)

- - 1. HBs antigen, HBc antibody^※^, HBs antibody^※^

※ If at least one of the HBc and HBs antibodies is positive, HBV-DNA should also be measured prior to initiation of therapy (see 6.4.1).

- - 1. Tumor incisional biopsy (a minimum of 10 unstained specimens can be prepared or prepared. In cases of local recurrence, submission of pathological specimens at the initial stage is mandatory, but eligible even if 10 unstained specimens cannot be secured)

The following tests should be performed before registration whenever possible.

- Whole-body bone scintigraphy (Tc99m-HMDP) (FDG-PET when difficult).

### Examination and evaluation performed within 28 days before registration

1. Regional MRI (slice thickness ≦ 5 mm. T1-weighted/T2-weighted in transverse view is mandatory. T1 contrast is performed whenever possible. If contrast media allergy is present, it is difficult to use contrast media. Simple MRI is acceptable.
2. CT or MRI of regional lymph node area (slice thickness ≦5 mm, with or without contrast)
3. Chest computed tomography (slices ≦5 mm in thickness, with or without contrast) (PET-CT images are acceptable)
4. 12-lead, resting electrocardiography

### Examination and evaluation performed within 14 days before registration

1. General condition: PS (ECOG), body weight
2. Peripheral blood count: white blood cell count, neutrophil count (ANCs: rod-shaped nuclear cells + segmented nuclear cells), hemoglobin, platelets
3. Blood chemistry: albumin, total bilirubin, AST (GOT), ALT (GPT), BUN, creatinine, LDH, calcium, sodium, potassium, CRP, FBS (fasting blood glucose)
4. Urinalysis (casual urine): urine occult blood, urine glucose qualitative, urine protein qualitative
5. Creatinine clearance (estimated by Cockcroft-Gault equation)
6. Cutaneous oxygen saturation (SpO_2_)
7. Chest radiograph (2 directions)

## Examination and evaluation during treatment

The following safety examinations and evaluations are minimal in frequency: Performing examinations more frequently at the discretion of the treating physician is not prohibited. However, the examination for efficacy evaluation should be performed at specified frequencies, unless progression is suspected, because dense frequency may lead to bias in the efficacy evaluation.

### Safety examinations and evaluations assessed weekly (written in CTCAE v4.0-JCOG) (common in preoperative and postoperative)

1. PS
2. Subjective and objective findings (described according to CTCAE v4.0-JCOG)
   - - - General disorders and administration site conditions: fever, edema limbs, fatigue
       - Blood and lymphatic system disorders: febrile neutropenia
       - Skin and subcutaneous tissue disorders: palmar-plantar erythrodysesthesia syndrome, skin hyperpigmentation
       - Gastrointestinal disorders: diarrhea, nausea, oral mucositis
       - Metabolism and nutrition disorders: anorexia
       - Nervous system disorders: dizziness (vertigo, floating), leukoencephalopathy, peripheral sensory/motor neuropathy, decreased level of consciousness, seizure
       - Infections and infestations: bronchial infection, lung infection, soft tissue infection, bone infection
       - Cardiac disorders: supraventricular tachycardia, ventricular arrhythmias, left ventricular systolic dysfunction (Grade 0 unless subjective symptoms or arrhythmias are present, and ECG is not mandatory)
       - Respiratory, thoracic and mediastinal disorders: pneumonitis
       - Renal and urinary disorders: hematuria
3. Peripheral blood count: hemoglobin, white blood cell count, platelet count, neutrophil count (rod cell count + segmented cell count)
4. Biochemical tests: total bilirubin, ALP, AST (GOT), ALT (GPT), BUN, creatinine, sodium, potassium
5. Chest radiograph (2 directions)*
6. Cutaneous oxygen saturation(SpO_2_)*

* It should be performed only at the time of Grade 1 pneumonitis.

### Safety examinations and evaluations assessed every course (within 3 days of course initiation) during chemotherapy (common in preoperative and postoperative)

1. Items in "8.2.1. Safety examinations and evaluations assessed at least once a week"
2. General condition: body weight
3. Biochemical tests: albumin, LDH, calcium, CRP
4. Urinalysis (casual urine): urine occult blood
5. Chest radiograph (2 directions)

### Safety examinations and evaluations to be performed as needed

1. If dyspnea or Grade 1 pneumonitis is present
   - Cutaneous oxygen saturation(SpO_2_)
   - Arterial Oxygen-Saturation: PaO_2_
   - Chest radiograph (2 directions)
2. If an arrhythmia is observed
   - 12-lead, resting electrocardiography
3. When symptoms of heart failure are observed
   - Echocardiography

### Examinations for efficacy evaluation (response evaluation after preoperative chemotherapy)

After completion of preoperative chemotherapy, the following examinations are performed 14 days or less than 35 days after day1 of the 3rd course. In accordance with "11.1. Response evaluation", perform response evaluation, and assess whether the tumor is resectable or not, and determine the surgical method.

1. Chest CT (slice thickness ≦5 mm, with or without contrast enhancement).
2. Regional MRI: T1-weighted, T2-weighted, (slice thickness ≦5 mm, T1 contrast enhanced and plain MRI are acceptable. Same imaging methods are used as pretreatment baseline evaluation)

If preoperative chemotherapy is discontinued, perform 1) and 2) above as much as possible after 14 days and 35 days after the first day of the final preoperative chemotherapy course, and perform response evaluation, whenever feasible. When the regional MRI is performed, the decision of whether it is resectable or not and the decision of the surgical method are made by those MRI images.

### Examinations and evaluations before surgery (including additional resections)

#### 1) Examinations and evaluations before the first surgery

The followings are evaluated within 14 days before surgery:

- 1. Chest radiograph (2 directions)
  2. 12-lead, resting electrocardiography
  3. Pulmonary function test

The followings are evaluated with the most recent laboratory values within 7 days before the day of surgery:

- 1. General condition: PS (ECOG), body weight
  2. Peripheral blood count: white blood cell count, neutrophil count (segmented nuclear cell + rod nuclear cell), hemoglobin, platelet count
  3. Blood chemistry: total protein, albumin, total bilirubin, AST (GOT), ALT (GPT), BUN, creatinine, LDH, calcium, sodium, potassium, CRP

#### 2) Examinations and evaluations before additional resection

The followings are evaluated with the most recent laboratory values within 7 days before the day of surgery:

1. General condition: PS (ECOG), body weight
2. Peripheral blood count: white blood cell count, neutrophil count (segmented nuclear cell + rod nuclear cell), hemoglobin, platelet count
3. Blood chemistry: total protein, albumin, total bilirubin, AST (GOT), ALT (GPT), BUN, creatinine, LDH, calcium, sodium, potassium, CRP

### Examinations and evaluations associated with surgery

The followings should be recorded (assessed for each of the first surgery and additional surgery in the case with additional surgery):

1. operative time, intraoperative blood loss
2. Surgical procedures: limb preservation, amputation, and others (describe in details)
3. Complicated resection tissue: Specific description of resection of nerves, blood vessels, bones, etc.
4. Reconstructive procedures (if reconstruction is performed): Specific description of skin grafts, myocutaneous flaps, prostheses, revascularization, etc.
5. Margin evaluation: wide margin (shortest margin (cm) (shortest longitudinal, shortest transverse)), marginal margin, intralesional margin
6. Postoperative blood loss, transfusion volume

### Postoperative pathological diagnosis

The followings are assessed (for additional surgical cases, surgical procedures of both the initial surgery and additional surgery are evaluated):

1. Histological type diagnosis of the resected specimen
2. Evaluation of surgical margins: positive, negative, or inconclusive
3. Pathological response evaluation of resected lesions: Grade 1/2/3/4/NE
4. Percentage of residual tumor

### Intraoperative and postoperative safety evaluation

The followings are evaluated:

#### 1) Intraoperative complications

Subjective and objective findings (described according to CTCAE v4.0-JCOG)

- - - - Injury, poisoning and procedural complications: intraoperative hemorrhage, intraoperative arterial injury, intraoperative venous injury, intraoperative neurological injury

#### 2) Early postoperative complication (within 27 days after surgery (including additional resection) or just before starting postoperative chemotherapy)

Only the first operation should be performed within 27 days after surgery, and if additional surgery is performed, it should be performed between the first operation and 27 days after additional surgery.

Subjective and objective findings (described according to CTCAE v4.0-JCOG)

- - - - General disorders and administration site conditions: fever, edema limbs
      - Vascular disorders: peripheral ischemia, thromboembolic event
      - Injury, poisoning and procedural complications: wound dehiscence
      - Nervous system disorders: Nervous system disorders
      - Infections and infestations: bone infection, joint infection, lung infection, bronchial infection, wound infection, soft tissue infection

## Examination and evaluation after the end of treatment

### Examination and evaluation after completion/termination of protocol treatment

After protocol treatment completion or termination, the followings are examined or evaluated in the frequencies specified below until the end of follow-up period.

Once every 3 months: chest radiograph (2 directions) (chest radiograph can be omitted if chest CT is taken)

Once every 6 months: chest CT

Once a year: evaluations of late complications

- - - - General disorders and administration site conditions: edema limbs
      - Cardiac disorders: supraventricular tachycardia, ventricular arrhythmias (Grade 0 if there are no subjective symptoms or arrhythmias, ECG is not mandatory)
      - Respiratory, thoracic and mediastinal disorders: dyspnea, pneumonitis
      - Nervous system disorders: peripheral sensory/motor neuropathy
      - Vascular disorders: peripheral ischemia
      - Peripheral blood count: hemoglobin, white blood cell count, platelet count, neutrophil count (rod cell count + segmented cell count)
      - Biochemical tests: total bilirubin, ALP, AST (GOT), ALT (GPT), BUN, creatinine, sodium, potassium, total protein, albumin, LDH, calcium, CRP
      - Urinalysis (casual urine): urine occult blood, urine glucose qualitative, urine protein qualitative

### Radiotherapy and post-study treatment Information after relapse

Radiotherapy as post-study treatment and post-study treatment after relapse are documented on Follow-up Forms at each follow-up survey.

1. PS at the start of post-study treatment (PS should be documented in the medical record)
2. Radiotherapy or not (irradiation site, total dose, if performed)
3. Site of relapse, the number of metastatic lesions at the site of relapse, and the number of relapsed organs
4. Post-study treatment after relapse
5. Secondary cancer

## Study calendar

|  | Before preoperative chemotherapy | Preoperative chemotherapy | | | | | | | | | Preoperative  After chemotherapy  Day 14  From  35 days | Before surgery (additional resection) | Intraoperative complications | Early postoperative complications |
| --- | --- | --- | --- | --- | --- | --- | --- | --- | --- | --- | --- | --- | --- | --- |
| Course |  | 1 | | | 2 | | | 3 | | |  |  |  |  |
| Week |  | 1 | 2 | 3 | 4 | 5 | 6 | 7 | 8 | 9 |  |  |  |  |
| General condition |  |  |  |  |  |  |  |  |  |  |  |  |  |  |
| Physical findings | ○ | ○ | ○ | ○ | ○ | ○ | ○ | ○ | ○ | ○ |  |  |  |  |
| Body weight | ○^14^ | ○ |  |  | ○ |  |  | ○ |  |  |  | ●^7^ |  |  |
| PS | ○^14^ | ○ | ○ | ○ | ○ | ○ | ○ | ○ | ○ | ○ |  | ●^7^ |  |  |
| Incisional tumor biopsy | ○^Pre^ |  |  |  |  |  |  |  |  |  |  |  |  |  |
| Laboratory tests |  |  |  |  |  |  |  |  |  |  |  |  |  |  |
| CBC | ○^14^ | ○ | ○ | ○ | ○ | ○ | ○ | ○ | ○ | ○ |  | ●^7^ |  |  |
| T-Bil、ALP、AST、ALT、  BUN、Cr、Na、K | ○^14^ | ○ | ○ | ○ | ○ | ○ | ○ | ○ | ○ | ○ |  | ●^7^ |  |  |
| Alb、LDH、Ca、CRP | ○^14^ | ○ |  |  | ○ |  |  | ○ |  |  |  | ●^7^ |  |  |
| FBS (fasting blood glucose) | ○^14^ |  |  |  |  |  |  |  |  |  |  |  |  |  |
| CCr | ○^14^ |  |  |  |  |  |  |  |  |  |  |  |  |  |
| Urinalysis | ○^14^ | ○ |  |  | ○ |  |  | ○ |  |  |  |  |  |  |
| HBs-Ag, HBs-Ab,  HBc-Ab, HCV-Ab | ○^Pre^ |  |  |  |  |  |  |  |  |  |  |  |  |  |
| Radiological examination (response evaluation) |  |  |  |  |  |  |  |  |  |  |  |  |  |  |
| Chest radiograph  (2 directions) | ○^14^ | ○ |  |  | ○ |  |  | ○ |  |  |  | ●^14^ |  |  |
| Chest CT/MRI | ○^28^ |  |  |  |  |  |  |  |  |  | ○ |  |  |  |
| Regional MRI | ○^28^ |  |  |  |  |  |  |  |  |  | ○ |  |  |  |
| Whole-body bone  scintigraphy | ○^28^ |  |  |  |  |  |  |  |  |  |  |  |  |  |
| 12-lead, resting  electrocardiography | ○^28^ |  |  |  |  |  |  |  |  |  |  | ●^14^ |  |  |
| Pulmonary function test |  |  |  |  |  |  |  |  |  |  |  | ●^14^ |  |  |
| Cutaneous oxygen  saturation(SpO2) | ○^14^ |  |  |  |  |  |  |  |  |  |  |  |  |  |
| Toxicity evaluation |  |  |  |  |  |  |  |  |  |  |  |  |  |  |
| Subjective/objective  findings | ○ | ○ | ○ | ○ | ○ | ○ | ○ | ○ | ○ | ○ |  |  |  |  |
| Intraoperative complications |  |  |  |  |  |  |  |  |  |  |  |  | ○ |  |
| Early postoperative  complications |  |  |  |  |  |  |  |  |  |  |  |  |  | ○ |
| Late complications |  |  |  |  |  |  |  |  |  |  |  |  |  |  |
| Submission of CRFs |  |  |  |  |  |  |  |  |  |  |  |  |  |  |
| On-preoperative  Treatment Form | □ |  |  |  |  |  |  |  |  |  |  |  |  |  |
| Preoperative Treatment  Form |  | □ | □ | □ | □ | □ | □ | □ | □ | □ |  |  |  |  |
| Off-preoperative  Treatment Form |  |  |  |  |  |  |  |  |  |  | □ |  |  |  |
| Response Evaluation  Form |  |  |  |  |  |  |  |  |  |  | □ |  |  |  |
| Preoperative Form |  |  |  |  |  |  |  |  |  |  | □ |  |  |  |
| (Additional) Surgery  Form |  |  |  |  |  |  |  |  |  |  | □ |  |  |  |
| Postoperative Form |  |  |  |  |  |  |  |  |  |  |  |  |  | □ |
| Pathology Form |  |  |  |  |  |  |  |  |  |  |  |  |  | □ |

○: Implement, ○^Pre^: Perform until registration, ○^28^: Perform within 28 days before registration, ○^14^: Perform within 14 days before registration,

●^7^ Perform within 7 days before surgery, ●^14^: Perform within 14 days before the first surgery, □: Submit

|  | Before postoperative chemotherapy | Postoperative chemotherapy | | | | | | After protocol treatment /termination to the end of follow-up period |
| --- | --- | --- | --- | --- | --- | --- | --- | --- |
| Course |  | 1 | | | 2 | | |  |
| Week |  | 1 | 2 | 3 | 4 | 5 | 6 |  |
| General condition |  |  |  |  |  |  |  |  |
| Physical findings | ○ |  | ○ | ○ | ○ | ○ | ○ |  |
| Body weight | ○ |  |  |  | ○ |  |  |  |
| PS | ○ |  | ○ | ○ | ○ | ○ | ○ |  |
| Incisional tumor biopsy |  |  |  |  |  |  |  |  |
| Laboratory tests |  |  |  |  |  |  |  |  |
| CBC | ○ |  | ○ | ○ | ○ | ○ | ○ | △ |
| T-Bil、ALP、AST、ALT、BUN、Cr、Na、  K | ○ |  | ○ | ○ | ○ | ○ | ○ | △ |
| Alb、LDH、Ca、CRP | ○ |  |  |  | ○ |  |  | △ |
| FBS (fasting blood glucose) |  |  |  |  |  |  |  |  |
| CCr |  |  |  |  |  |  |  |  |
| Urinalysis | ○ |  |  |  | ○ |  |  | △ |
| HBs-Ag, HBs-Ab, HBc-Ab, HCV-Ab |  |  |  |  |  |  |  |  |
| Radiological examination (response evaluation) |  |  |  |  |  |  |  |  |
| Chest radiograph (2 directions) | ○ |  |  |  | ○ |  |  | ● |
| Chest CT/MRI |  |  |  |  |  |  |  | ◎ |
| Regional MRI |  |  |  |  |  |  |  |  |
| Whole-body bone scintigraphy |  |  |  |  |  |  |  |  |
| 12-lead, resting electrocardiography |  |  |  |  |  |  |  |  |
| Pulmonary function test |  |  |  |  |  |  |  |  |
| Cutaneous oxygen saturation(SpO2) |  |  |  |  |  |  |  |  |
| Toxicity evaluation |  |  |  |  |  |  |  |  |
| Subjective/objective findings | ○ |  | ○ | ○ | ○ | ○ | ○ | △ |
| Intraoperative complications |  |  |  |  |  |  |  |  |
| Early postoperative complications |  |  |  |  |  |  |  |  |
| Late complications |  |  |  |  |  |  |  | △ |
| Submission of CRFs |  |  |  |  |  |  |  |  |
| On-postoperative Treatment Form | □ |  |  |  |  |  |  |  |
| Postoperative Treatment Form |  | □ | □ | □ | □ | □ | □ |  |
| Off-postoperative Treatment Form |  |  |  |  |  |  |  | □ |
| Off-treatment Form |  |  |  |  |  |  |  | □ |
| Follow-up Form |  |  |  |  |  |  |  | 2 times/year |

○: Implementation; ●: once every 3 months; ◎: once every 6 months; △: once every 1 year

□: Submit

※ Follow-up Forms will be sent up to 5 years after completion of accrual and will be submitted after 5 years of registration in the individual patient according to the closing date of registration.

# Response Evaluation and Endpoint Definition

## Response evaluation

Tumor response evaluation is performed according to the following instructions in compliance with the Japanese JCOG version of "New response evaluation criteria in solid tumours: Revised RECIST guideline (version 1.1)"^(45)^. The original article of RECIST version 1.0 stipulated in the Introduction that "use in the context of decisions regarding continuation of therapy is not the primary focus of this document." and a similar description is seen also in RECIST version 1.1 as follows:

"many oncologists in their daily clinical practice follow their patients’ malignant disease by means of repeated imaging studies and make decisions about continued therapy on the basis of both objective and symptomatic criteria. It is not intended that these RECIST guidelines play a role in that decision making, except if determined appropriate by the treating oncologist."

Therefore, the "overall response" as determined by response evaluation in accordance with the RECIST guidelines should be used to determine whether a drug or treatment regimen shows promising study results worthy for continuing research and development of them. In other words, the judgment of whether or not to continue treatment in individual patients should not be based on CR/PR/SD/PD of the overall response, but rather on the clinical judgment, which is based on a comprehensive consideration of symptoms, physical findings, and various laboratory data as well as imaging findings.

Therefore, there are cases where it is clinically appropriate to continue the protocol treatment even when it is judged as "PD (Progressive Disease)" as the overall response by response evaluation based on the imaging diagnosis. However, although whether or not to continue protocol treatment should be decided by clinical judgment regardless of response evaluation in those cases, the event date of progression-free survival should be the date on which the overall response is judged to be PD. This is due to three reasons: (i) the decision whether or not to continue protocol treatment for each arm may differ; (ii) the RECIST guidelines are the criteria which intend to standardize progression-free survival as well as response proportion (response rate); and (iii) the standard definition in the US Cooperative Groups is that the event date of progression-free survival is defined as the date on which the overall response is judged to be PD in any situation.

On the other hand, if the patient does not meet the criteria for "PD" according to response evaluation criteria based on imaging, but the treating physician judges "clinical progression" based on clinical and comprehensive judgment not based on imaging, protocol treatment should be terminated according to "6.2.2. Protocol treatment termination criteria". If the patient is judged as "clinical progression", the event date of progression-free survival should be the date on which judged as "clinical progression", even if not judged as "PD" by response evaluation. This is because imaging after a patient is judged to have "clinical progression" is often not done on schedule, so if "clinical progression" is not an event for progression-free survival, progression-free survival is likely to be overestimated. It should be noted that treating "clinical progression" as "censoring" in progression-free survival is also statistically incorrect (informative censoring) because it would handle the patients at increased risk of progression or death as censored cases.

In the original article of RECIST v1.1, it is described that "unequivocal progression" in the PD criteria for non-target lesions is "an overall level of substantial worsening in non-target disease such that the overall tumour burden has increased sufficiently to merit discontinuation of therapy," and therefore the judgement of PD in non-target lesions includes "judgment of whether or not to continue treatment in individual patients". It is confusing. It should be noted that this "unequivocal progression" is only a criterion for "PD in non-target lesions."

The relationships among 'PD', 'clinical progression', 'progression', and the events of progression-free survivals are as shown in Figure 11.1. in JCOG.


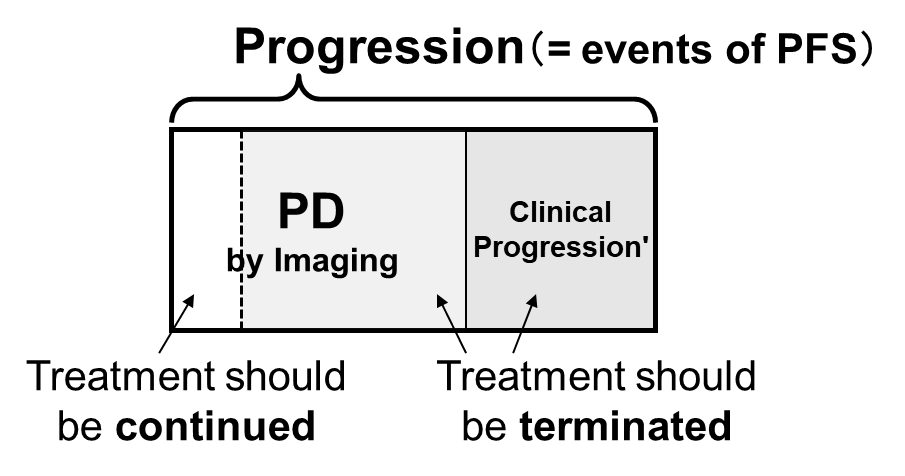


Figure 11.1. Relationship among Progression, PD by Imaging, and Clinical Progression.

### Baseline Evaluation

According to "8.1. Baseline examination and evaluation before registration", tumorous lesions at baseline are identified and categorized into "measurable lesions" and "non-measurable lesions" by chest CT (slice thickness ≦5 mm), regional MRI (slice thickness ≦5 mm), and CT or MRI including regional lymph node sites before registration.

Measurement of tumor diameter is carried out on the axial plane of MRI, and the measurement on 3-dimensional reconstruction image of CT and sagittal or coronal plane of MRI are not used. Baseline evaluations are done using the most recent imaging within 28 days prior to registration. If the other imaging studies are performed after registration and before initiation of protocol treatment, the most recent imaging studies should be used.

### Definition of measurable lesions

A measurable lesion is defined as a lesion that meets any of the following: In this study, lesions other than malignant lymph nodes (tumor lesions) are assessed by MRI, while malignant lymph nodes are assessed by MRI or CT.

- 1. Tumor lesions (non-nodal lesions) : lesions with minimum size of 10 mm in longest diameter other than malignant lymph nodes on MRI of 5 mm or less in slice thickness
  2. Malignant lymph nodes (nodal lesions): lymph nodes ≧15 mm in short axis on MRI or CT with slice thickness ≦5 mm

(Lymph nodes with ≧10 to < 15 mm in short axis are considered non-target lesions, and those with < 10 mm in short axis are not considered as malignant lymph nodes.)

All other lesions are considered non-measurable lesions.

### Selection of target lesions and baseline documentation

Up to a maximum of five measurable lesions at baseline (a maximum of to two lesions per organ), in descending order of diameter (longest diameter for non-nodal lesions and short axis diameter for nodal lesions) should be identified as target lesions. Target lesions should be selected on the basis of their size (lesions with the longest diameter), representative of all involved organs, but in adition should be those that lend themselves to reproducible repeated measurements (avoiding lesions that are difficult to measure even if they are large in diameter).

For selected target lesions, the region (code), examination methods, date of examination, longest diameter of non-nodal lesions, short axis diameter of nodal lesions, and the sum of the diameters of all target lesions (hereinafter referred to as the sum of diameters) are reported from head to tail in Pre-treatment Form 3.

### Baseline documentation of non-target lesions

All other lesions which are not selected as target lesions should be identified as non-target lesions regardless of whether or not they are measurable, and those site (code), examination methods, and date of examination are reported in Pre-treatment Form 3. Multiple non-target lesions in the same organ may be recorded as a single lesion (e.g., multiple enlarged pelvic lymph nodes, multiple liver metastases).

### Determining objective tumor response

Evaluation of the target lesions and non-target lesions are performed according to "8.2 Examination and evaluation during treatment" within 14 days and 35 days after day1 of the 3rd course of preoperative chemotherapy using the same examination methods as at baseline, and the diameters of the target lesions and the disappearance or progression of the non-target lesions should be recorded in the Response Evaluation Form.

### Response evaluation criteria for target lesions

**・ Complete Response (CR):**

Disappearance of all target lesions. Any pathological lymph nodes (whether target or non-target) must have reduction in short axis to<10 mm. When lymph nodes are included as target lesions, the ‘sum’ of lesions may not be zero even if complete response criteria are met

**・ Partial Response (PR):**

At least a 30% decrease in the sum of diameters of target lesions, taking as reference the baseline sum of diameters.

**・ Progressive Disease(PD):**

At least a 20% increase in the sum of diameters of target lesions, taking as reference the smallest sum on study (this includes the baseline sum if that is the smallest on study). In addition to the relative increase of 20%, the sum must also demonstrate an absolute increase of at least 5 mm.

**・ Stable Disease (SD):**

Neither sufficient shrinkage to qualify for PR nor sufficient increase to qualify for PD, taking as reference the smallest sum of diameters while on study.

**・ Not all Evaluated (NE):**

If some of examinations could not be performed for any reason or if neither CR, PR, PD, or SD could be determined.

Pre-treatment sum of diameters - sum of diameters at the time of study

%Decrease of sum of diameters = ------------------------------------------------------------------------------------------------ x 100%

Pre-treatment sum of diameters

Sum of diameters at examination - minimum sum of diameters

%Increase of sum of diameters = ------------------------------------------------------------------------------------------------ x 100%

Minimum sum of diameters

- The diameters of the target lesions should be measured, and their actual measurements should be recorded whenever measurable (e.g., <5 mm). If the diameter of the target lesion is judged to be 'too small to measure' and it is the opinion of the radiologist that the lesion has likely disappeared, the measurement should be recorded as 0 mm. If the lesion is believed to be present and is faintly seen but too small to measure, a default value of 5 mm should be assigned, regardless of the CT slice thickness.
- If %Decrease meets the criteria of PD and %Increase meets the criteria of PR simultaneously, the tumor response should be PD.
- When non-nodal lesions ‘fragment’, the longest diameters of the fragmented portions should be added together to calculate the target lesion sum.
- If the lesions have truly coalesced such that they are no longer separable, the vector of the longest diameter in this instance should be the maximal longest diameter for the ‘coalesced lesion’. If lesions coalesce, a plane between them may be maintained that would aid in obtaining maximal diameter measurements of each individual lesion.

### Response evaluation criteria for non-target lesions

**・ Complete Response (CR):**

Disappearance of all non-target lesions and normalisation of tumor marker level. All lymph nodes must be non-pathological in size(<10mm short axis).

**・ Non-CR/Non-PD:**

Persistence of one or more non-target lesion(s) and/or maintenance of tumor marker level above the normal limits.

**・ Progressive Disease (PD):**

Unequivocal progression of existing non-target lesions.

When the patient also has measurable disease. In this setting, to achieve ‘unequivocal progression’ on the basis of the non-target lesions, there must be an overall level of substantial worsening in non-target lesions such that, even in presence of SD or PR in target lesions, the overall tumor burden has increased sufficiently to merit discontinuation of therapy. If the response of the target lesions is SD or PR, the increase in tumor burden of the non-target lesions to a much greater extent than the decrease in tumor burden in the target lesions is considered as ‘unequivocal progression’ and if not, response of the non-target lesions should be Non-CR/Non-PD.

Having only non-measurable lesions: An ‘unequivocal progression’ is defined as an increase in non-target lesions that is judged to clearly exceed the tumor burden corresponding to a 20% increase in diameter and a 73% increase in tumor volume.

**・ Not all Evaluated (NE):**

If some examinations could not be performed for any reason or if neither CR, Non-CR/Non-PD, nor PD could be determined

### New lesions

If a lesion that was not present at baseline was present after initiation of treatment, it should be considered as "new lesion".

However, a "new lesion" must not be a change in the image due to a difference in the imaging methods or a change in the imaging modality from the examination at the baseline evaluation, or a change in the imaging findings thought to representing something other than the tumor. For example, a cystic lesion arising within a lesion due to necrosis of a liver metastatic lesion is not considered a new lesion. New lesions are defined as new lesions by examination of sites that were not mandatory at baseline (evaluation before registration).

If a lesion disappears and later reappears, measurements should be continued. However, the response at the time the lesion reappears depends on the status of the other lesions. If the lesions reappear after CR, it is judged as PD at the time of reappearance. On the other hand, if the overall response is PR or SD, the once disappeared lesion reappears, the diameter of the lesion should be added to the sum of diameters of the remaining lesions to evaluate response. That is, in the condition where many lesions remain, even if a lesion reappears after an disappearance in imaging only, it is not considered to be PD, and it is considered to be PD when the sum of the diameters of all target lesions meets the criteria for PD. This is due to the realization that most lesions do not truly 'disappear' and are not only depicted due to the limitation of resolution of the imaging modalities used.

If a new lesion is suspected but not definitive, it should not be a new lesion, and imaging studies should be repeated at a clinically relevant time interval. When a new lesion is confirmed by repeated imaging, it is judged as the new lesion at the date of imaging at which the new lesion is confirmed.

### Overall Response

The overall response are determined by combining the response of the target lesions, the response of the non-target lesions, and the presence or absence of new lesions within 14 days and 35 days after day1 of the 3rd course of preoperative chemotherapy according to Table 11.1.9.a below. The overall response in patients without non-target lesions is determined by combining the response of the target lesions and the presence or absence of new lesions. The overall response in patients without target lesions at baseline is determined by combining the response of the non-target lesions and the presence or absence of new lesions according to Table 11.1.9.b.

Table 11.1.9.a Time point response: patients with target (+/–non-target) lesions.

| Target lesions | Non-arget lesions | New lesions | Overall response |
| --- | --- | --- | --- |
| CR | CR | No | CR |
| CR | Non-CR/non-PD | No | PR |
| CR | Not evaluated | No | PR |
| PR | Non-PD or not all evaluated | No | PR |
| SD | Non-PD or not all evaluated | No | SD |
| Not all evaluated | Non-PD | No | NE |
| PD | Yes or No | Yes or No | PD |
| Yes or No | PD | Yes or No | PD |
| Yes or No | Yes or No | Yes | PD |

Table 11.1.9.b Time point response: patients with non-target lesions only.

| Non-target lesions | New lesions | Overall response |
| --- | --- | --- |
| CR | No | CR |
| Non-CR/non-PD | No | Non-CR/non-PD |
| Not all evaluated | No | NE |
| PD | Yes or No | PD |
| Yes or No | Yes | PD |

### Best Overall Response

Since only one response evaluation is performed in this study, the overall response is considered the best overall overall response.

If any response evaluation by imaging cannot be performed due to early symptomatic deterioration or death before the first response evaluation. In addition, if any response evaluation by imaging cannot be performed by the early termination of the protocol treatment due to toxicity or patient refusal before the first response evaluation, the best overall response should be NE.

### Pathological response evaluation

Pathological response evaluation for preoperative chemotherapy using resected tissue specimens is performed at each institution.

The specimens used for pathological response evaluation should be the H&E stained tissue sections of the largest plane in the longitudinal direction of the tumor, and the percentage of residual viable tumor cell is evaluated histopathologically. Grade 3 and Grade 4 are regarded as 'Effective' for preoperative chemotherapy. Mapping is recommended when determining pathological response.

**・ Grade 1: Viable tumor cells remain greater than 50%**

**・ Grade 2: Viable tumor cells remain > 10% and ≦ 50%**

**・ Grade 3: Viable tumor cells remain 10% or less**

**・ Grade 4: No viable tumor cells remain**

**・ Not Evaluable (NE): The pathological examinations cannot be performed for any reason or neither Grade can be determined**

 Note 1: Tumor cells with pyknosis, karyorrhexis, or karyolysis are judged to be non-viable cells. Tumor cells with eosinophilic cytoplasm, vacuolar degeneration, swelling of the nucleus are judged to be viable tumor cells.

Note 2: In the hisitopathological mapping of viable tumor cells, square measures of the area with extremely decreased cellularity due to chemotherapy and viable tumor cells distributing sparsely are multiplied by 1/10 or 1/20 as appropriate and should be added to the sum of square measures of the area with viable tumor cells.

## Definitions of analyses set

The analysis sets used in periodic central monitoring, interim analysis, and final analysis are defined as follows: The flow diagram below shows the relationships of analysis sets.


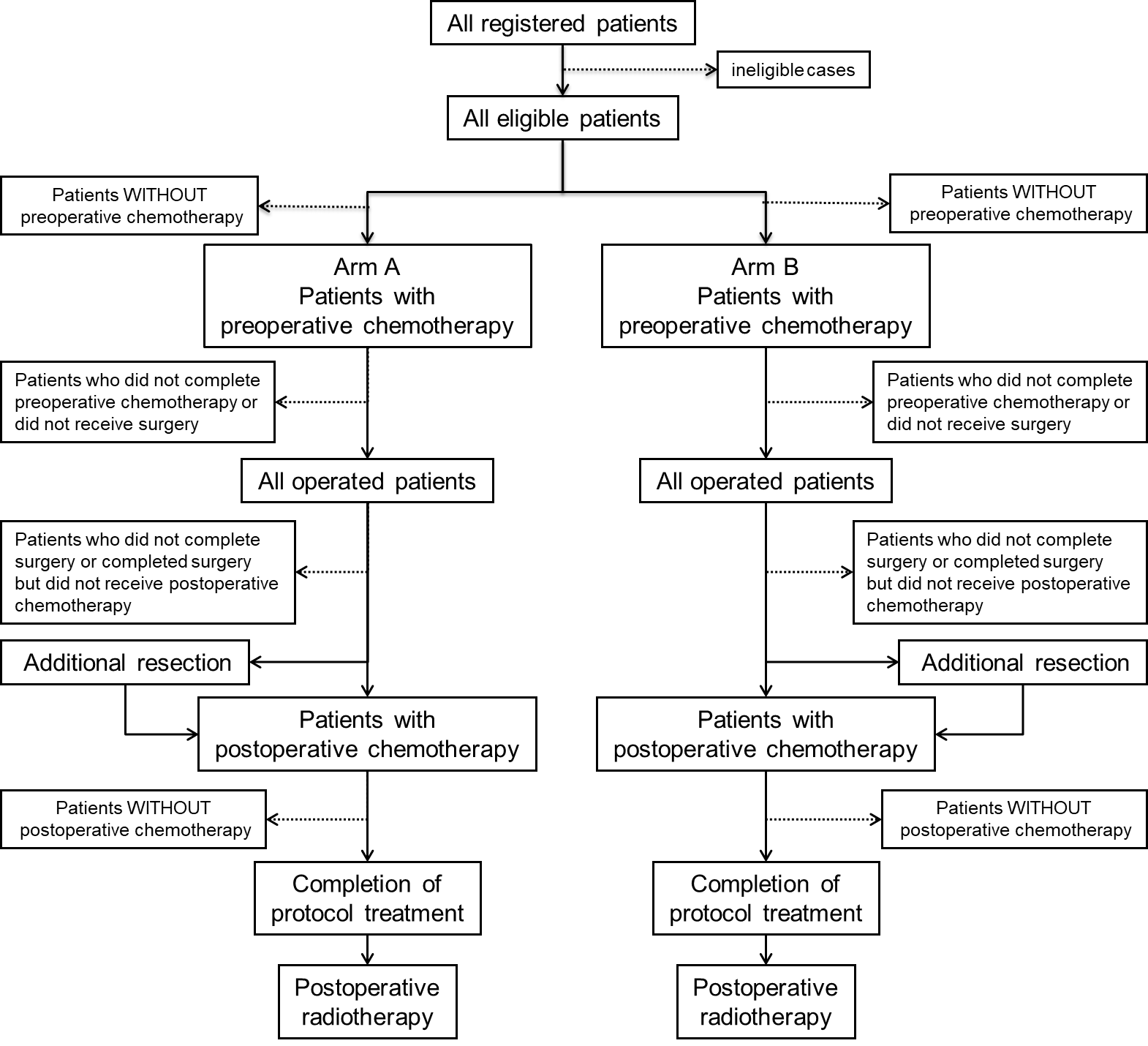


### All registered patients

Among the patients enrolled according to '5.1. Registration procedures', the group excluding duplicate registration and mis-registration is regarded as all registered patients.

### All eligible patients

The group excluding "ineligible cases (post hoc ineligibles, de facto ineligibles, violation of registration)" determined by group review from all registered patients is regarded as all eligible patients. Ineligible cases as judged by the investigator or sub-investigator alone are included in all eligible patients. Those considered not eligible according to the central pathological review only are not eligible and are included in all eligible patients.

### Eligible patients by central pathological review

Among all eligible patients, the group judged to be eligible as a result of the central pathological review is defined as the "Eligible patients by central pathological review".

### All treated patients

Among all registered patients, all patients who received some or all of the protocol treatments are regarded as all treated patients.

Data Center is allowed to determine "non-treated patients" who received no protocol treatment, and whether exclude them from the safety analysis under the consent by the Study Coordinator. Whether ineligible patient is included in the analysis sets is determined after reviewing the ineligibility by the Study Coordinator in consultation with JCOG Data Center.

### Patients with preoperative chemotherapy

Among all registered patients, patients who received part or all of preoperative chemotherapy are regarded as "patients with preoperative chemotherapy".

### Patients completed preoperative chemotherapy

Patient group excluding the patients who do not meet the criteria of preoperative chemotherapy completion (see 6.2.1.1)) from the patients completed preoperative chemotherapy is regarded as 'patients completed preoperative chemotherapy'.

### All operated patients

Group of the patients who completed preoperative chemotherapy or received surgery regardless of receiving or completing preoperative chemotherapy is regarded as 'all operated patients'. However, for patients who have undergone palliative resection, the decision will be made to be included in the analysis sets after discussion between Study Coordinator and JCOG Data Center.

### Patients with additional resection

Group of the patients who received additional resection among all operated patients is regarded as 'patients with additional resection'.

### Patients completed surgery

Group of the patients among all operated patients in which resection resulted in wide excision or marginal excision is regarded as 'patients with surgery completed'.

### Patients completed additional resection

Among all operated patients, the group in which additional resection was performed and resection resulted in wide excision or marginal excision is regarded as patients completed additional resection.

### Patients with postoperative chemotherapy

Among patients completed surgery and patients completed additional resection, the group who received a part or all of postoperative chemotherapy is regarded as patients with postoperative chemotherapy.

### Patients completed postoperative chemotherapy

Among patients with postoperative chemotherapy, the group who meet '6.2.1.3) Definition of postoperative chemotherapy Completion' is regarded as patients completed postoperative chemotherapy.

## Definition of endpoints

| Endpoint | Event (whichever is earlier) | | Censoring date |
| --- | --- | --- | --- |
| Overall survival  Overall survival(OS) | All deaths | - | Date of last survival confirmation |
| Progression-free survival  Progression-free survival(PFS) | All deaths | Progression/Relapse | Final date of clinically confirmed absence of progression |

### Overall survival

The duration from the date of registration to the date of death from any cause.

- Survivors are censored at the date of final survival confirmation (survival confirmation by telephone contact is permitted, but the fact that survival confirmation was performed should be recorded in the medical record).
- Patients lost to follow-up are censored at the last date of survival confirmation before lost to follow-up.

### Progression-free survival (PFS)

The duration from the date of registration to the date of the judgement of progression or death from any cause, whichever comes first.

- 'Progression' includes both imaging-based PD (Progressive Disease) in '11.1.9. Overall response' and progression that cannot be diagnosed by imaging studies (clinical progression). If the progression is judged based on imaging studies, the date of progression should be the date of imaging studies. The date of progression should be the date of clinical judgment in the case of clinical progression. In the case of PD according to response evaluation criteria but judged as clearly no progression clinically for example when the size of the tumors are very small, the event date of progression-free survival should be the date of judgement of PD. (In this case, clinical judgment should be prioritized to decide whether or not protocol treatment should be continued.) In addition, patients who are not PD according to response evaluation criteria but who are apparently clinically judged to have an progression are considered to be progression with precedence of clinical judgment. However, even if the tumor increases in size or progresses clinically after initiation of preoperative chemotherapy, it is not regarded as progression when the patient received tumor resection. In those cases, the date of event of progression-free survival event should be the date of judgement of relapse (if there is no residual tumor at surgery) or progression (if there is residual tumor at surgery). When tumor increases in size or clinical progression is observed after initiation of preoperative chemotherapy and tumor resection was not performed, the event date of progression-free survival should be the date of judgement of tumor increase in size or clinical progression. (because of progression that cannot be salvaged by surgery should be an event of progression-free survival)
- In survivors who are not judged to have progression, the progression-free survival should be censored at the last date at which no progression is confirmed clinically (last date of confirmation of survival without progression). (The confirmation of progression-free by imaging or specimen examination is not mandatory, and the confirmation of clinical progression-free by outpatient visit etc. is permitted, however, the confirmation by telephone contact alone is not permitted. If information on progression or progression-free is obtained from the medical institution, to which the registered patient is transferred or referred, receive and store a medical information form containing the evidence for diagnosis. In this case, telephone contact alone is not permitted).
- Events and censoring are treated similarly, if chemotherapy is terminated for reasons such as toxicity or patient refusal and other therapies are performed as post-study treatment. i.e., progression-free survival is not censored at the time of treatment termination or at the date of initiation of post-study treatment.
- When the diagnosis of progression is based on imaging, the relapse is not regarded as an event at the date of imaging study with "suspicious diagnosis of relapse", but regarded as an event at the later date of imaging study with "definite diagnosis of relapse"" If a progression is judged clinically not based on imaging, it is regarded as the event at the day on which the progression is judged.
- If a definitive diagnosis of relapse or new lesion is made by biopsy pathology, when diagnosis of relapse or new lesion is made clinically, clinical diagnosis of relapse is regarded as an event at the date of clinical diagnosis, and when a diagnosis of relapse is made by biopsy pathology without a clinical diagnosis of relapse, pathological diagnosis of relapse is regarded as an event at the date of biopsy.
- The incidence of second cancers (metachronous double cancers) should not be an event or censoring, and progression-free survival is continued until other events are observed.

### Proportion of completion of preoperative chemotherapy without progression

Of all registered patients, the percentage of patients who completed 3 courses of preoperative chemotherapy and whose best overall response (see '11.1.10. Best overall response') after preoperative chemotherapy is either CR, PR, or SD is defined as proportion of completion of preoperative chemotherapy without progression.

### Response proportion (response rate) of preoperative chemotherapy

Among all registered patients, the percentage of patients whose best overall response (see '11.1.10. Best overall response') after preoperative chemotherapy is either CR or PR is defined as the response proportion (response rate).

### Pathological response proportion (pathological response rate)

Proportion of patients with Grade 3 or Grade 4 based on "11.1.11 Pathological response evaluation" in all registered patients.

### Proportion of limb preserved

Proportion of patients with limb preservation after surgery in all registered patients whose tumor origin is limb.

### Tumor control proportion

Among all registered patients, the percentage of patients whose best overall response (see '11.1.10. Best overall response') after preoperative chemotherapy is either CR, PR, or SD is defined as the tumor control proportion.

### Incidence of adverse events (adverse reactions)

#### 1) During preoperative chemotherapy

Using patients with preoperative chemotherapy as the denominator, the frequency of the worst Grade during all courses are calculated by arm for each of the following adverse events (toxicities) according to CTCAE v4.0-JCOG:

- Laboratory tests: white blood cell decreased, neutrophil count decreased, platelet count decreased,

Increased blood bilirubin, aspartate aminotransferase increased (AST or GOT),

Alanine aminotransferase increased (ALT or GPT), creatinine increased,

Hypernatremia, hyperkalemia, hyponatremia, hypokalemia

- General disorders and administration site conditions: fever, edema limbs, fatigue
- Skin and subcutaneous tissue disorders: palmar-plantar erythrodysesthesia syndrome, skin hyperpigmentation
- Gastrointestinal disorders: diarrhea, nausea, oral mucositis
- Metabolism and nutrition disorders: anorexia,
- Infections and parasites: bronchial infection, pulmonary infection, soft tissue infection, bone infection
- Cardiac disorders: Supraventricular tachycardia, ventricular arrhythmia, left ventricular systolic dysfunction
- Blood and Lymphoid Disorders: anemia, febrile neutropenia
- Nervous system disorders: vertigo, dizziness, leukoencephalopathy, peripheral sensory neuropathy, peripheral motor neuropathy, decreased level of consciousness, seizures
- Respiratory, thoracic and mediastinal disorders: pneumonitis
- Renal and urinary disorders: hematuria

#### 2) During postoperative chemotherapy

Using patients with postoperative chemotherapy as the denominator, the frequency of the worst Grade during all courses are calculated by arm for each of the following adverse events (toxicities) according to CTCAE v4.0-JCOG:

- Laboratory tests: white blood cell decreased, neutrophil count decreased, platelet count decreased,

Increased blood bilirubin, aspartate aminotransferase increased (AST or GOT),

Alanine aminotransferase increased (ALT or GPT), creatinine increased,

Hypernatremia, hyperkalemia, hyponatremia, hypokalemia

- General disorders and administration site conditions: edema limbs
- Skin and subcutaneous tissue disorders: palmar-plantar erythrodysesthesia syndrome, skin hyperpigmentation
- Gastrointestinal disorders: diarrhea, nausea, oral mucositis
- Metabolism and nutrition disorders: anorexia,
- Infections and infestations: bronchial infection, pulmonary infection, soft tissue infection
- Cardiac disorders: supraventricular tachycardia, ventricular arrhythmia
- Blood and lymphatic system disorders: anemia, febrile neutropenia
- Nervous system disorders: dizziness, leukoencephalopathy
- Respiratory, thoracic and mediastinal disorders: pneumonitis
- Renal and urinary disorders; hematuria

**3) Intraoperative complications**

Using all operated patients as the denominator, the frequency of Grade of the following adverse events (surgical complications) are calculated by arm according to CTCAE v4.0-JCOG:

Vascular disorders: thromboembolic event

Injury, poisoning, and procedural complications: intraoperative arterial injury, intraoperative venous injury, intraoperative nervous system injury, intraoperative musculoskeletal injury

**4) Early postoperative complication (until 27 days after surgery (additional resection) or postoperative chemotherapy initiation date, whichever comes first)**

Using all operated patients as the denominator, the frequency of Grade of the following adverse events (surgical complications) are calculated by arm according to CTCAE v4.0-JCOG.

- General disorders and administration site conditions: fever, edema limbs
- Vascular disorders: peripheral ischemia, thromboembolic event
- Injury, poisoning and procedural complications: wound dehiscence
- Nervous system disorders: Nervous system disorders
- Infections and infestations: bone infection, joint infection, lung infection, bronchial infection, wound infection, soft tissue infection

**5) Late complications (after protocol treatment completion/termination)**

Using all registered patients as the denominator, the frequency of Grade of each following adverse events are calculated by arm according to CTCAE v4.0-JCOG.

- General and general disorders and application site conditions: edema limbs
- Cardiac disorders: supraventricular tachycardia, ventricular arrhythmias (if subjective symptoms or arrhythmias are absent, grade as Grade 0 and ECG is not mandatory)
- Respiratory, thoracic and mediastinal disorders: dyspnea, pneumonitis
- Nervous system disorders: peripheral sensory neuropathy, peripheral motor neuropathy
- Vascular disorders: peripheral ischemia

In the other adverse event (toxicitiy) than the above, the proportion of occurrence are not calculated unless a large number of specific adverse events are observed, since only Grade 3 or more non-hematological toxicity is reported in the Treatment Form.

### Incidence of respiratory toxicity

The percentage of patients in whom at least one of the protocoled treatments was initiated (all treated patients) is used as the denominator, and the percentage of patients in whom at least one adverse event of Grade 2 was observed in any one or more of the respiratory toxicities (bronchial infection, pulmonary infection, pneumonitis) before or after surgery is used as the numerator.

### Incidence of serious adverse events (adverse reactions)

#### 1) Incidence of Grade 4 non-hematologic toxicities

The proportion of the patients who had at least one Grade 4 non-hematological toxicities judged as protocol treatment-related (definite, probable or possible), which is listed in Section 11.3.8. or described in the free text field of the CRFs, out of all treated patients.

※ Non-hematological toxicity refers to adverse events other than those listed below in CTCAE v4.0-JCOG.

anaemia, bone marrow hypocellular, lymphocyte count decreased, neutrophil count decreaseda, white blood cell decreased, platelet count decreased, CD4 lymphocyte decreased.

#### 2) Proportion of early death

Proportion of all deaths during the protocol treatment or within 30 days from the last protocol treatment day among all treated patients. Causes of death irrespective of causality with protocol treatment.

#### 3) Proportion of treatment-related death (%TRD)

Proportion of all deaths judged as causally related (either definite, probable, possible) to the protocol treatment among all treated patients

### Proportion of surgical complications

The proportion of surgical complications is defined as the proportion of patients with at least one observed surgical complication listed in "7.1.4 Expected adverse reactions and complications by surgical resection" or other unexpected surgical complications out of all operated patients. If additional resection is performed, it should be evaluated in conjunction with the initial surgery. Proportions of intraoperative complications, early postoperative complications, and late complications will be calculated by treatment arm.

# Statistical consideration

Methods for statistical analysis are as follows: In addition, the details required for conducting specific analyses are specified in the statistical analysis plan prepared separately prior to the analysis, and in documents that clarify the endpoint definition, etc. If substantial changes occur in statistical analyses as described below, follow the policy set out in "13.6. Protocol Changes." Facilities will be contacted by "14.1. Periodic Monitoring" for missing or abnormal results, and data will be collected or not by review by the Study coordinator based on the results of inquiries to the institution in accordance with the policies stipulated in "14.1.2. eligible (eligibility/ineligibility)" and "14.1.3. Protocol Deviations/Violations". The treatment of missing values and abnormal data that cannot be dealt with by the definition and analysis method of each endpoint, as set forth in the "11. Response Assessment and Definition of Endpoints" and "12. Statistical consideration", below, is set forth in the statistical analysis plan described above.

## Primary Analysis and Decision Criteria

The primary analysis of this study is to confirm that the standard treatment arm, Arm A (ADM + IFO), versus Arm B (GEM + DOC), can be denied (non-inferiority) to be below the acceptable range in terms of primary endpoint, overall survival.

We conclude that the study treatment, the GEM plus DOC combination, is a more useful treatment when the experimental treatment arm can prove to be non-inferior to the standard treatment arm in overall survival and the safety endpoint shows the benefit of the study treatment and the point estimate of HR for progression-free survival is below 1.4. We conclude that the standard of care, ADM plus IFO therapy, continues to be a useful treatment if noninferiority cannot be demonstrated, if noninferiority shows no treatment benefit at the safety endpoint, or if the point estimate of HR for progression-free survival is over 1.4. Based on the background presented in "2.4.2. Clinical Hypothesis and Rationale for sample size," the decision included a progression-free survival criterion, as the patient had a major disadvantage and could not accept the experimental treatment if progression-free survival appeared to be substantially inferior to the standard treatment arm.

This is a study to test for non-inferiority; therefore, a one-sided test is performed. Considering that target population is rare cancer, the study-wise significance level is one-sided 10%. Significance levels and corresponding confidence coefficients used to test the primary hypothesis is based on the multiplicity adjustment associated with the interim analysis. Other than testing the primary hypothesis, 95% confidence intervals will be calculated for summary purposes.

The primary analysis will be based on a stratified Cox proportional hazards model for all enrolled patients, stratified by adjustment factors (first relapse [index vs. recurrence], site [extremity vs. trunk]) except for institution, including treatment arm as a covariate, and tested using the confidence interval of the hazard ratio for the standard treatment arm of the experimental treatment arm. Wald-type confidence interval (CI) is used. The non-inferiority margin for group comparisons is a hazard ratio <1.61 for the experimental treatment arm versus the standard treatment arm. In other words, if the upper confidence limit of the hazard ratio is below 1.61 based on the adjustment for multiplicity, statistically significant non-inferiority is judged to be demonstrated. However, if it is assumed that a stratified analysis by Cox proportional hazards model cannot be performed appropriately using the 2 factors, such as when the number of patients and events in each stratum is small, the adjustment factor will be handled in an analysis plan prepared without information on group comparisons before conducting a confirmatory analysis with group comparisons.

Test for superiority is performed only if the primary analysis demonstrates non-inferiority in overall survival. The significance level shall be the same as that used for non-inferiority testing, since no adjustment for multiplicity is required according to the closed testing procedure. Confirmation of superiority is based on hazard ratios from stratified Cox regression stratified by adjustment factors (first relapse [first recurrence vs. recurrence], and location [limb vs. trunk]) except for institution in all enrolled patients, and superiority is judged to be demonstrated if the upper limit of the confidence interval is less than 1. There are some options to test superiority using the log-rank test, but the hazard ratio used to test non-inferiority and its confidence interval are to be used by Cox regression with emphasis on simplicity. As a reference, the results of comparisons by the non-stratified log-rank test are also presented, but the main decision is made by the results based on the upper confidence limit of the hazard ratio. Sensitivity analyses will also be done in all eligible patients as well as all treated patients as appropriate.

Estimates such as cumulative survival curves, median survival times, and annual survival rates are performed using Kaplan-Meier method. Brookmeyer and Crowley methods are used to obtain 95% confidence intervals for median survival. Greenwood's formula is used to obtain 95% confidence intervals for annual survival rates. Hazard ratios with their 80% confidence intervals and 95% confidence intervals for treatment effect between arms will be obtained using the stratified Cox proportional hazards model described above as an estimate of treatment effect. Cox regression adjusted for biased background factors as appropriate is performed.

The primary analysis results will be summarized by the Data Center as the Primary Analysis Report and submitted to the study coordinator, principal physician, group chair, Data and Safety Monitoring Committee and JCOG chair.

The principal investigator/study coordinator summarizes the content of the main analysis report, prepares a "Clinical Study Report" summarizing the conclusions, problems, interpretations and discussion of the results, and future policies of the entire study, and submits it to the Data and Safety Monitoring Committee and JCOG chair with approval from the Group chair and the Head of JCOG Data Center.

## Planned enrollment, enrollment, and follow-up periods

Based on the background presented in the "2.4.2. Clinical Hypothesis and Rationale for Sample size", the 3-year survival rates of Arm A and Arm B are assumed to be 85% and 87%, respectively (hazard ratio of Arm B to group A = 0.857). The non-inferiority margin is set at 8% (non-inferiority margin of 1.61 in terms of hazard ratio).

Using Schoenfeld & Richter methodological ^(46)^, the number needed to test for non-inferiority of experimental arm to the standard treatment arm is 65 in each arm and 130 in both arms (33 events required), 6 years of enrollment, 3 years of follow-up, alpha = 10% (one-sided), power 70%, non-inferiority margin of hazard ratio 1.61. The number of required patients (number of required events) when the 3-year survival rate in the standard treatment group differs from the assumption is shown in Table 12.2 below.

Table 12.2. 3-year survival rates in both groups and thenumber of required patients corresponding to power

| 3-year survival rate | | Non-inferiority margin of HR | Total number of required patients for each power in both groups  (number of required events ) | | |
| --- | --- | --- | --- | --- | --- |
| Standard treatment group  (ADM+IFO) | Experimental treatment arm  (GEM+DOC) |  | 65% | 70% | 75% |
| 80% | 82.6% | 1.61 | 86 (28) | 100 (33) | 118 (38) |
| 85% | 87% |  | 110 (28) | 130 (33) | 152 (38) |
| 90% | 91.4% |  | 160 (28) | 188 (33) | 222 (38) |

(*) Hazard ratios for the experimental treatment arm versus the standard treatment arm, corresponding to the null hypothesis

That is, 33 events are required to achieve a power of 70% or more with the assumption settings.

Since the efficacy and safety data for high-grade soft tissue sarcomas in Japan are not sufficiently available for the GEM+DOC combination therapy, which is the experimental treatment arm in this study, this study is designed as a phase II/III design to confirm the efficacy and safety in the phase II part, and an interim analysis will be conducted using the rate of complete progression free of neoadjuvant chemotherapy as the endpoint during the enrollment period. Based on the rationale given in "2.4.2 Clinical Hypothesis and Rationale for Sample sizse", a threshold of 65%, an expected value of 85%, an alpha = 10% (one-sided), and a power of 80% would result in 28 patients per arm needed to be analysed by exact method based on a binomial distribution.

Based on these findings, the following will be established in view of some cases of loss to follow-up.

Planned enrollment: 70 patients in each group and 140 patients in both groups

Planned enrollment time: 6 years, follow-up time: 3 years after completion of enrollment (but follow-up until 5 years after completion of enrollment after primary analysis)

The analysis period is expected to be 1 year, and the total study period is 12 years.

Consider redesigning the sample size when there is a large deviation from prior assumptions, such as when periodic monitoring clearly indicates a better prognosis than assumed, the clinically meaningful differences will also be reviewed and redesigned under blind prior to the conduct of the analysis.

## Interim analysis

### Purpose and Timing of the Interim Analysis

Interim analyses will be conducted twice to determine if the primary objective of the study has been achieved during the study period.

The first interim analysis will assess whether the experimental treatment (GEM plus DOC) arm has adequate efficacy and safety after the planned number of enrolled in the phase II part is obtained and will determine whether it is appropriate to continue the study as a phase III trial. The second interim analysis will be conducted early after the completion of enrollment in the phase III par to determine whether follow-up for the planned period will continue. In any case, the study will be terminated if the primary objective of the study is determined to be achieved, and the study results will be promptly published at the conference and in the paper.

The first interim analysis will investigate the rate of completion of neoadjuvant chemotherapy in the experimental treatment (GEM + DOC) arm at the time when 28 patients in the GEM + DOC arm is enrolled (phase II part). The second interim analysis will be conducted at the time of completion of enrollment and completion of protocol treatment for all enrolled patients and will be tailored to periodic monitoring at the time that may be appropriate after consultation with the Data Center and the Study Corrdinator.

If the progress of the study is as expected, the expected number of events at the time of the interim analysis under the assumptions given in Section 12.2 is expected to be 19 if the second interim analysis is conducted at the end of enrollment (6 years after enrollment).

### Method of interim analysis

Interim analyses will be conducted by the JCOG Data Center.

When the first interim analysis (phase II part) reaches 28 subjects who are enrolled in the GEM + DOC combination therapy group, which is the planned number of patients enrolled in the phase II part, the Data Center will inform the Study coordinator and anticipate the time of the analysis (approximately 6 months later) when data from the interim analysis can be obtained. The Data Center will encourage documentation and inquire about unclear points in the case report form so that appropriate interim analyses can be conducted at the expected time of analysis in cooperation with the Study coordinator. The Study coordinator will conduct a review (CRF review) of reporting forms prior to the analysis to determine data such as response assessments used for the analysis.

If it is obvious that the threshold is exceeded in 28 patients enrolled in the GEM+DOC combination therapy group when the planned enrollment in the phase II part is reached, i.e. more than 22 patients (78.6%) with complete progression-free preoperative chemotherapy have been identified, the registration will not be stopped and JCOG Data Center will inform the Study coordinator that the results will be compiled in an interim analysis report and presented to the Data and Safety Monitoring Committee.

On the other hand, if it is not obvious that the threshold is exceeded in 28 patients enrolled in the GEM plus DOC group at this time, i.e. only 21 or fewer patients with complete preoperative chemotherapy progression-free can be identified, enrollment is stopped until more than 22 patients with complete preoperative chemotherapy progression-free can be confirmed. If progression-free completion of neoadjuvant chemotherapy of at least 22 patients can be confirmed, the Data Center will summarize the results in an interim analysis report and present them to the Data and Safety Monitoring Committee. Enrollment is resumed after approval for continuation of the study was obtained by the Data and Safety Monitoring Committee.

In both cases, the Data Center calculates the preoperative chemotherapy progression-free completion rate in the GEM + DOC combination arm using the preoperative chemotherapy progression-free completion established by the Study corrdinator, and examines at a one-sided significance level of 10% whether the null hypothesis H0 (true response rate of 65% or less) can be rejected based on the required preoperative chemotherapy progression-free completion rate. No comparisons were made between groups, and the proportion of patients who completed neoadjuvant chemotherapy without progression in the control ADM+IFO group is also presented as a reference value.

The Data and Safety Monitoring Committee will judge the appropriateness of transition to the Phase III part and whether or not to continue enrollment based on the results of the interim analysis report. In principle, if the null hypothesis is rejected, the investigator judges that "the treatment regimen in the GEM plus DOC group is likely to achieve the expected effect," and judges that the transition to the phase III part is reasonable. On the other hand, if the null hypothesis is not rejected, the study will be discontinued as a rule based on the judgment that the treatment regimen in the GEM plus DOC group is unlikely to achieve the expected effect.

The second interim analysis (phase 3 part) uses Lan & DeMets's alpha spending functions to adjust the multiplicity of the test in the second interim analysis and the final analysis to keep the study-wise alpha errors at one-sided 5.0% and investigate the statistical significance for differences in overall survival between arms. As α spending functions, we use O'Brien & Fleming types.

For details of the second interim analysis, the statistical staff in charge of the group at the Data Center will prepare the analysis plan by the time of the interim analysis. Actual interim analyses will be performed by statistical staff who are not in charge of the group and an interim analysis report will be prepared.

In the second interim analysis, the hazard ratio for treatment effect and the confidence interval corresponding to the level of significance at the time of the analysis is calculated in the manner specified in "12.1 Primary Analysis and Decision Criteria," and the overall survival in Arm B (GEM + DOC combination therapy) exceeded that in Arm A (ADM + IFO therapy). If the upper limit of the confidence interval of the hazard ratio adjusted for multiplicity was below the acceptable hazard ratio of 1.61, statistically significant non-inferiority was determined.

If non-inferiority is demonstrated, the superiority will continue to be analyzed. When the upper limit of the confidence interval of the hazard ratio adjusted for multiplicity is below 1, we judge it to be statistically significantly superior.

Decision criteria based on the results of the interim analysis is following.

- The trial will continue if the treatment arm (GEM + DOC) surpasses the overall survival but non-inferiority is not demonstrated for the standard arm (ADM + IFO therapy) or if non-inferiority is demonstrated but no superiority is demonstrated.
- The trial will be terminated (termination by efficacy) if the trial treatment arm (GEM + DOC combination) proves non-inferior to the standard arm (ADM + IFO combination) in terms of overall survival and further proves to superiority.
- If the overall survival of the experimental treatment group (GEM + DOC combination) is below that of the standard treatment group (ADM + IFO combination), the study termination will be considered comprehensively without limitation to statistical judgment such as testing. If the point estimate of the hazard ratio exceeds the non-inferiority margin of hazard ratio (hazard ratio = 1.61), the study will be terminated (futility stopped).

In the standard non-inferiority trial, if non-inferiority is demonstrated at the end of enrollment, the study is terminated by efficacy at that time. However, the interim analysis at the end of enrollment (at the 2nd interim analysis) is expected to show a small number of events in this trial, and it is likely that the non-inferiority may not be demonstrated in the follow-up results even if non-inferiority is demonstrated and efficacy is discontinued in the interim analysis depending on the shape of the survival curve. Therefore, the trial is terminated if it is proven to superiority as well as non-inferiority in overall survival of the experimental treatment arm.

### Interim analysis Reporting and review of the results

The results of the interim analysis will be submitted to the Data and Safety Monitoring Committee by the Data Center as an interim analysis report and reviewed for the acceptability of continuation of the study and for publication of the results. The Data and Safety Monitoring Committee considers whether to continue the study by written review in the first interim analysis and by the meeting in the second interim analysis, and recommends whether to continue the study and whether to publish the results to research representatives or group representatives based on the review results.

Members of the Data and Safety Monitoring Committee of the relevant group are not included in the review. Unless the results of the interim analysis make recommendations for termination of the study from the Data and Safety Monitoring Committee, the principal investigator, the sutyd corrdinator, researchers at participating sites, group chair, and the Group Secretary of the study cannot know the results of the interim analysis until the primary analysis is completed.

When the Interim Analysis Report has been reviewed by the Data and Safety Monitoring Committee to recommend termination or change of all or part of the study, the principle investigator and group chair review the recommendations and decide whether to discontinue or change some of the study.

If the study is terminated or part of the study is changed, the principal investigator and group chair shall submit in written form a request to the Data and Safety Monitoring Committee for permission to discontinue the study or a request to revise the protocol. Following approval by the Data and Safety Monitoring Committee, the principal investigator may discontinue the study or change part of the study.

The principle investigator and group chair can disagree with the recommendations of the Data and Safety Monitoring Committee, but if they fail to adjust their opinions with the Data and Safety Monitoring Committee, they will ultimately follow the instructions of JCOG chair.

If the study is terminated, the subsequent follow-up period will be 5 years from last enrollment.

If the interim analysis resulted in study termination, the interim analysis will be the primary analysis of the study. In cooperation with the principal investigator and the study corrdinator, the Data Center will conduct analyses necessary for imputation of incomplete data and publication of results, focusing on the results of the interim analysis in question, and promptly prepare the main analysis report and submit it to the Group and the Data and Safety Monitoring Committee.

## Analysis of Secondary endpoints

Secondary endpoints analyses will be conducted to provide a supplementary discussion of the primary analysis results of the study. Because the analysis of secondary endpoint is exploratory, no multiplicity adjustments are made. Comparisons between arms are made where appropriate, note that when the results of the group comparisons are not significant, they do not mean that there is no difference between the two arms.

### Analysis of safety secondary endpoints

Among Secondary endpoints, the safety endpoints are the incidence of adverse events, the incidence of serious adverse events, the incidence of respiratory toxicity, and the incidence of surgical complications, which are, in principle, routine monitoring items ("14.1. Periodic monitoring").

The rate of serious adverse events and surgical complications are expected to be similar or lower in the study treatment group compared with the standard treatment group. We expect the adverse event rate to be lower in the study treatment group compared to the standard treatment group. The incidence of adverse events will be summarized for each incidence of adverse events, and the incidence of Grade3 or higher will be calculated. For adverse events other than laboratory data, the incidence of Grade 2 or higher is also calculated. Grade 4 non-hematologic toxic incidence, early death, and treatment-related death rates, which are serious adverse events, are reported in periodic monitoring reports with patiens identification numbers and details. The incidence of Grade 4 non-hematologic toxicities, early death, and treatment-related death will be calculated at the time of interim analysis, primary analysis, and final analysis. Exact method based on binomial distribusion is used to estimate confidence interval for the propotion. Neither of these endpoints is judged based on statistical tests, but comparisons are made between arms using Fisher's exact test, as appropriate.

The respiratory toxicity incidence is assumed to be high, especially in the study treatment group (GEM + DOC combination), but we expect that it will not greatly outperform the standard treatment group (ADM + IFO combination). Exact method based on binomial distribusion is used to estimate confidence interval for the propotion. Comparisons between arms will be made using Fisher's exact test where appropriate.

### Analysis of efficacy secondary endpoints

Among Secondary endpoints, the efficacy endpoints were progression-free survival, response rate of neoadjuvant chemotherapy, pathological response rate, limb salvage rate (limb development), and tumor control rate (limb trunk development), which will only be analyzed in the second interim analysis, main analysis, and final analysis.

Since progression-free survival is positioned as a surrogate endpoint of overall survival, we expect the combined GEM+DOC group to be non-inferior to the combined ADM+IFO group. Analysis will be done in all eligible patients. Comparisons by all eligible patients, except for ineligible patients, which were determined after enrollment by the Group as necessary, will also be performed as a sensitivity analysis.

Response rate, pathological response rate, limb salvage rate (limb development), and tumor control rate (limb trunk development) of neoadjuvant chemotherapy, all of which we expect GEM plus DOC combination therapy to be equivalent to ADM plus IFO combination therapy.

Fisher's exact test will be used for comparisons of rates, and binomial distribution-based exact confidence intervals will be used for interval estimates. Estimates such as cumulative progression-free survival curves, median progression-free survival times, and annual progression-free survival rates are performed using Kaplan-Meier method. Brookmeyer and Crowley methods are used to obtain 95% confidence intervals for median survival. Greenwood's formula is used to obtain 95% confidence intervals for annual progression-free survival rates. Hazard ratios and their 95% confidence intervals for treatment effects between arms will be calculated using Cox's proportional hazards model as an estimate of treatment effect. Cox regression adjusted by imbalance background factors in addition to adjustment factors will be performed as needed.

## Final analysis

After completion of the follow-up period, analysis will be performed for all endpoints after the final monitoring confirmed the data.

No comparisons in secondary endpoints for efficacy and primary endpoint are made at any other time except when stated in the protocol or permitted by the Data and Safety Monitoring Committee.

The final analysis results will be summarized by the Data Center as a final analysis report and submitted to the study coordinator, principal investigator, group chair, group secretary, Data and Safety Monitoring Committee, and JCOG chair.

The principal investigator/study corrdinator shall summarize the content of the Final Analysis Report, prepare a "Clinical Study Report" (if the Principal Analysis Report has previously prepared a "Clinical Study Report," an additional "Clinical Study Report"), which summarizes the conclusions of the entire study, problems, interpretations and discussions of the results, and future policies from a clinical standpoint (the "Clinical Study Report") and submit it to the Data and Safety Monitoring Committee and JCOG chair with the approval of the Group chair and the Director of JCOG Data Center, with the approval of the Group chair and the Director of JCOG Data Center.

Approval of the clinical study report by the Data and Safety Monitoring Committee shall be considered as "end of the study."

## Exploratory analysis

To investigate the interaction between treatment effect and the subpopulation, subgroup analyses will be conducted exploratory with respect to the following factors: Because these analyses are not adequately powered and do not adjust for multiplicity, the results of each subgroup analysis should be interpreted as exploratory.

Factors planned for subgroup analysis

- Age (<40 years/40 years or older)
- Age (<50 years/50 years or older)
- PS (0/1)
- ・ Sex (male/female)
- Degree of tumor differentiation (Score 2/3).
- Degree of necrosis (Score 0/1/2).
- Nuclear division count (Score 1/2/3).
- Histological grade (Grade 2/3).
- Grade at pathological diagnosis (Grade 2/Grade 3) with MIB1 values.
- Scheduled procedure (extensive or amputation/margin or intralesional resection)
- Scheduled procedure (wide or amputation/margin/intralesional resection)

The following subgroup analyses will also be performed, but it is important to note that these are outcomes for which treatment was given after randomisation (interim variables), and therefore the results can be interpreted in different ways than results from other factors.

- Margin assessment (extensive/marginal/intrafocal)
- Histological margins (positive/negative margins)
- Postoperative radiation (with/without)
- Completed (completed/discontinued) protocol treatment
- Completed neoadjuvant chemotherapy (completed/discontinued)

## Premature withdrawal from the trial

In this study, early termination of the study may occur in the following cases:

1. Early termination due to discontinuation of interim analysis
2. Early termination due to adverse events
3. Early termination due to poor enrollment
4. Early termination due to other reasons

### Early termination by interim analysis

In this study, based on the criteria described in "12.3. Interim Analysis," recommendations for early termination of the study may be issued at the interim analysis review by theData and Safety Monitoring Committee. If the Data and Safety Monitoring Committee provides recommendations for early termination of the study, the principle investigator and group chair will review the recommendations and decide whether to terminate the study early.

### Early termination due to adverse events

A randomized controlled trial (JCOG0104) of GEM+DOC and DOC for Recurrent Non-Small Cell Lung Cancer, conducted by JCOG Lung Cancer Study Group, used the same GEM and DOC as the treatment in this group, with Grade 3/4 interstitial pneumonia occurring in 12.3% (8 cases) and treatment-related death in 3 cases, leading to premature discontinuation. Therefore, the risk of developing interstitial pneumonitis should be considered in this study, and the presence or absence of interstitial pneumonia should be regularly evaluated. Specifically, it was considered unacceptable that the incidence of Grade 3 or higher interstitial pneumonitis in the study treatment group exceeded the 7% incidence of pulmonary toxicities reported by Maki et al. In addition, treatment-related deaths should not exceed 2 in any of the two groups. When five cases of Grade 3/4 interstitial pneumonitis occur in the study treatment group, immediate enrollment is suspended and the Efficacy and Safety Evaluation Committee is advised to withdraw from the study. When one or fewer treatment-related deaths occur in each group, the patient will be reported to the Data and Safety Monitoring Committee for adjudication. In principle, enrollment will continue until the results of the review are available.

On the other hand, the standard treatment ADM + IFO combination therapy is also suspended from enrollment when 5 cases of Grade 3/4 encephalopathy or cardiotoxicity occur because of the possibility of encephalopathy or cardiotoxicity, and the Data and Safety Monitoring Committee is advised to discontinue the study.

### Early termination due to poor enrollment

If the patient enrollment pace is significantly worse than at the time of planning, early termination of the study may be advised by the Data and Safety Monitoring Committee. If early termination recommendations are issued by the Data and Safety Monitoring Committee due to poor enrollment, the principle investigator and group chair will review the recommendations and decide whether to terminate the study early.

### Early termination due to other reasons

12.8.1.～12.8.3. If other reasons except for 12.7.3., it is considered difficult to continue the study, the principle investigator will submit the request for early termination of the study to the Data and Safety Monitoring Committee. If the Data and Safety Monitoring Committee recommends early termination of the study based on the submitted data, the procedure for early termination of the study will be progressed.

## Procedures after Early termination of the Study

If the principle investigator accepts the recommendations made by the Data and Safety Monitoring Committee based on Section 12.8., he/she will promptly submit a notification to the Data and Safety Monitoring Committee that early termination of the study will be performed

The principle investigator will submit a termination notification to the Certified Review Board within 10 days of the date they decide to terminate the study early. If the study falls into a specified clinical trials under the Clinical Trials Act, the principle investigator shall submit a discontinuation notification to the Certified Review Board within 10 days of the date on which the study was decided to be prematurely discontinued, as well as submit a specified clinical trials discontinuation notification to the MHLW.

The principle investigator promptly informs the investigator of the decision to terminate the study early in writing, and the investigator who has received a report of early termination of the study will report in writing that the study was prematurely terminated to the institution's manager without delay.

If the study is terminated early, JCOG Data Center will promptly initiate the development of the primary analysis report or final analysis report. Subsequent follow-up will be 5 years from last enrollment.
